# Multifunctional Nanodiamond Conjugate With a Tumor‐Specific EGFR‐Targeting Peptide and Photoactivated CO Release for Improved Therapeutic Efficacy in Head and Neck Cancers

**DOI:** 10.1002/smll.74355

**Published:** 2026-07-01

**Authors:** Harsh Nitin Dongre, Elisabeth Mayerhoefer, Julia Puck, Himalaya Parajuli, Lorena Larios Salazar, Paul Johan Høl, Ridhima Das, Patrick Roth, Christian Arvei Moen, Ulrich Schatzschneider, Line Bjørge, Daniela Elena Costea, Anke Krueger

**Affiliations:** ^1^ The Gade Laboratory for Pathology Department of Clinical Medicine University of Bergen Bergen Norway; ^2^ Centre for Cancer Biomarkers Faculty of Medicine University of Bergen Bergen Norway; ^3^ Institute of Organic Chemistry University of Stuttgart Stuttgart Germany; ^4^ Institute of Organic Chemistry Julius‐Maximilians‐Universität Würzburg Würzburg Germany; ^5^ Department of Clinical Medicine University of Bergen Bergen Norway; ^6^ Biomatlab, Department of Orthopaedic Surgery Haukeland University Hospital Bergen Norway; ^7^ Institute of Inorganic Chemistry Julius‐Maximilians‐Universität Würzburg Würzburg Germany; ^8^ Department of Urology Haukeland University Hospital Bergen Norway; ^9^ Department of Obstetrics and Gynaecology Haukeland University Hospital Bergen Norway; ^10^ Department of Pathology Haukeland University Hospital Bergen Norway; ^11^ Center for Integrated Quantum Science and Technology University of Stuttgart Stuttgart Germany

**Keywords:** cancer treatment, diamond nanoparticles, nanocarbon, photoCORM, stimuli‐responsive targeted drug delivery, surface chemistry

## Abstract

The targeted and stimuli‐responsive delivery of drugs for the treatment of cancer using functionalized nanoparticles is a promising avenue for therapeutic applications. However, many of these conjugates are limited by either low biocompatibility, poor targeting efficiency or complex requirements for the response to stimuli. Here we report a highly efficient nanodiamond conjugate for the light induced delivery of carbon monoxide, a potent anti‐tumor agent. Nanodiamond particles are well‐known for their high biocompatibility, rich surface chemistry, and low cytotoxicity. Using co‐functionalization with an EGFR‐targeting peptide and a manganese carbonyl complex as PhotoCORM, the efficient delivery of CO to cancer cells is demonstrated with spatiotemporal resolution both in vitro and in vivo using blue‐light photoactivation. In vitro experiments using head and neck cancer models show significant cancer‐specific cytotoxicity only upon blue‐light exposure, whereas limited toxicity was observed toward non‐cancerous and pre‐malignant cells. Further in vivo experiments demonstrate the significant suppression of tumor growth and lymph node metastasis in a head and neck cancer xenograft mouse model while maintaining excellent biocompatibility. This paves the way for the application of multifunctional, nanodiamond‐based delivery tools in the stimuli responsive treatment of tumors.

## Graphical abstract

A multifunctional nanodiamond conjugate carrying a blue light‐activated carbon monoxide‐releasing molecule and an EGFR‐targeting peptide enables efficient, targeted CO delivery in vitro and in vivo. Upon photoactivation, the particles induce cancer‐specific cytotoxicity and significantly suppress tumor growth and lymph node metastasis in a head and neck cancer xenograft model.

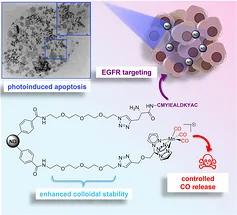

## 1 Introduction

Nanodiamond (ND) particles have been identified as one of the most promising materials for biomedical applications due to their high biocompatibility, low toxicity, and facile chemical surface modification [1, 2, 3]. Applications of ND include sensing of metabolic intermediates [4, 5], study of speciation of drugs [6] and pollutants [7], determination of local temperature [8, 9, 10], tissue engineering application [11, 12, 13], gene therapy [14, 15, 16], and the delivery of drugs. The latter was shown to have great potential for a broad range of anticancer drugs such as doxorubicin [17], paclitaxel [18], cis‐platin [19], and oligonucleotides [20] as well as anti‐infective drugs such as tetracycline [21]. Its advantage is the efficient uptake of otherwise water‐insoluble drugs, the sustained release from the ND drug conjugates as well as the benign behavior of this nanocarrier [1, 2, 3, 22]. The uptake and biocompatibility of ND‐drug conjugates can be controlled by appropriate surface functionalization of the ND particles. To this end, covalent and non‐covalent approaches have been reported [23, 24, 25]. For instance, different polymeric coating have shown great potential for the formation of “stealth” ND materials and for the formation of highly soluble ND conjugates with little agglomeration [26, 27, 28]. Also, covalent approaches for the improved uptake, colloidal stability, and highly defined binding of the drug or drug precursor molecules have shown their applicability [29, 30].

In recent years, it became evident that drug delivery can be significantly improved with regard to specificity and efficacy by using targeting approaches. ND particles have shown potential in targeted drug delivery (co‐delivery of multiple drugs), and stimuli‐responsive release [29, 31]. For instance, dual‐ligand‐functionalized NDs have been developed to simultaneously recognize cancer cell‐specific surface markers and target the mitochondria, effectively overcoming multidrug resistance in vitro and in vivo [32]. Such designs illustrate how the combination of molecular recognition and specific targeting can open new avenues for precision nanomedicine [29, 32, 33].

Most of the reported studies for drug delivery with NDs are based on non‐specific delivery and cellular uptake. Nevertheless, several studies on the grafting of targeting moieties on NDs and their use in sensing and drug delivery have been reported [34, 35]. These include targeting of receptors on tumor cells with small molecules [36, 37], peptides [38, 39], and antibodies [40, 41] as well as specific locations inside the cell, such as mitochondria [32] and the cell nucleus [42]. In drug delivery, these approaches have been used to achieve localized release and action of drugs such as doxorubicin and other antitumor drugs, which suffer from low specificity, poor solubility, and severe side effects in their typical formulations [43]. Stimuli responsive release using pH or thermal stimuli has also been demonstrated with targeted ND‐drug conjugates [44, 45].

Here we report for the first time the combination of efficient targeting of a clinically relevant receptor, epidermal growth factor receptor (EGFR), which is overexpressed in several cancer types, including head and neck squamous cell carcinomas (HNC) [46] with a photoactivated carbon monoxide‐releasing molecule (PhotoCORM) [47, 48, 49, 50] anchored on detonation ND as the drug delivery vehicle. This platform enables highly localized photochemotherapy to topically accessible/treat squamous cell carcinomas such as head and neck cancers.

## 2 Results and Discussion

### 2.1 Synthesis and Characterization of PhotoCORM‐Functionalized ND particles

Building on our previous work [49] in which we developed PhotoCORM‐functionalized ND particles capable of efficient CO release, we aimed to optimize this system further for both in vitro and in vivo applications. Thus, the short aromatic azide spacer for covalent immobilization of alkyne‐modified CO‐releasing complex [48, 51] [Mn(CO)_3_(tpm)]PF_6_ (**CORM**) with tpm = tris(pyrazolyl)methane on ND was replaced by a flexible hydrophilic linker to facilitate colloidal stability and biocompatibility in biological environments. Moreover, an EGFR‐targeting peptide (**anti‐EGFR pep**) was conjugated, in combination with the photoactivable **CORM**, to ND carrying the improved linker architecture, thereby ensuring selective targeting of malignant epithelial cells overexpressing EGFR. The selected EGFR‐targeting peptide sequence CMYIEALDKYAC was modified at the *N*‐terminus by a linking amino acid with a propynyl group. This peptide sequence has been shown to effectively bind to EGFR to increase biodistribution in EGFR‐overexpressing tumor cells [52, 53]. Additionally, the choice of side chain grafting conserves the zwitterionic nature of the peptide after the conjugation step, which has been shown to be beneficial for colloidal stability, efficient cellular uptake, and the prevention of protein corona formation [54].

**SCHEME 1:**
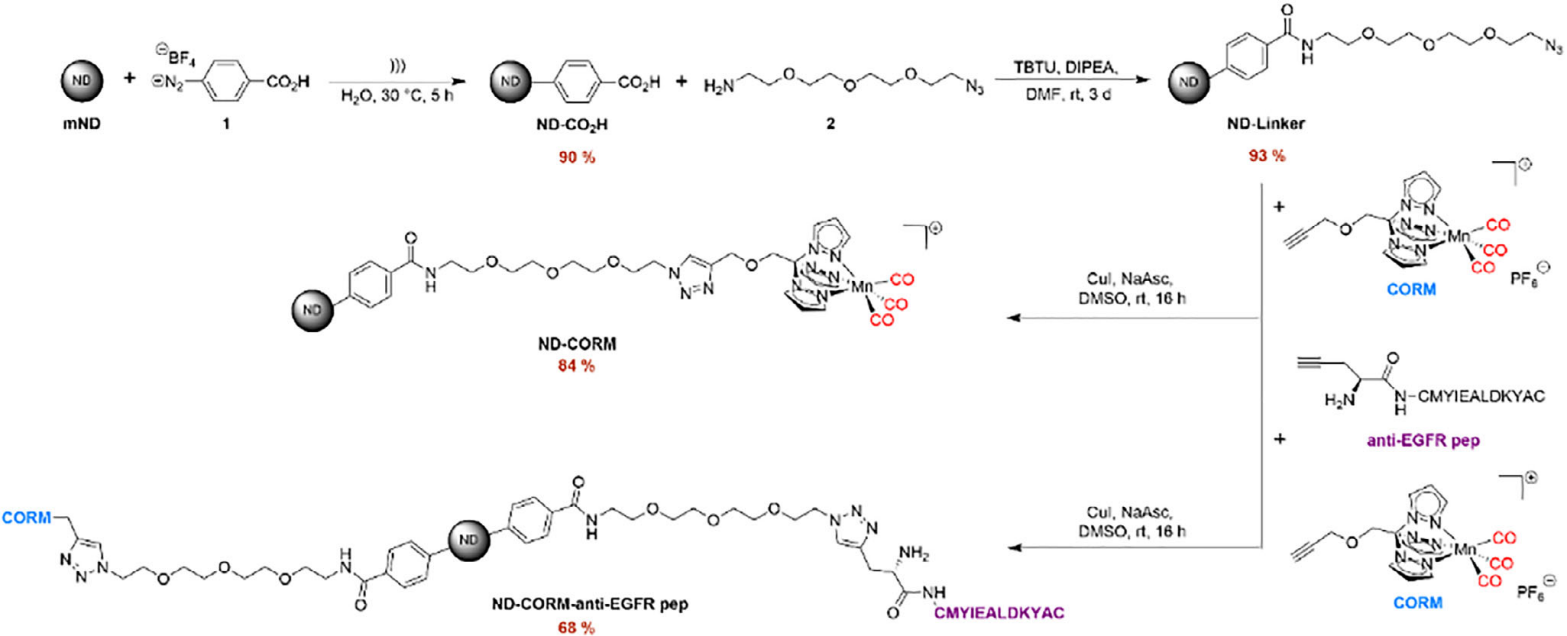
Synthesis of modified nanodiamond (ND) particles starting from functionalization of fully dispersed detonation nanodiamond mND with diazonium salt **1**, yielding benzoic acid‐functionalized **ND‐** **CO**_**2**_ **H**, which was further modified by amide coupling of triethylene glycol derivative **2**. **ND‐Linker** with terminal azide groups was subsequently used for immobilization of alkyne‐modified [Mn(CO)_3_(tpm)]PF_6_ (**CORM**) to obtain **ND‐CORM** or **CORM** combined with EGFR‐targeting peptide (**anti‐EGFR pep**) to yield **ND‐CORM‐anti‐EGFR pep**. The yields of the respective ND conjugates are shown in red.

**FIGURE 1:**
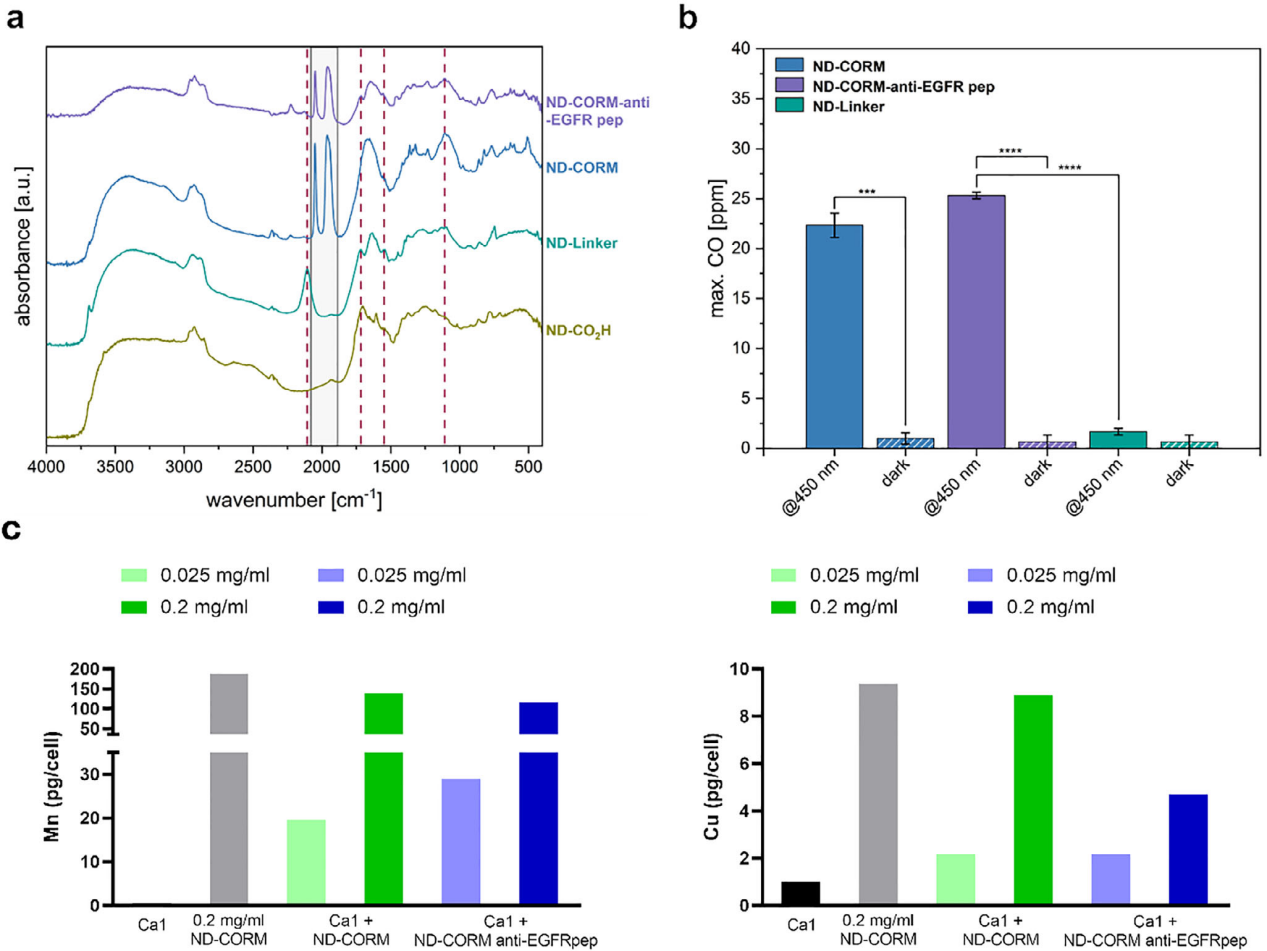
(a) DRIFT spectra of the functionalized NDs and elemental analysis of Mn and Cu. Dashed lines indicate significant signals due to the organic surface functionalization of the respective ND conjugates, the gray box highlights the region showing the spectroscopic signature of the *fac*‐Mn(CO)_3_ moiety at 2050 cm^−1^ and 1960 cm–^1^. (b) Maximum detected CO concentration released by 200 µg of **ND‐CORM** or **ND‐CORM‐anti‐EGFR pep** after 1 h of photoactivation at 450 nm (100% optical power, 10 cm distance to the LED light source) or kept in the dark. **ND‐Linker** was employed as negative control. Error bars show the standard error of the mean of triplicate measurements. ^***^ *p* ≈ 0.0005, ^****^ *p* < 0.0001. (c) Elemental analysis of Mn and Cu in cells treated with different concentrations of **ND‐CORM** or **ND‐CORM anti‐EGFR pep** to verify targeting effect and potential Cu‐associated toxicity, respectively. Ca1 and **ND‐CORM** only were used as negative and positive controls.

Starting from milled detonation nanodiamond (**mND**) (Scheme 1), benzoic acid moieties were grafted onto ND by radical arylation of aromatic diazonium salt **1**, forming stable C─C bonds to the diamond surface. The obtained benzoic acid‐modified particles **ND‐** **CO**_**2**_ **H** displayed a highly negative zeta potential of −32.7 mV (doubly distilled water (dd‐H_2_O), pH 6.8) and form stable dispersions in water (Table 1). In the infrared spectrum of **ND‐** **CO**_**2**_ **H** recorded using diffuse reflectance infrared Fourier transform spectroscopy (DRIFTS) (Figure 1a), the stretching vibration of the aromatic carboxylic acid groups at 1705 cm^−1^ is clearly visible, while the band at 1607 cm^−1^ indicates the C = C stretching vibration of the aromatic ring.

**TABLE 1:** Summary of zeta potential (ζ) and particle size distribution data of all produced ND conjugates.

ND material	zeta potential ζ [mV] (pH)	D_v_(10) [nm]^a^	D_v_(50) [nm]^a^	D_v_(90) [nm]^a^
**ND‐CO**_**2**_ **H**	−32.7 (6.8)	38.4	57.8	101
**ND‐Linker**	+37.6 (5.7)	53.1	81.7	151
**ND‐CORM**	+22.7 (6.5)	55.1	83.7	152
**ND‐CORM‐anti‐EGFR pep**	+12.5 (6.2)	52.0	80.1	152

^a^ Particle size distributions were measured as hydrodynamic diameter in doubly distilled water (dd‐H_2_O) by dynamic light scattering (DLS) and are reported as volume distributions (D_v_(10), D_v_(50), D_v_(90)).

In the next step, azide‐modified triethylene glycol derivative **2** (detailed procedures for the synthesis of organic compounds can be found in the Supplementary Information) was anchored to **ND‐CO**_**2**_ **H** by amide coupling. The reaction progress could be easily monitored by DRIFT spectroscopy, as the intense band of the azide‐stretching vibration appears at 2105 cm^−1^. Additionally, an inversion of the zeta potential from negative values to +37.6 mV (dd‐H_2_O, pH 5.7) of the resulting conjugate **ND‐Linker** confirms successful attachment of the hydrophilic molecule. The respective DRIFT spectrum shows further characteristic bands such as amide I and amide II stretches at 1718 and 1544 cm^−1^, as well as vibrations of the ethylene glycol bridges around 1100 cm^−1^ (Figure 1a). Particle size measurements by dynamic light scattering (DLS) reveal hydrodynamic diameters of less than 152 nm for **ND‐Linker** (Table 1), which underlines the high colloidal stability of the particles primarily arising from hydrogen bonds with the surrounding water molecules. Using an analogous system of functionalized ND particles, we demonstrated significantly enhanced dispersibility and stability, even in protein‐rich media [55].

Owing to its design, **ND‐Linker** enables covalent attachment of **CORM** and **anti‐EGFR pep** via copper(I)‐catalyzed click reaction, affording particles with well‐defined organic surface functionalization and robustness against both hydrolysis and enzymatic degradation. The reaction progress was easily monitored by DRIFT spectroscopy following the decreasing intensity of the azide vibrational band as the azide groups were converted into stable triazole units. Successful attachment of **CORM** to **ND‐Linker** is most clearly evidenced by the appearance of the distinct spectroscopic signature of the *fac*‐Mn(CO)_3_ moiety at 2050 and 1960 cm^−1^ in the DRIFT spectra (Figure 1a) of **ND‐CORM** and **ND‐CORM‐anti‐EGFR pep**. Even after immobilization of the CO‐releasing molecule as well as the EGFR‐targeting peptide consisting of thirteen amino acids, the particle sizes of the obtained ND conjugates remained below 160 nm, which ensures efficient cellular internalization. Furthermore, **ND‐CORM** and **ND‐CORM‐anti‐EGFR pep** exhibit zeta potentials of +22.7 mV (dd‐H_2_O, pH 6.5) and +12.5 mV (dd‐H_2_O, pH 6.2), respectively (Table 1). HRTEM images reveal aggregated nanodiamond clusters with progressively reduced lattice fringe visibility from **ND‐CO**_**2**_ **H** to **ND‐CORM‐anti‐EGFR pep**, indicating successive surface functionalization. While the pristine **ND‐CO**_**2**_ **H** exhibits discernible crystalline domains, the later stages show increased amorphous contrast due to organic coating layers (Figure S25).

Once ND functionalization with **CORM** and the targeting peptide was achieved, we investigated the CO‐release from **ND‐CORM** and **ND‐CORM‐anti‐EGFR pep** upon photoactivation. Although 365 nm is the optimum wavelength for the liberation of CO from **CORM**, light exposure at this wavelength poses limitations for biomedical applications due to its poor tissue penetration and potential phototoxicity associated with UV light. For this reason, we illuminated **ND‐CORM** and **ND‐CORM‐anti‐EGFR pep** dispersed in phosphate buffered saline (PBS) with commercial blue light LEDs at 450 nm and directly measured the released CO by employing a gas sensor. After 1 h of photoactivation of 200 µg of **ND‐CORM** or **ND‐CORM‐anti‐EGFR pep**, a CO concentration of 22.3 ± 1.2 ppm or 25.3 ± 0.3 ppm could be detected, which corresponds to total CO yields of 9.0 ± 0.5 nmol (45 ± 2.5 pmol per µg of **ND‐CORM**) or 10.2 ± 0.1 nmol (51 ± 0.5 pmol per µg of **ND‐CORM‐anti‐EGFR pep**), respectively (Figure 1b and Figure S26a). Furthermore, significant CO release was only recorded upon photoactivation of **ND‐CORM** (*p* ≈ 0.0005) and **ND‐CORM‐anti‐EGFR pep** (*p* < 0.0001) compared to the absence of blue light illumination and is restricted to PhotoCORM‐functionalized ND conjugates as **ND‐Linker** without attached **CORM** did not show significant CO release under illumination when compared to **ND‐CORM‐anti‐EGFR pep** (*p* < 0.0001). These results confirm the photorelease of cytotoxic CO, which can then be used for therapeutic purposes in the field of topically treatable tumors [56, 57, 58].

The CO release profiles of **ND‐CORM** and **ND‐CORM‐anti‐EGFR pep** recorded at an optical power of 100% (corresponds to an irradiance of ∼3.8 mW cm^−2^) and at a distance of 10 cm to the light source exhibit a rapid onset within the first 30 min, followed by a gradually decreasing CO production rate (Figure S26a). To assess the impact of a reduced light intensity on CO release under conditions relevant to biological tissue, where light is scattered and absorbed, the CO production from **ND‐CORM‐anti‐EGFR pep** was monitored at 50% and 10% optical power of the LEDs (Figure S26b). The observed decrease in the respective CO yields suggests a light dose‐dependent CO release, which may limit the therapeutic efficacy in deep‐seated tumors. However, CO release persists even at 10% optical power, indicating that the system remains functionally active under conditions of attenuated light. Given that the released CO may diffuse within the tumor, even low levels of CO could contribute to promoting apoptosis. In addition, the influence of the distance between the light source and the sample on photoactivation efficacy was investigated. When reducing the distance, the CO production rate increased as well as the total amount of CO released by **ND‐CORM‐anti‐EGFR pep** (Figure S26c). These results underscore the therapeutic potential of photoinduced cytotoxicity by PhotoCORM‐functionalized NDs for the treatment of superficial tumors. Moreover, CO release could be also visualized by the decrease in the intensity of the two C≡O vibrations at 2050 and 1960 cm^−1^ in the DRIFT spectra of **ND‐CORM** with illumination time (Figure S26d).

### 2.2 Biocompatibility of Functionalized Nanodiamond Precursors

**FIGURE 2:**
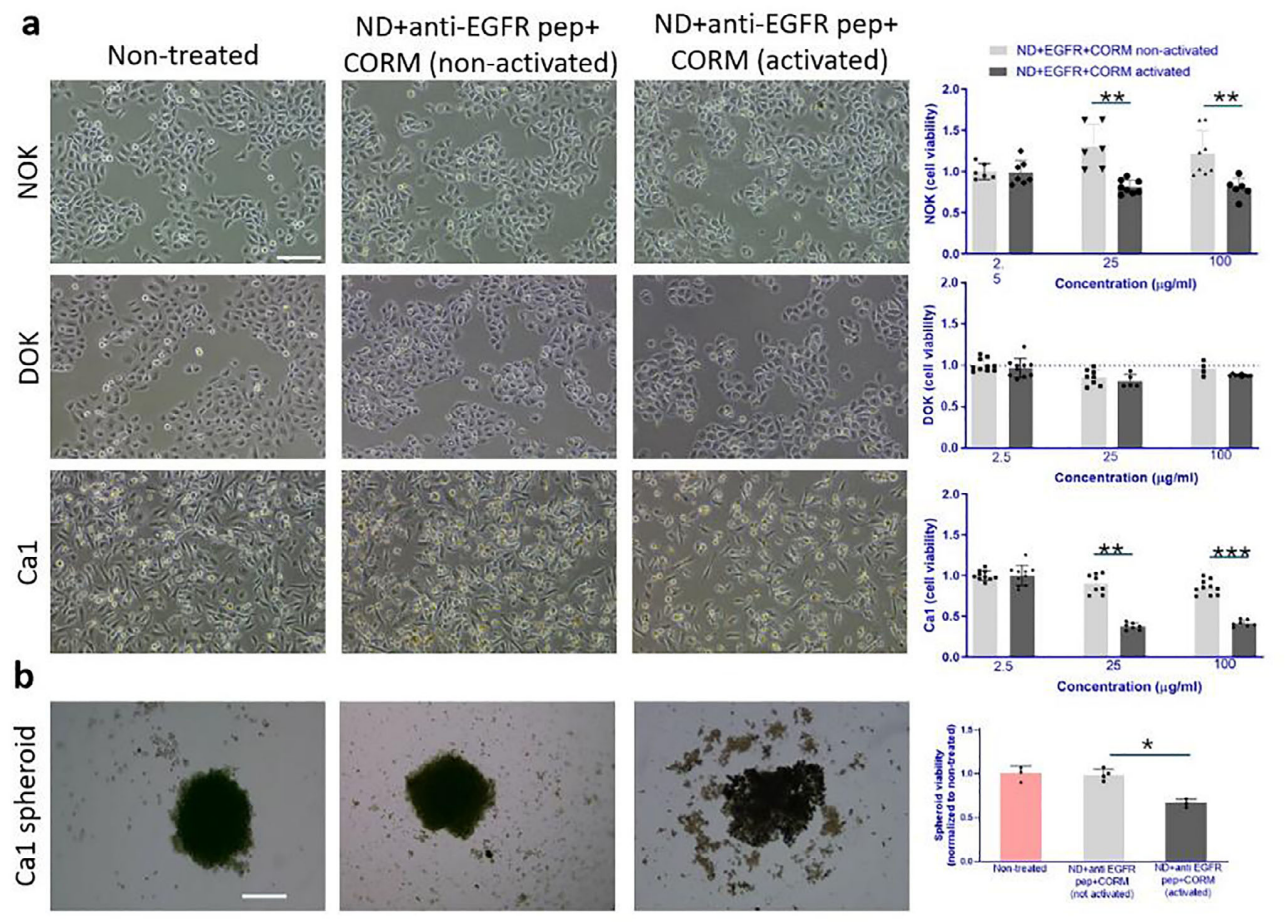
Photoactivation‐induced cytotoxicity of **ND‐CORM‐anti‐EGFR pep** on an i‐CORMn vitro stepwise model of head and neck cancer. (a) Phase contrast images of NOK, DOK, and Ca1 cancer cells following treatment with **ND‐CORM‐anti‐EGFR pep** at 25 µg mL^−1^ with and without photoactivation using 450 nm light. Quantification of cell viability of NOK, DOK, and Ca1 at 2.5, 25, and 100 µg mL^−1^. Scale bar: 100 µm. Data is presented as mean ± SD. ^**^ *p* = 0.007; ^***^ *p* = 0.001. (b) Representative images of Ca1 spheroids when non‐treated, treated but without and with photoactivation. Graphical quantification of viability of 3D Ca1 tumor spheroids. Scale bar: 200 µm. Data is presented as mean ± SD. ^**^ *p* = 0.05.

To verify the targeting effect of the anti‐EGFR peptide, quantitative cellular uptake of manganese was assessed by Inductively Coupled Plasma Mass Spectrometry (ICP‐MS) in a head and neck cancer cell line (Cal). Cells treated with **ND‐CORM‐anti‐EGFR pep** exhibited higher intracellular Mn levels compared to **ND‐CORM** at 0.025 mg mL^−^^1^, confirming enhanced EGFR‐mediated uptake. At a higher concentration (0.2 mg mL^−^^1^), this difference became negligible, indicating a shift toward non‐specific or passive uptake (Figure 1c). In addition, intracellular Cu levels were quantified to evaluate potential Cu‐associated toxicity, revealing lower Cu accumulation in cells treated with **ND‐CORM‐anti‐EGFR pep** even at elevated concentrations (Figure 1c). The biocompatibility of **ND‐CORM‐anti‐EGFR pep** and the precursor **mND** as well as non‐targeting reference material **ND‐CORM** were tested on normal, pre‐malignant, and cancer cell lines to rule out off‐target effects (Figure 2 and Figure S27a,b). Even though copper was present at low levels, all tested ND materials were non‐toxic to normal oral epithelial cells (NOKs), premalignant dysplastic oral keratinocytes (DOKs), and cancer cell line (Ca1) [59] in 2D in vitro cultures at different concentrations without photoactivation. These results indicate a favorable safety profile of all precursors and the absence of adverse biological effects. To further validate the biocompatibility under more physiologically relevant conditions, the NDs were tested on 3D Ca1 tumor spheroids, which better recapitulate in vivo tumor architecture, including cell‐cell interactions and nutrients availability. At 25 µg mL^−1^, the viability of treated Ca1 spheroids was similar in the presence of all tested precursors upon photoactivation and did not change significantly from the untreated controls (Figure S27c).

### 2.3 Controlled CO Release From Functionalized NDs Reduce Viability and Promote Apoptosis in Cancer Cells

Controlled targeting of NDs and subsequent drug release can be achieved through either the use of specific targeting peptides or by modulating the drug release mechanism itself [60, 61]. Unlike many tumor‐homing peptides that exhibit promiscuous binding to normal cells, EGFR‐targeting peptides exploit a receptor‐driven mechanism that is disease‐specific and clinically validated [53, 62, 63]. Furthermore, photoactivated CO‐release enables precise spatiotemporal control of drug release, thereby enhancing specificity and minimizing off‐target effects [64, 65, 66].

To initiate controlled drug release, photoactivation was performed using 450 nm light. As illustrated in Figure 2a, light‐induced CO release resulted in a marked increase in cytotoxicity across all cell types, with the most pronounced effect observed in Ca1 cancer cells. Compared to non‐treated Ca1 controls, cell viability was reduced by 52 ± 7% at 25 µg mL^−1^ (*p* = 0.005) and by 50 ± 6% at 100 µg mL^−1^ (*p* = 0.0004). In addition, reduced viability was also observed in 3D spheroids, where the viability of treated and light‐activated spheroids was reduced significantly by 44 ± 3% (*p* = 0.05). Morphological alterations in spheroids following drug treatment can serve as reliable indicators of therapeutic response [67]. Key changes such as reduced spheroid size and loss of compactness indicate inhibition of cell proliferation or induction of apoptosis [67]. These findings highlight the efficacy of photoactivated CORM‐functionalized ND systems in inducing selective cytotoxicity in EGFR‐overexpressing cancer cells. The significant reduction in viability upon light activation underscores the potential of this platform for spatiotemporally controlled drug release, minimizing damage to surrounding healthy tissue. The dose‐dependent response further supports the tunability of therapeutic intensity, which is critical for optimizing treatment regimens.

**FIGURE 3:**
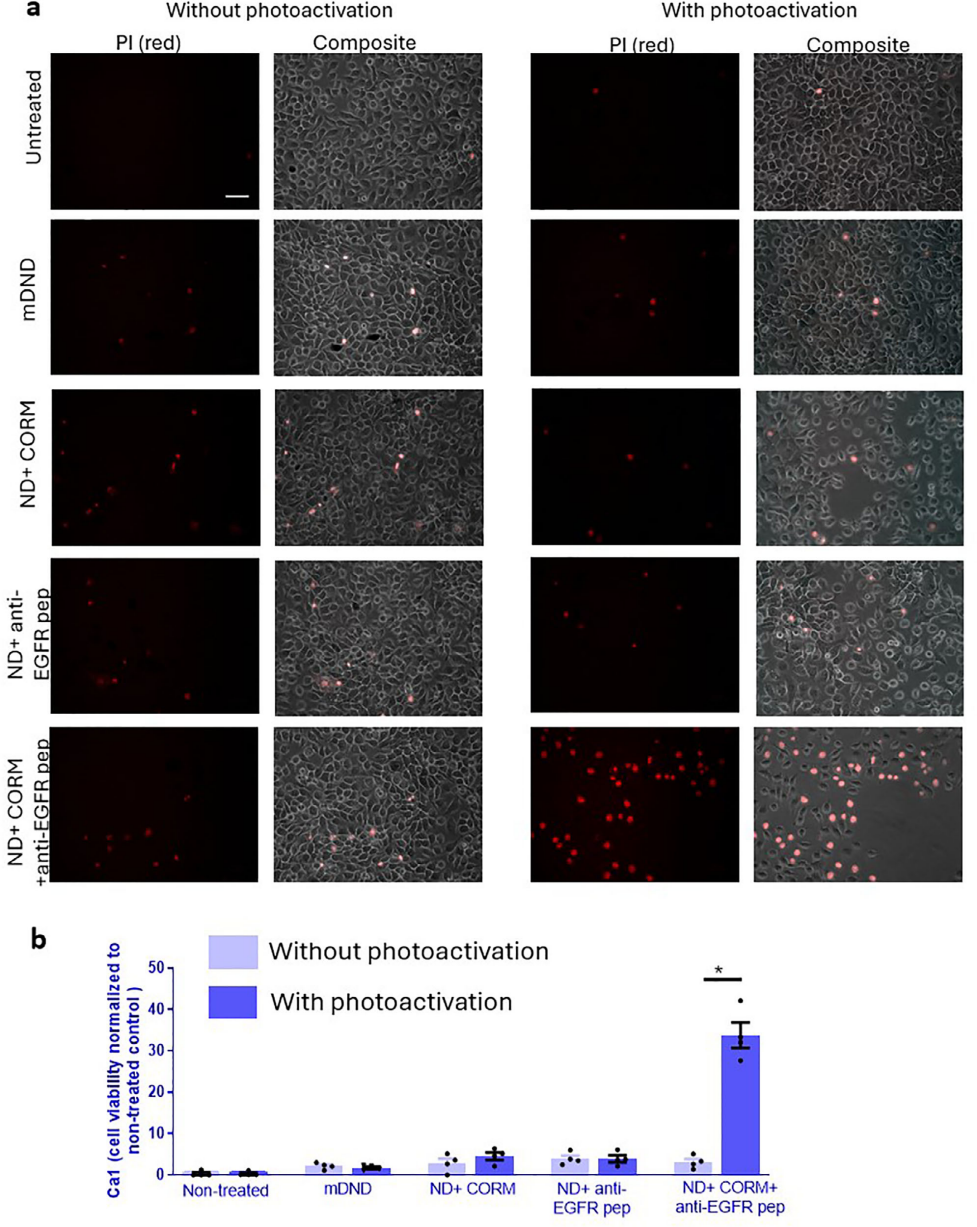
Photoactivation increases the number of late apoptotic/necrotic cells. (a) Representative fluorescence microscopy images of 2D in vitro Ca1 cell cultures (*n* = 4 for each ND) stained with PI to assess membrane integrity without and with photoactivation. Scale bar: 2 µm. (b) Quantification of PI‐positive cells using ImageJ analysis. Data is presented as mean ± SD. ^*^ *p* = 0.02.

To evaluate cell death in the remaining viable population, propidium iodide (PI) staining was performed on 2D in vitro cultures. Without photoactivation, less than 5% of the cancer cells were positive for PI when treated with either the precursors or the **ND‐CORM‐anti‐EGFR pep** as determined by fluorescence microscopy (Figure 3a) and later quantified by ImageJ (Figure 3b). However, upon photoactivation after **ND‐CORM‐anti‐EGFR pep** treatment, 34 ± 5% of the viable cells exhibited PI‐positive staining compared to cancer cells without photoactivation (*p* = 0.02), indicating compromised membrane integrity consistent with late‐stage apoptosis and/or necrosis [68] (Figure 3b). The observed PI positivity upon photoactivation likely reflects CO‐induced mitochondrial dysfunction and oxidative stress. Carbon monoxide has a high affinity for cytochrome c oxidase, which disrupts electron transport, leading to ATP depletion and ROS generation. These events synergistically compromise membrane integrity, consistent with late‐stage apoptosis or necrosis. Thus, PI positivity exclusively in the photoactivated **ND‐CORM‐anti‐EGFR‐pep** treatment supports that CO release, rather than ND uptake alone, is responsible for the cytotoxicity in cancer cells [69].

These findings suggest that while NDs significantly impair cell viability directly via photocytotoxicity, a substantial proportion of the surviving cells undergo cell death. Such late apoptotic/necrotic cells are likely to be cleared by immune cells, suggesting a dual therapeutic effect of the functionalized NDs: direct cytotoxicity and immune‐mediated clearance initiated by an immunogenic type of cancer cell death. Moreover, the results clearly show that CO‐release and subsequent induction of cell death can be controlled by external blue light.

**FIGURE 4:**
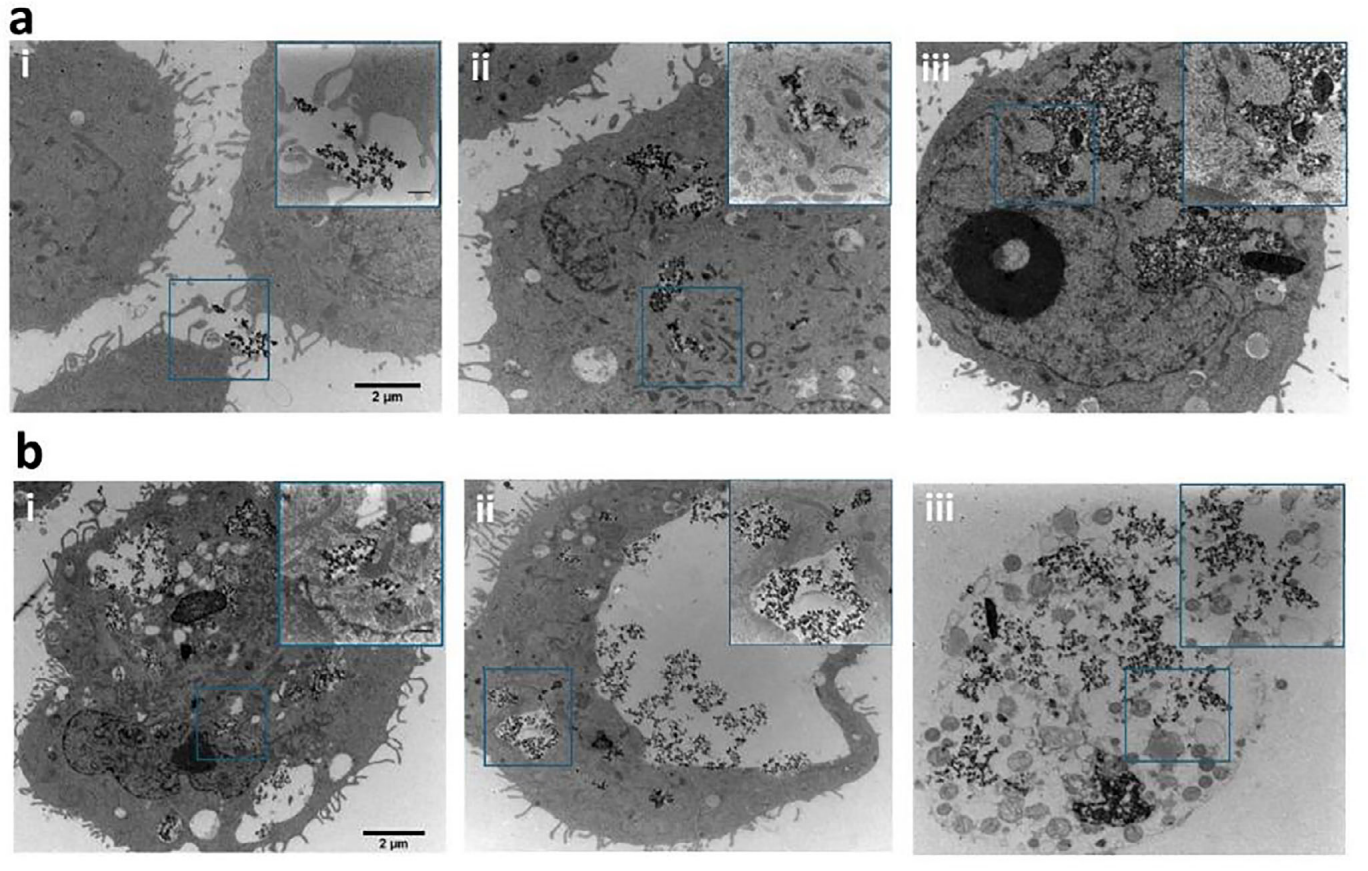
Time‐dependent transmission electron microscopy reveals localization and photoactivation response of **ND‐CORM‐anti‐EGFR pep** in representative cancer cells. (a) **ND‐CORM‐anti‐EGFR pep** localization over time without photoactivation at (i) 30 min, (ii) 6 h, and (iii) 12 h. Inset showing magnified image of the boxed area. Scale bar: 2 µm, inset scale bar: 100 nm. (b) Cellular response following 12 h treatment and photoactivation of **ND‐CORM‐anti‐EGFR pep** after (i) 30 min, (ii) 6 h, and (iii) 12 h, post‐photoactivation. Scale bar: 2 µm, inset scale bar: 100 nm.

Functionalized NDs are efficiently taken up by cells, especially cancer cells, primarily via clathrin‐mediated endocytosis [70]. Their surface chemistry influences the intracellular distribution, with certain functional groups promoting better dispersion and endosomal escape [71]. At earlier time points after treatment, but without photoactivation, **ND‐CORM‐anti‐EGFR pep** particles were predominantly localized at the cell‐cell junction of cancer cells (Figure 4a, panel i), suggesting EGFR‐mediated targeting. Over time, NDs showed abundant accumulation in the cytoplasm, consistent with receptor‐mediated endocytosis (Figure 4a, panel ii and iii). Furthermore, cells were treated with **ND‐CORM‐anti‐EGFR pep** for 12 h followed by photoactivation. As seen in Figure 4b, 30 min after photoactivation, vacuoles could be observed in the cytoplasm suggesting early cellular stress response likely triggered by CO‐release (Figure 4b, panel i). After 6 h, a large vacuole containing NDs could be observed, and at 12 h post photoactivation, complete cell death had occurred (Figure 4b, panel ii and iii). These findings support the therapeutic promise of photoactivatable NDs with spatial control to potentially activate cytotoxic mechanisms involving autophagic or apoptotic pathways at the cellular level.

**FIGURE 5:**
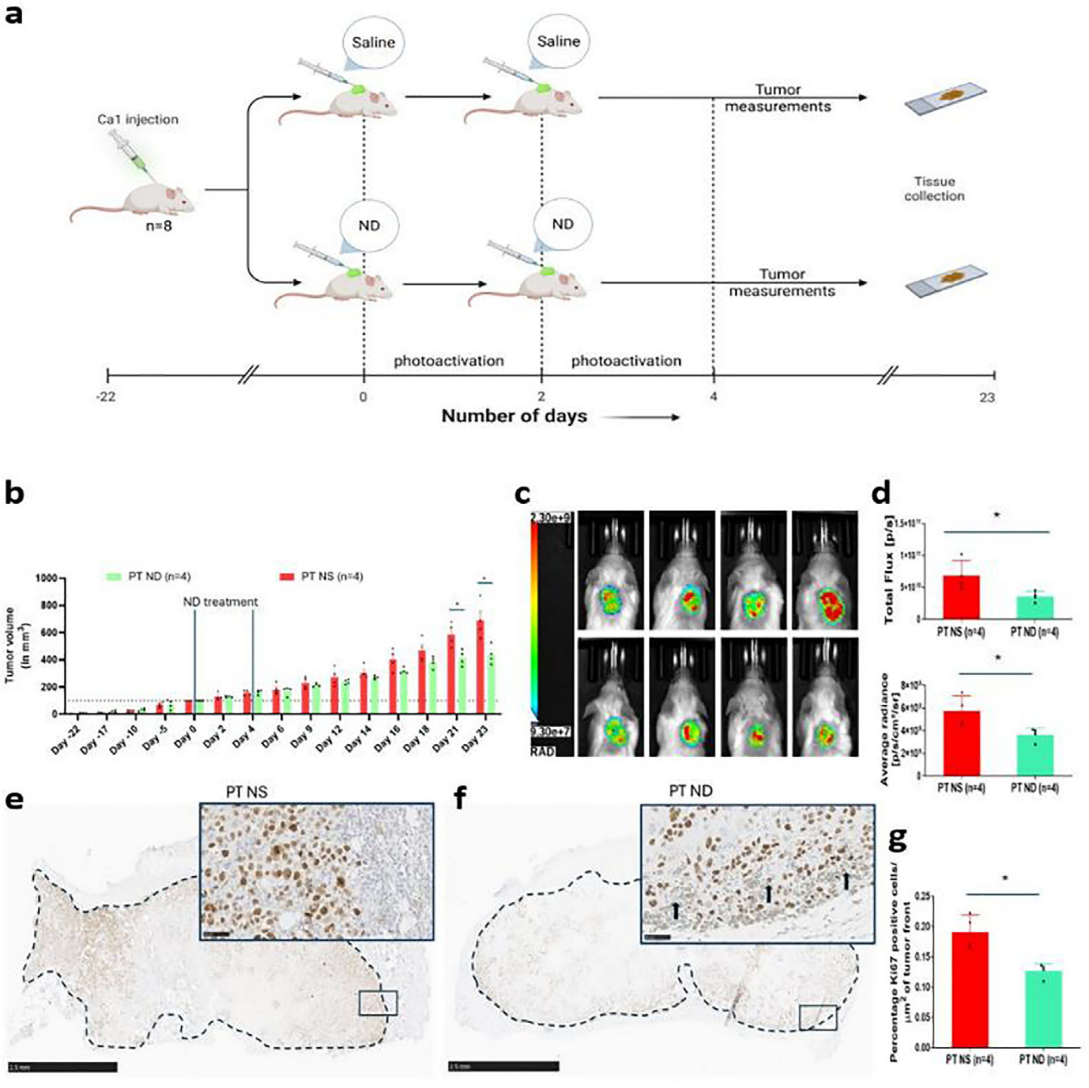
In vivo antitumor efficacy and antiproliferative effects of **ND‐CORM‐anti‐EGFR pep** in Ca1 xenografts. (a) Experimental timeline and treatment regimen. Luciferase‐labelled Ca1 tumors were established in NOD/SCID mice (*n* = 8), randomized to peritumoral injection of **ND‐CORM‐anti‐EGFR** (PT ND, *n* = 4) or saline (PT NS, *n* = 4), and photoactivated twice. (b) Caliper‐measured tumor volumes over the course of treatment. Data is presented as mean ± SD. ^*^ *p* = 0.048 at day 21 and ^*^ *p* = 0.0286 at day 23. (c) Bioluminescence imaging of PT NS (top row) and PT ND (bottom row) groups at day 23. (d) Quantification of total flux (^*^ *p* = 0.05) and average radiance (^*^ *p* = 0.045) at day 23. Representative micrographs of Ki67 immunostaining of the PT NS (e) and PT ND (f) tumors. Scale bar: 2.5 mm. Inset showing a magnified image of the boxed area. Scale bar: 100 µm. Note the presence of the functionalized NDs in the magnified image shown using solid black arrows. (g) Quantification of Ki67 positive cells in the PT NS and PT ND tumor front (^*^ *p* = 0.0286).

To investigate the in vivo effects of **ND‐CORM‐anti‐EGFR pep**, xenografts using luciferase‐labelled Ca1 were first established in NOD/SCID mice and after the tumor volume reached 100 mm^3^, were randomly divided into four groups‐ treated (peritumorally injected ND, PT ND, *n* = 4; intravenously injected IV ND, *n* = 6), non‐treated (peritumorally injected normal saline, PT NS, *n* = 4 and intravenously injected IV NS = 3). The treated groups were given two rounds of ND treatment followed by photoactivation after each round. After three weeks of the last ND treatment, mice were sacrificed, and tumors and lymph nodes were collected (Figure 5a). Tumor measurement using vernier callipers revealed a significant change in volume of treated vs. non‐treated tumors at day 21 in the PT group (382 ± 42 mm^3^ *vs*. 470 ± 85 mm^3^, respectively; *p* = 0.048) and day 23 (415 ± 58 mm^3^ vs. 587 ± 88 mm^3^, respectively; *p* = 0.0268) (Figure 5b). In the IV group, even though a similar trend in the treated vs. non‐treated group was observed at day 21 (249 ± 113 mm^3^ *vs*. 367 ± 17 mm^3^, respectively; *p* = 0.16) and day 23 (296 ± 100 mm^3^ *vs*. 450 ± 39 mm^3^, respectively; *p* = 0.09), statistical significance was not reached (Figure S28). Bioluminescence imaging (BLI) revealed that both total flux and average radiance were significantly decreased in treated vs. non‐treated controls (*p* = 0.05 and *p* = 0.045, respectively) (Figure 5c,d).

These in vivo results demonstrate that **ND‐CORM‐anti‐EGFR pep** significantly suppresses tumor growth. BLI revealed differences in the signal intensity in the treated tumors; hence Ki67 immunostaining was performed to identify proliferating cells in the tumors. Quantification of Ki67 positive cells at the tumor front revealed that the number of proliferating cells was significantly reduced in the treated tumors vs. non‐treated controls (*p* = 0.0286) (Figure 5e–g). A marked decrease in proliferative activity following ND treatment and photoactivation complements the observed cytotoxicity, suggesting the therapy disrupts cell proliferation downstream of EGFR signaling. Together, these findings support the dual action of ND‐mediated CO delivery in both killing tumor cells and halting their in vivo growth.

**FIGURE 6:**
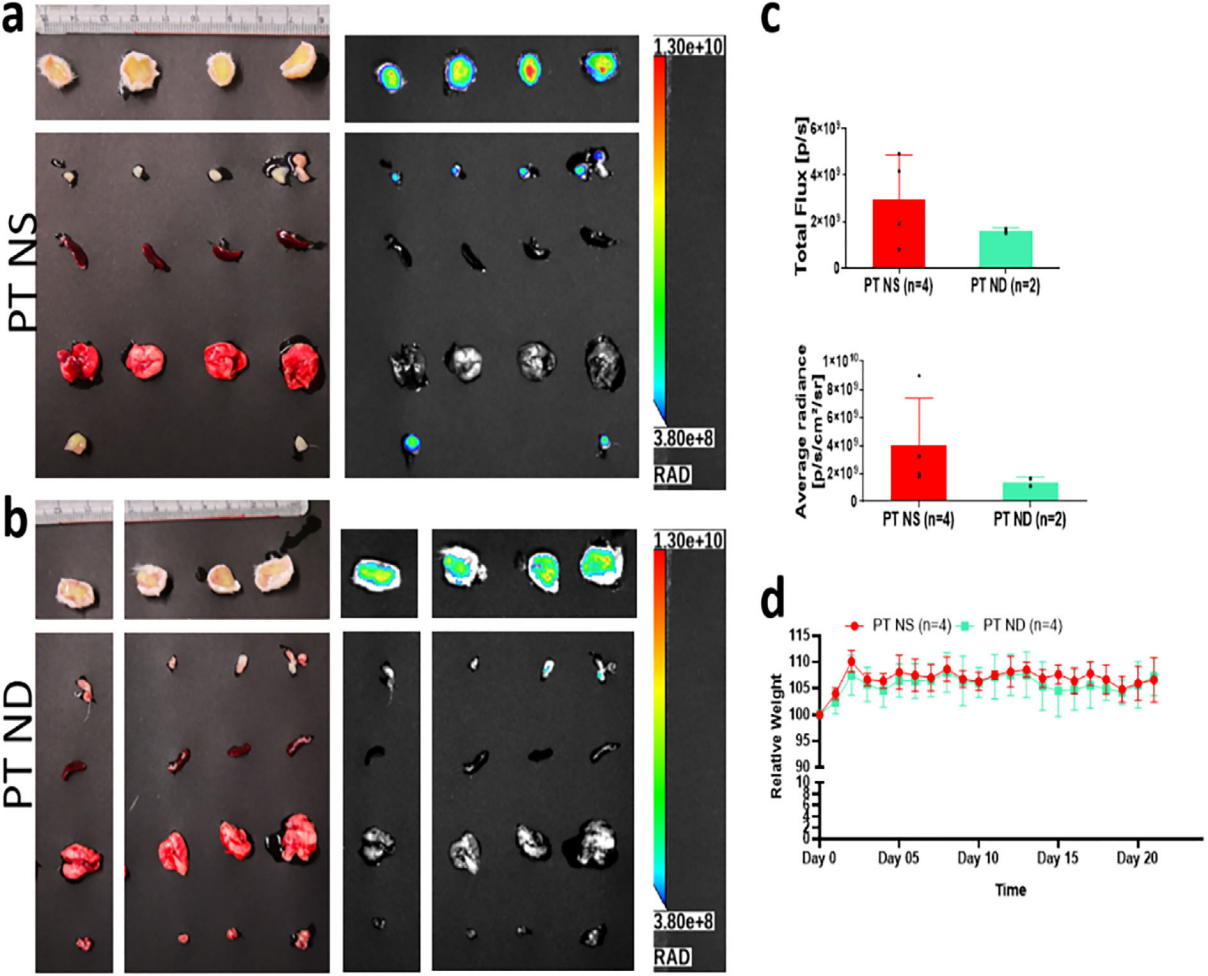
Peritumoral **ND‐CORM‐anti‐EGFR pep** inhibits lymph node metastasis in oral cancer xenografts. BLI of tumors (top), lymph nodes (second), spleen (third), and lungs (fourth) in (a) PT NS and (b) PT ND groups. Note, additional lymph nodes excised from the same animals. (c) Quantification of total flux and average radiance in lymph nodes of both groups. (d) Body weight curves over the treatment period showing no significant differences between groups.

Metastasis remains the leading cause of cancer‐related mortality [72, 73]. In this study, all PT NS (*n* = 4/4, 100%) mice developed clinically apparent lymph node metastasis, whereas in the PT ND group only two mice (*n* = 2/4, 50%) had lymph node metastasis (Figure S29). Of note, several additional lymph node metastases were observed in the PT NS group. *Ex vivo* BLI of harvested lymph nodes confirmed metastases (Figure 6a,b) and HE staining of FFPE sections validated these findings (Figure S30). Although quantification of total flux and average radiance revealed no statistically significant difference between the intensity of lymph node metastases in PT NS and PT ND groups, there was a consistent trend toward lower signal in the PT ND cohort (Figure 6c). No macroscopic metastases were detected in the lung, spleen, kidney, and liver in either group (Figure 6a,b, data not shown for other organs). Mouse body weights remained comparable over the three‐week treatment period in the PT group (Figure 6d), however in the IV ND group, a decrease in body weight in the treated group was observed (Figure S28). Moreover, ultra‐high‐resolution microscopy revealed **ND‐CORM‐anti‐EGFR pep** in the spleen of IV treated mice but not in the PT treated mice suggesting some degree of systemic toxicity (Figure S31). ND accumulation was primarily observed in the spleen rather than the liver, consistent with reticuloendothelial uptake, highlighting the need for detailed long‐term biodistribution studies. Taken together, **ND‐CORM‐anti‐EGFR** peritumoral treatment markedly reduced lymph node metastasis incidence and showed a trend toward lower BLI signals, suggesting inhibition of early tumor dissemination. Further, stable body weights and no observable toxicity in different organs indicate that this approach is systemically well tolerated. EGFR‐targeted, CO‐releasing NDs likely disrupt key metastatic seeding processes, such as cell motility and microenvironment conditioning leading to reduced metastatic dissemination.

Comprehensive immune profiling was not performed and would have limited interpretability due to the use of immunocompromised NOD/SCID mice, which lack functional T‐cell‐mediated adaptive immunity. Moreover, discrepancy between strong in vitro cytotoxicity and continued tumor growth in vivo likely arises from limited light penetration and the absence of adaptive immune clearance in NOD/SCID mice, allowing survival and regrowth of residual tumor cells. Nevertheless, photoactivated ND‐mediated CO release induced pronounced cytotoxicity in vitro and drove a substantial fraction of cells toward late apoptosis/necrosis, a cell death modality often associated with immunogenic clearance [74]. While these findings suggest potential immune‐related contributions, evaluation of nanoparticle‐induced immune activation will require validation in immunocompetent models and remains an important direction for future studies.

## 3 Conclusion

In summary, a highly efficient ND‐based drug delivery tool for a CO‐releasing drug precursor, which can be activated by blue light with high spatial resolution in topically treatable head and neck cancers, was developed. The conjugate consists of fully dispersed and colloidally stable ND particles simultaneously functionalized with a PhotoCORM based on a *fac*‐Mn(CO)_3_ moiety and a small EGFR targeting peptide for the selective addressing of tumor cells. Collectively, our in vitro and in vivo results establish that EGFR‐targeted **ND‐CORM‐anti‐EGFR pep** enables spatiotemporally controlled CO release upon blue‐light activation, driving selective cytotoxicity, cell death, and proliferation blockade in head and neck cancer models, significantly suppressing tumor growth and lymph node metastasis in vivo while maintaining excellent biocompatibility. Further steps including increasing animal cohort size and validating these findings in an immunocompetent mouse model, will shed light on the clinical applicability of these functionalized NDs. This leads the way to novel applications of ND conjugates in the field of stimuli‐responsive, targeted drug delivery.

## 4 Experimental Section

### 4.1 Materials and Methods for Organic Synthesis

Unless otherwise specified, all chemicals used for synthesis were of reagent grade, obtained from commercial suppliers, and used without additional purification. Solvents were distilled prior to use and dried following standard procedures when necessary. Reactions involving air‐ or moisture‐sensitive reagents were carried out in dry glassware under a nitrogen atmosphere. Unless explicitly stated otherwise, all synthetic procedures were performed under ambient conditions. Nuclear magnetic resonance (NMR) spectra (^1^H and ^13^C) were recorded using a Bruker Avance 400 FT‐NMR spectrometer, operating at 400 MHz for ^1^H and 100 MHz for ^13^C. Chemical shifts (δ) are reported in parts per million (ppm), with the residual proton signal of the deuterated solvent used for internal calibration. Fourier‐transform infrared (FT‐IR) spectra were acquired using a Jasco FT‐IR‐410 spectrometer equipped with an attenuated total reflection (ATR) unit suitable for organic compounds. 4‐carboxybenzenediazonium tetrafluoroborate (**1**) was synthesized according to a previously published procedure [75].

### 4.2 Materials and Methods for Nanodiamond Particles Characterization

Pristine detonation nanodiamond powder (acid‐purified, diamond content > 95%) was obtained from Gansu Lingyun Corp. (China) and deagglomerated using an attrition mill with 50 µm zirconia beads, following a previously reported procedure [76]. Milled nanodiamond (**mND**) particles were stored as aqueous dispersions in doubly distilled water (dd‐H_2_O) and subjected to washing steps prior to subsequent functionalization. Washing was performed using a Hettich EBA 21 Typ 1004 benchtop centrifuge with a fixed‐angle rotor (maximum 18 k rpm) for particle isolation, followed by redispersion in fresh solvent using a Bandelin Sonorex Digitec Typ DT52 ultrasonic bath (maximum 80 W, 35 kHz). During the reaction of **mND** with diazonium salt (**1**) sonication was applied using a Branson Sonifier 450 102C (max. 400 W, 19850 – 20050 Hz) with conical 5 mm titanium‐microtip for 5 h at level 2 of 10 in a 60% pulse and 40% pause period. Particle size and zeta potential were determined in dd‐H_2_O (pH 6 – 7) using a Malvern Zetasizer Nano ZS (dynamic light scattering, backscattering mode) equipped with an MPT‐2 autotitrator. Zeta potential measurements were recorded as single points at the intrinsic pH of the sample. Particle size distributions were calculated using the Marquardt algorithm and are reported as volume distributions: D_v_(10), D_v_(50), and D_v_(90). Thermogravimetric analysis (TGA) was conducted on a Perkin‐Elmer STA 6000 under a nitrogen flow of 60 mL·min^−1^. Samples were heated from 30 to 130°C at a rate of 10 K min^−1^, held at 130°C for 60 min, and then further heated to 900°C at 5 K min^−1^. FT‐IR spectra were collected using a Jasco FT‐IR‐410 spectrometer with KBr pellets (∼1 mg sample in 220 mg KBr), which had been dried under vacuum. Additionally, diffuse reflectance infrared Fourier transform spectroscopy (DRIFTS) was performed using a Thermo Fisher Nicolet iS5.

### 4.3 Procedures for Nanodiamond Functionalization and Characterization of Functionalized Nanodiamond

Benzoic acid functionalized ND (**ND‐CO_2_H**) [55]

To 26 mL of **mND** dispersion (26.9 mg mL^−1^, 700 mg nanodiamond) 700 mg of 4‐carboxy‐benzenediazonium tetrafluoroborate **1** (2.97 mmol) were added. The mixture was warmed to 30°C and then dispersed by an ultrasonic horn (output 80 W) for 5 h. The functionalized ND particles were isolated via centrifugation (15 k rpm) and washed repeatedly with 64 mL of dd‐H_2_O (2x), acetone (5x), and acetone/dd‐H_2_O (1:1, v/v) (3x). If necessary, the washing procedure was repeated (until the disappearance of the diazonium band in DRIFTS and a highly negative zeta potential was detected). For further characterization, a small amount of dispersion was dried at 80°C. Zeta potential: −32.7 mV (dd‐H_2_O, intrinsic pH = 6.8). Particle size (DLS): 10% ≤ 38.4 nm, 50% ≤ 57.8 nm, 90% ≤ 101 nm (dd‐H_2_O). Surface loading (TGA): 0.51 mmol g^−1^ = Dm (150 – 450°C) = −4.5%. FT‐IR (DRIFTS): $\tilde{\nu }$ = 3381 (br), 2927 (w, n(C‐H)), 2856 (w, n(C‐H)), 1705 (s, n(C═O)), 1607 (m, n(C═C)), 1412 (w), 1377 (m), 1246 (m), 1177 (w), 1018 (w), 860 (w), 774 (w), 707 (w) cm^−1^.

### 4.4 Azide Linker Functionalized ND (ND‐Linker)

300 mg of benzoic acid functionalized **ND‐CO_2_H** were washed with 30 mL DMF and redispersed in 30 mL of dry DMF under nitrogen atmosphere. Then, 731 mg of 2‐(1H‐benzotriazole‐1‐yl)‐1,1,3,3‐tetramethylamonnium tetrafluoroborate (TBTU, 2.27 mmol), 1.33 mL of *N*,*N*‐diisopropylethylamine (DIPEA, 986 mg, 7.62 mmol), and 200 mg of azide linker **2** (0.91 mmol) were added. The dispersion was stirred at 35°C for 3 d. After the reaction mixture was cooled to rt, the particles were isolated via centrifugation (15 k rpm) and washed repeatedly with 48 mL of DMF (2x), acetone (2x), DMF (1x), DMF/dd‐H_2_O (1:1, v/v) (1x) dd‐H_2_O (3x). For further characterization, a small amount of dispersion was lyophilized. Zeta potential: +37.6 mV (dd‐H_2_O, intrinsic pH = 5.7). Particle size (DLS): 10% ≤ 53.1 nm, 50% ≤ 81.7 nm, 90% ≤ 151 nm (dd‐H_2_O). Surface loading (TGA): 0.18 mmol g^−1^ = Dm (150 – 450°C) = −4.4%. FT‐IR (DRIFTS): $\tilde{\nu }$= 3376 (br), 2937 (w, n(C─H)), 2881 (w, n(C─H)), 2105 (s, n(N_3_)), 1718 (s, n(C═O), amide I), 1636 (s), 1544 (m, n(C─N), amide II), 1449 (w, δ(C─H)), 1374 (w), 1272 (w), 1180 (w), 1126 (w, n(C═O_ether_)), 1100 (w, n(C═O_ether_)), 926 (w), 858 (w), 746 (m) cm^−1^.

### 4.5 CORM‐Functionalized ND (ND‐CORM)

35 mg of **ND‐Linker** were washed with DMF and redispersed in 6 mL degassed DMF. Under N_2_ atmosphere, the ND dispersion was degassed through N_2_ bubbling and simultaneous sonication for 15 min. Then, 13.3 mg cupper(II) sulfate (0.08 mmol) was added, and the dispersion was further degassed for another 15 min. After 33.3 mg of sodium ascorbate (0.16 mmol) were added, the reaction mixture was vigorously stirred for 30 min and then cooled using an ice bath followed by addition of 35 mg of **CORM** (0.063 mmol). The resulting reaction mixture was stirred under N_2_ atmosphere at room temperature in the dark for 6 d. Completion of the reaction was monitored by DRIFTS (disappearance of the azide band at 2100 cm^−1^). The particles were isolated by centrifugation (17 k rpm, 15 min, 4°C) and washed repeatedly with 10 mL of DMF (1x), DMF/dd‐H_2_O (1:1, v/v) (1x), dd‐H_2_O (3x), 0.1 M ethylenediaminetetraacetic acid (EDTA) solution (2x), DMF (1x), acetone (1x) and again with dd‐H_2_O (3x). For further characterization, a small amount of ND dispersion was lyophilized. Zeta potential: +22.7 mV (dd‐H_2_O, intrinsic pH = 6.5). Particle size (DLS): 10% ≤ 55.1 nm, 50% ≤ 83.7 nm, 90% ≤ 152 nm (dd‐H_2_O). FT‐IR (DRIFTS): $\tilde{\nu }$= 3401 (br), 2925 (w, n(C─H)), 2050 (s, n(C≡O)), 1960 (s, n(C≡O)), 1669 (s), 1364 (m), 1321 (m), 1233 (w), 1106 (s, n(C═O_ether_)), 987 (w), 861(w), 821 (w), 768 (w), 670 (w), 632 (w), 605 (w), 508 (w) cm^−1^.

### 4.6 CORM‐EGFR Peptide Functionalized ND (ND‐CORM‐Anti‐EGFR pep)

35 mg of **ND‐Linker** were washed with DMF and redispersed in 6 mL degassed DMF. Under N_2_ atmosphere, the ND dispersion was degassed through N_2_ bubbling and simultaneous sonication for 15 min. Then, 13.3 mg cupper(II) sulfate (0.08 mmol) was added, and the dispersion was further degassed for another 15 min. After 33.3 mg of sodium ascorbate (0.16 mmol) were added, the reaction mixture was vigorously stirred for 30 min and then cooled using an ice bath followed by addition of 35 mg of **CORM** (0.063 mmol) and 90.0 µg (68.4 pmol) of **anti‐EGFR pep**. The resulting reaction mixture was stirred under N_2_ atmosphere at room temperature in the dark for 6 d. Completion of the reaction was monitored by DRIFTS (disappearance of the azide band at 2100 cm^−1^). The particles were isolated by centrifugation (17 k rpm, 15 min, 4°C) and washed repeatedly with 10 mL of DMF (1x), DMF/dd‐H_2_O (1:1, v/v) (1x), dd‐H_2_O (3x), 0.1 M ethylenediaminetetraacetic acid (EDTA) solution (2x), DMF (1x), acetone (1x) and again with dd‐H_2_O (3x). For further characterization, a small amount of ND dispersion was lyophilized. Zeta potential: +12.5 mV (dd‐H_2_O, intrinsic pH = 6.2). Particle size (DLS): 10% ≤ 52.0 nm, 50% ≤ 80.1 nm, 90% ≤ 152 nm (dd‐H_2_O). FT‐IR (DRIFTS): $\tilde{\nu }$= 3396 (br), 2954 (w, n(C─H)), 2925 (m, n(C─H)), 2867 (w, n(C─H)), 2225 (w), 2126 (w), 2049 (s, n(C≡O)), 1959 (s, n(C≡O)), 1715 (m, n(C═O), amide I), 1645 (s), 1556 (w, n(C─N), amide II), 1458 (w, δ(C─H)), 1377 (w), 1339 (w), 1325 (w), 1234 (m), 1106 (m, n(C═O_ether_)), 975 (w), 860 (w), 767 (m), 633 (w), 603 (w), 530 (w) cm^−1^.

### 4.7 Carbon Monoxide‐Release Studies

The release of CO from PhotoCORM functionalized ND particles was studied quantitatively by dispersing 200 µg mL^−1^ of **ND‐CORM**, **ND‐CORM‐anti‐EGFR pep** or **ND‐Linker** in 1 mL of 0.01 M PBS and placing the respective ND dispersion into a small flask equipped with a Dräger XXS CO LC sensor at the top (volume headspace: 10 mL), which was connected to a Dräger X‐am 5000. The setup was sealed and either illuminated with blue light LEDs purchased from Sahlmann Photochemical Solutions with l_max_ = 450 nm (∼3.8 mW cm^−2^ ≙ 100% optical power) at varying distances and optical powers or kept in the dark for 1 h. The CO concentration was directly determined with an instrument‐specific measurement error of ± 1 ppm. Every experiment was conducted in triplicates and data are presented as average ± standard error over mean. Qualitative analysis of the PhotoCORM functionalized ND particles during photoactivation was performed by IR spectroscopy. In brief, 1.5 mg of lyophilized **ND‐CORM** were placed in a quartz cuvette (*d* = 1.0 cm) and redispersed in 1.5 mL of dd‐H_2_O. The ND dispersion was illuminated with the same blue light LEDs positioned perpendicular to the cuvette at a distance of 15 cm. A sample for analysis by DRIFTS was taken from the dispersion before irradiation as well as after 10 and 20 min of irradiation, respectively. The photoactivation was followed by a decrease in the intensity of the two characteristic carbonyl stretching bands at 2050 and 1960 cm^−1^.

### 4.8 Cell Culture, Proliferation Assay and Transmission Electron Microscopy (TEM)

Primary human normal oral keratinocytes (NOKs) were isolated from biopsy samples of normal human oral mucosa as described elsewhere [77]. Dysplastic oral keratinocytes (DOK cell line, accession no. 94122104) were obtained from the European Cell Culture Bank (ECACC) [77]. The Ca1 head and neck cancer cell line was obtained from Dr Ian Mackenzie's lab [78]. Routine maintenance of NOKs, DOKs, and Ca1 is described elsewhere [77, 78]. Alamar blue assay was used to explore the effect of different precursors and **ND‐CORM‐antiEGFRpep** on NOK, DOK, and Ca1 cell proliferation. In brief, the cells were seeded into 96‐well plates at a density of 5.0 × 10^3^ cells per well and allowed to adhere for 24 h in their growth media. After the cells adhered, the cells were washed with 1X PBS and incubated with serum‐free medium containing different concentrations of NDs for 24 h. After 24 h, the cells were washed again with 1X PBS to ensure only the internalized NDs remained. The cells were then irradiated with 450 nm light for photoactivation for 30 min from 10 cm height. Further, the cells were allowed to grow for 24 h, and then viability was evaluated by a standard Alamar blue assay. To analyze the internalization of different NDs using TEM, the cells were seeded in a 6‐well plate at a similar density and followed the same protocol as proliferation assay. The detailed protocol is mentioned elsewhere [54].

### 4.9 Inductively Coupled Plasma Mass Spectrometry (ICP‐MS)

The concentration of manganese and copper in cells was determined by Inductively Coupled Plasma Mass Spectrometry (ICP‐MS). The instrument (Element XR HR‐ICP‐MS, Thermo Fisher Scientific, Bremen, Germany) is equipped with a high resolution, magnetic sector field. The cells were incubated with different concentrations of NDs as previously described for TEM analysis. Before ICP‐MS analysis, an aliquot 1 mL containing either cells or only NDs was acid digested by adding 2 mL 60% ultrapure HNO_3_ and 1 mL 30% H_2_O_2_ (Merck, Darmstadt, Germany) in closed vessels in a microwave‐assisted system (Milestone Ethos UP, Sorisole, Italy). After digestion, the 1 mL aliquotes were diluted 100x times with doubly distilled deionized water (MilliQ, USA). Calibration was performed by standard addition using 0.2, 0.5, 2, and 5 µg L^−1^ calibration solutions prepared from a 10 µg mL^−1^ multi‐element stock standard solution (Spectrascan, Lot Number C2‐MEB294066, Teknolab AB, Kungsbacka, Sweden). An internal standard of indium was injected automatically to all the samples to monitor and correct for any instrumental changes. The samples were analyzed using the built‐in software ELEMENT 2/XR and the results are presented as pg/150,000 cells.

### 4.10 Cytoviva

Ultrahigh‐resolution imaging (URI) (CytoViva, Auburn, USA) was used to assess the presence of **ND‐CORM‐anti‐EGFR pep** in different mice organs. CytoViva is a high contrast optical dark‐field system which gives improved contrast and signal‐to‐noise ratio [54, 79]. Nanodiamonds and other light scattering objects present as bright features on a dark background.

### 4.11 3D Cell Culture

To make 3D spheroids of Ca1 cancer cells, 10,000 cells were seeded in a polyHEMA (Sigma) coated 96‐well plate and allowed to form a uniform spheroid. After 48 h, the spheroids were washed with 1x PBS and treated with different NDs in serum‐free medium. After 24 h, the spheroids were washed again and irradiated with 450 nm light. The viability of spheroids was measured with Alamar blue.

### 4.12 Propidium Iodide (PI) Staining

PI uptake versus exclusion was used to assess cell death associated with loss of plasma cell impermeability. Plasma membranes become permeable to PI typically during late apoptosis and necrosis, thus PI positivity is used as a reliable indicator of these cell death types. PI staining was measured by fluorescence microscopy (Axio Imager Z2, Carl Zeiss, DE).

### 4.13 Statistical Analysis

Statistical analyses were performed using the Prism 10.0 software (GraphPad Software, San Diego, CA, USA). Data is presented as means ± SEM throughout the manuscript, unless specified otherwise. All the quantitative data were analyzed using a two‐way Student's T test with Mann‐Whitney tests. *p* < 0.05 was considered statistically significant.

### 4.14 In Vivo Experiments

All animal experiments were approved by the local ethical board (FOTS ID: 202022627) and carried out at the Animal Research Facility, University of Bergen, Norway. Eight six to eight‐weeks‐old NOD SCID IL2R gamma chain deficient mice (NSG) weighing between 25 g – 30 g were used for this study. One million luciferase‐transfected Ca1 cells resuspended in 100 µL of PBS were injected subcutaneously in the back of the mice (*n* = 8). After the tumors had developed and reached 100 mm^3^, the mice were randomly divided into two groups: one group received 200 µg mL^−1^ of **ND‐CORM‐anti‐EGFR pep** peritumorally (PT ND, *n* = 4), and the other group received normal saline peritumorally (PT NS, *n* = 4). 24 h after PT injection, both groups received irradiation with 450 nm light. Both groups received one more round of PT injections and irradiation. Following this, tumor volumes and weight were monitored every second day until mice reached the planned endpoint or euthanized due to ethical reasons. Tumor volume was calculated as: volume, V (in mm^3^) = (L×W^2^)/2, where L is tumor length, and W is tumor width. In addition, the mice were subjected to in vivo bioluminescent imaging (BLI) once every week. In addition. Photon emissions from the luciferase expressing Ca1 cells were measured using the IVIS Lumina (IVIS Lumina XRMS In vivo Imaging System, PerkinElmer) with Living Image software (version 4.3.1, PerkinElmer), which was used to estimate the level of photon emission per mice. At the endpoint, tumors, lungs, liver, spleen, and kidneys were harvested for histological analysis and immunohistochemistry (IHC).

### 4.15 Histology and IHC

All the harvested tissues were fixed in 10% buffered formalin and embedded in paraffin. 3 – 5 µm thick sections were cut from the formalin‐fixed paraffin embedded (FFPE) blocks and stained with haematoxylin and eosin (H&E) and immunostained with Ki67 to assess proliferation. The detailed protocol is described elsewhere [80].

## Author Contributions

H.N.D. experiments, data analysis, writing first draft, manuscript editing E.M. experiments, data analysis, writing first draft, manuscript editing J.P. experiments, data analysis, writing first draft, manuscript editing H.P. in vivo experiments, data analysis L.L.S. in vitro experiments, data analysis P.J.H experiments, data analysis, manuscript editing R.D. in vitro experiments, data analysis P.R. experiments, data analysis C.A.M. supervision, resources U.S. supervision, resources, manuscript editing L.B. supervision, resources D.E.C. Conceptualization, resources, supervision, manuscript editing A.K. Conceptualization, resources, supervision, writing first draft, manuscript editing.

## Funding

E.M. and A.K. gratefully acknowledge the support by the Federal Ministry of Research, Technology and Space BMFTR (Cluster4Future QSENS, AZ: 03ZU1110FE) and the Carl Zeiss Foundation CZS Center for Quantum Photonics for a QPhoton project. H.N.D acknowledges financial support by UiB‐Idè 2022–23 (project number 103015), University of Bergen, Norway and Norwegian Research Council's Qualification – Research Commercialisation from Publicly Funded Research Program (project number 349789), Norway. D.E.C. acknowledges financial support from West Norway Health Authority (grant nr F‐13105/2024) and The Research Council of Norway through its Center of Excellence funding scheme (Grant No. 22325).

## Conflicts of Interest

The author declares no conflicts of interest.

## Supporting information




**Supporting File**: smll74355‐sup‐0001‐SuppMat.pdf.

## Data Availability

Data for this study are available from the corresponding authors upon reasonable request.
