# Supplementary material for: Multifunctional Nanodiamond Conjugate With a Tumor‐Specific EGFR‐Targeting Peptide and Photoactivated CO Release for Improved Therapeutic Efficacy in Head and Neck Cancers
Source: Small. 2026 Jul 1;22(47):e74355. doi: 10.1002/smll.74355 (PMC13495914; doi:10.1002/smll.74355)
Supplement: Supplementary file 1 [file SMLL-22-e74355-s001.pdf]

Supporting Information

Multifunctional nanodiamond conjugate with a tumor-specific EGFR-targeting peptide and photoactivated CO release for improved therapeutic efficacy in head and neck cancers

Harsh N. Dongre[#], Elisabeth Mayerhoefer[#], Julia Puck, Himalaya Parajuli, Lorena Larios Salazar, Paul Johan Høl, Ridhima Das, Patrick Roth, Christian A. Moen, Ulrich Schatzschneider, Line Bjørge, Daniela E. Costea[*], Anke Krueger[*]

Email: anke.krueger@oc.uni-stuttgart.de; daniela.costea@uib.no

Table of contents:

1. Synthesis of organic compounds

2. Synthesis and characterization of $[Mn(CO)_3(tpm^{CH2OCH2C\equiv CH})]PF_6$ (CORM)

3. Characterization of functionalized NDs

4. Additional results from *in vitro* and *in vivo* studies

5. References

1. Synthesis of organic compounds

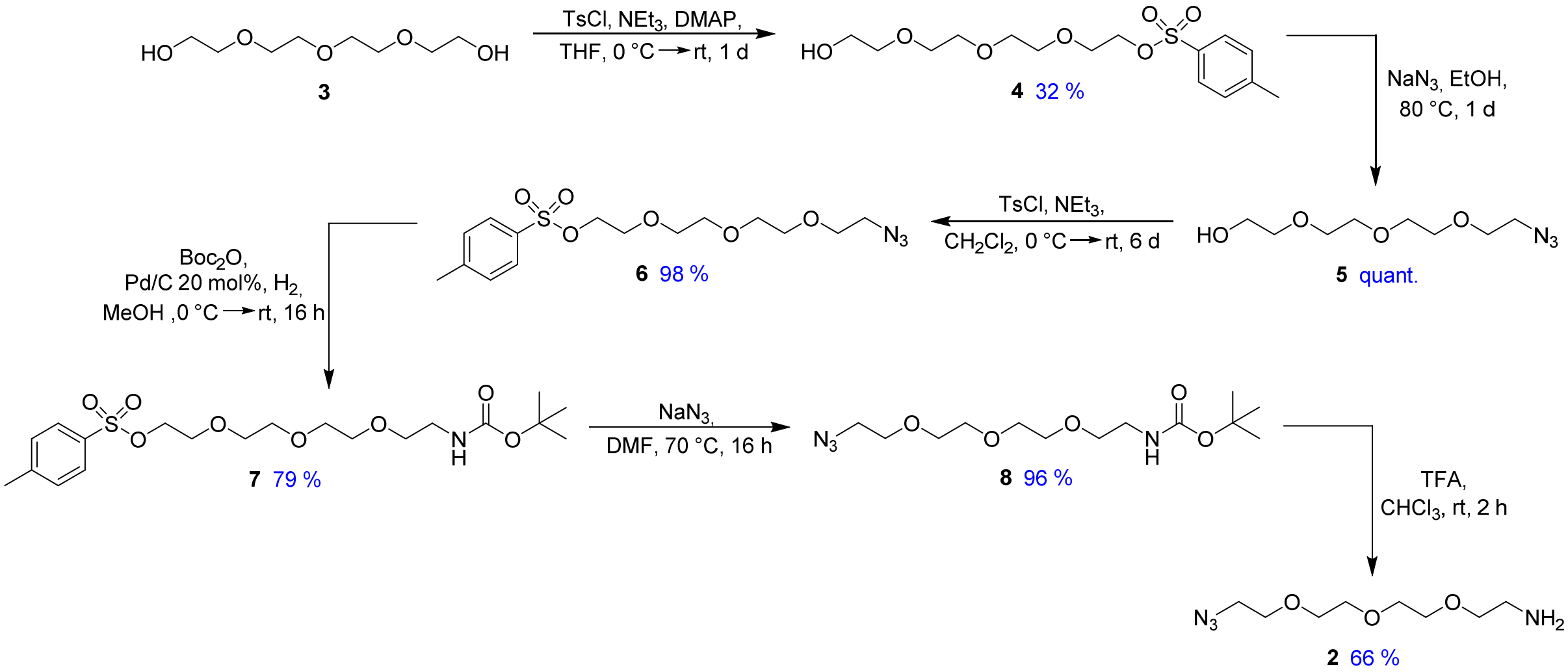

Scheme I. Synthesis of azide-modified triethylene glycol **2** for ND functionalization.

2-[2-[2-(2-Hydroxyethoxy)ethoxy]ethoxy]ethyl 4-methylbenzenesulfonate (**4**)[1]

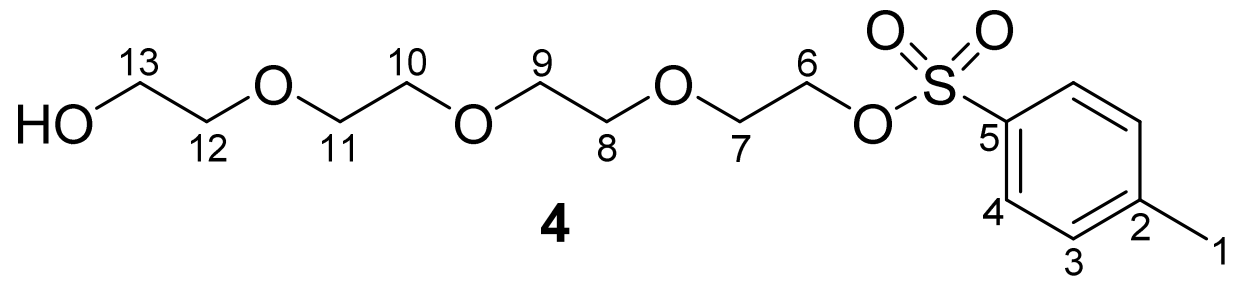

20.0 mL of tetraethylene glycol **3** (22.4 g, 115 mmol, 1.00 eq), 24.0 mL of triethylamine (17.5 g, 173 mmol, 1.50 eq) and 2.82 g of 4-dimethylaminophenol (4-DMAP, 23.1 mmol, 0.20 eq) were dissolved in 100 mL of tetrahydrofuran and subsequently cooled to 0 °C. Then, 24.2 g of tosyl chloride (127 mmol, 1.10 eq) was dissolved in 50 mL of tetrahydrofuran and added dropwise to the reaction mixture. After stirring at room temperature for 1 d, the solvent was removed from the colourless suspension *in vacuo*. The residue was dispersed in 150 mL of hydrochloric acid (2 N) and the resulting aqueous phase was extracted with dichloromethane (4x, 100 mL each). The combined organic layers were dried over $MgSO_4$ and filtered. After removing the solvent *in vacuo*, the crude product was purified by column chromatography (silica, cyclohexane/ethyl acetate, 1:1 → 0:1, v/v). The product **4** was obtained as a colourless oil.

Yield: 12.9 g (37.1 mmol, 32 %). **R_f** 0.30 (cyclohexane/ethyl acetate, 1:2). **^{1}H-NMR** (400 MHz, $CDCl_3$): δ = 7.80 (m, 2H, H-4), 7.34 (m, 2H, H-3), 4.17 (m, 2H, H-6), 3.73 – 3.59 (m, 16H, H-7–13), 2.45 (s, 3H, H-1) ppm. **^{13}C-NMR** (100 MHz, $CDCl_3$): δ = 144.9 (C_q, C-5), 132.9 (C_q, C-2), 129.9 (C_t, C-4), 128.0 (C_t, C-3), 72.5, 70.7, 70.6, 70.4, 69.3, 68.7 (C_s, C-7–12),

61.7 (C_s, C-13), 21.7 (C_p, C-1) ppm. **FT-IR** (ATR): $\tilde{\nu}$ = 3456 (br, ν(O-H), 2872 (w, ν(C-H)), 1598 (w, ν(C=C_{arom})), 1454 (w, δ(C-H)), 1352 (s, ν(S=O)), 1292 (w), 1248 (w), 1174 (vs, ν(C=O_{ether})), 1096 (s, ν(C=O_{ether})), 1013 (m), 917 (vs), 814 (m, δ(C-H_{arom})), 773 (m), 661 (vs) cm^{-1}.

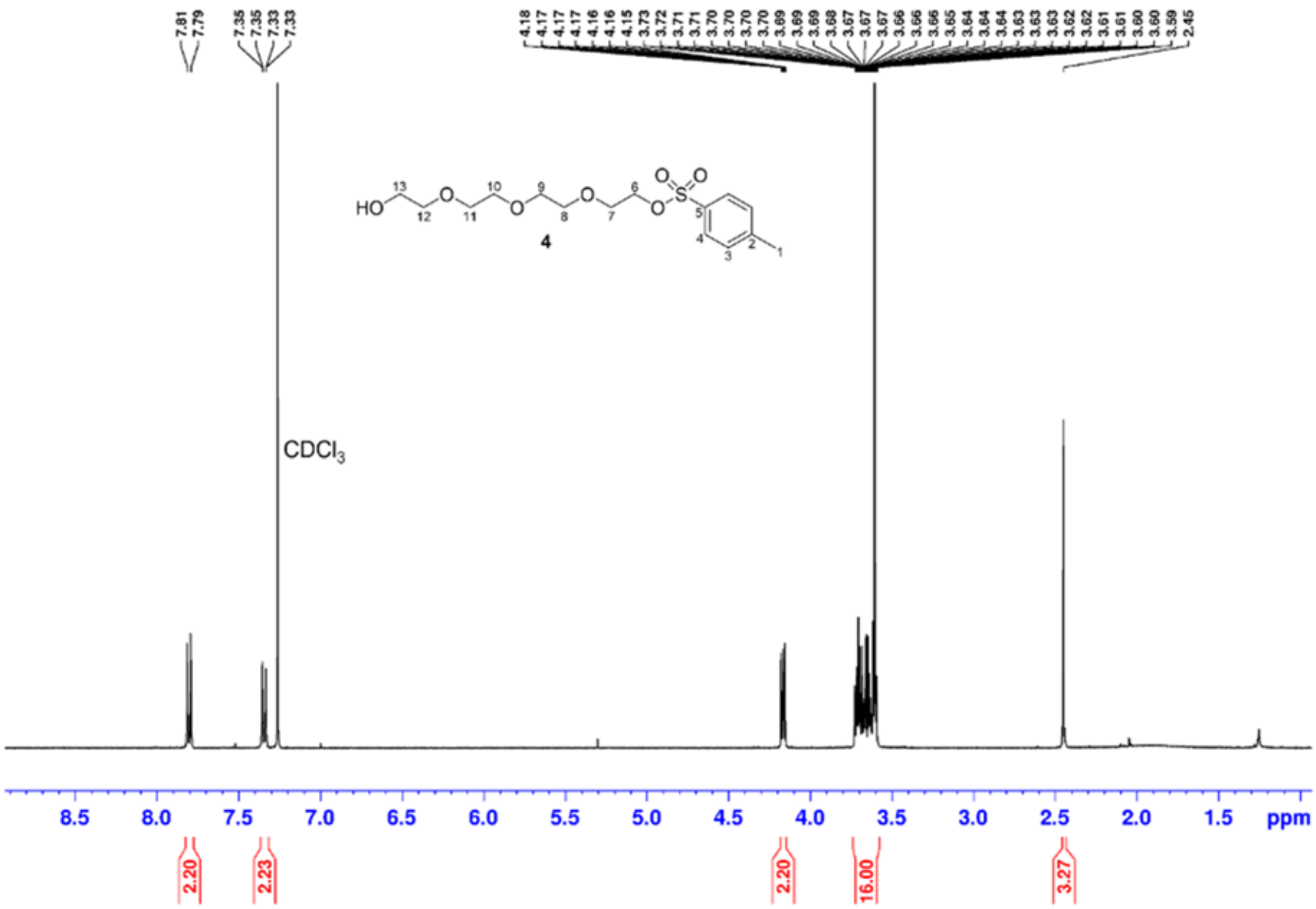

Figure S1. ^{1}H-NMR spectrum (400 MHz, $CDCl_3$) of compound **4**.

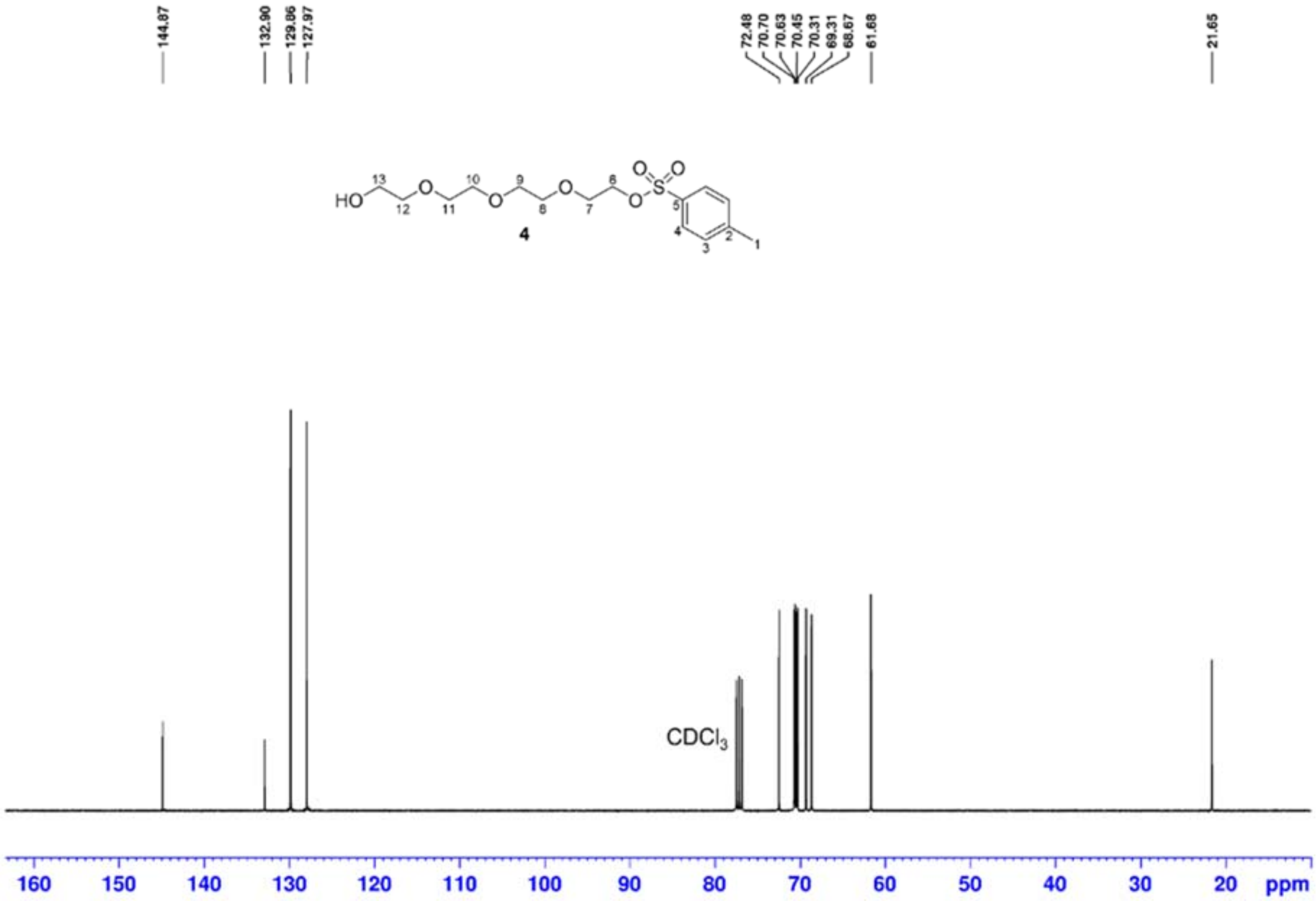

Figure S2. ^{13}C-NMR spectrum (100 MHz, $CDCl_3$) of compound **4**.

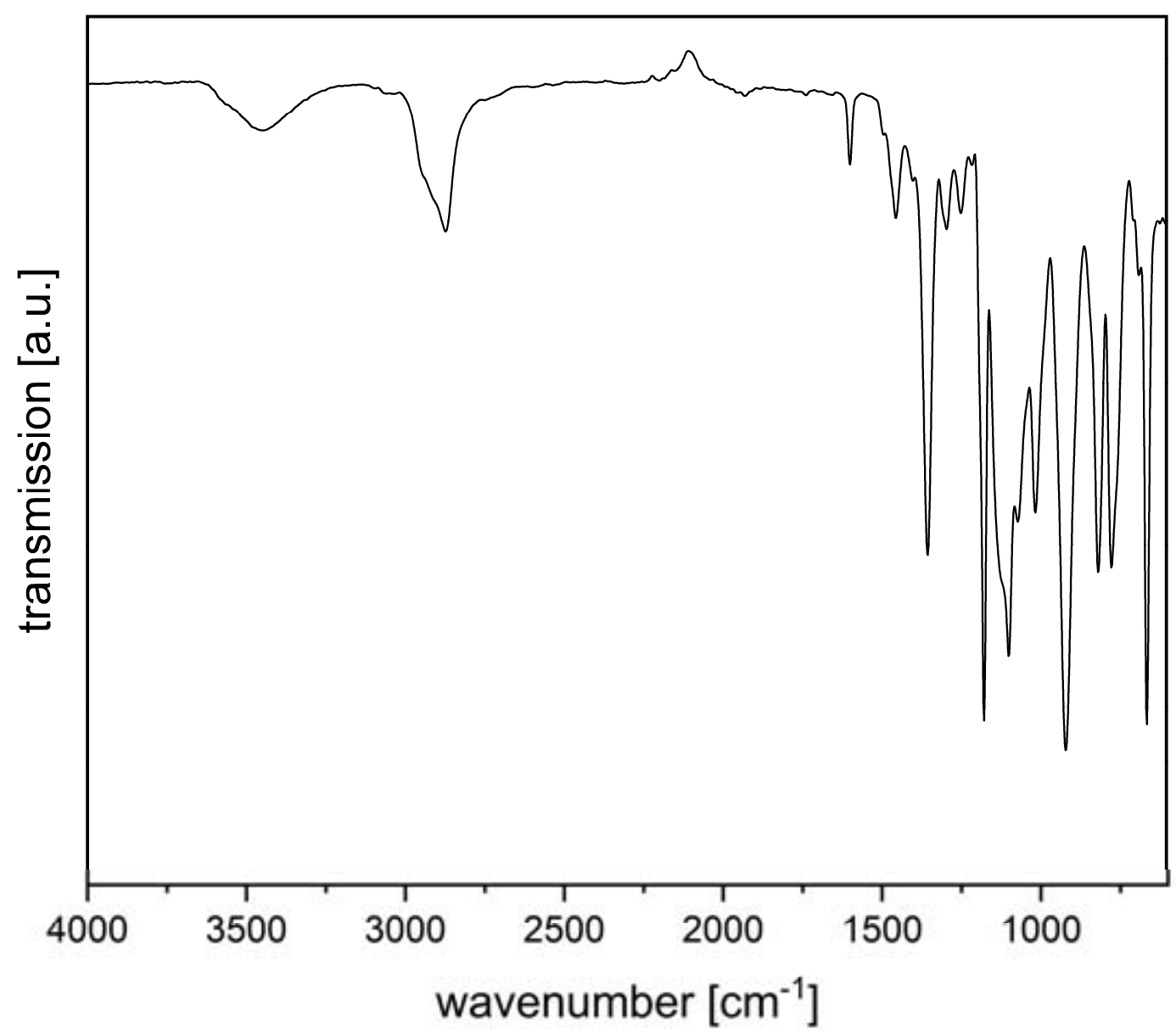

Figure S3. FT-IR (ATR) spectrum of compound **4**.

2-[2-[2-(2-Azidoethoxy)ethoxy]ethoxy]ethanol (**5**)[1]

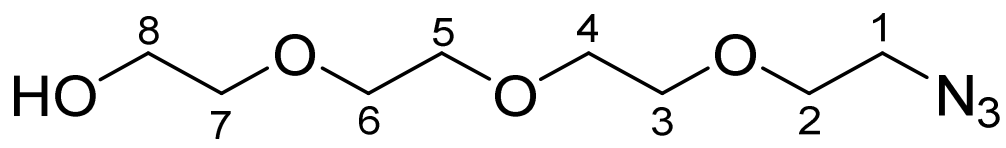

5

5.00 g of tetraethylene glycol monotosylate **4** (14.3 mmol, 1.00 eq) were dissolved in 120 mL of ethanol. After addition of 7.43 g of sodium azide (114 mmol, 8.00 eq), the reaction mixture was stirred overnight at 80 °C. Upon reaction completion, 100 mL of sodium chloride solution were added, and the solvent was subsequently removed *in vacuo*. The aqueous layer was extracted with chloroform (4x, 100 mL each) and the obtained organic layer was dried over Na_2SO_4. After filtration, the solvent was removed *in vacuo* and the product **5** was obtained as pale-yellow oil without further purification.

Yield: 3.14 g (14.3 mmol, 100 %). $\mathbf{R_f}$ 0.13 (cyclohexane/ethyl acetate, 1:2). ^{1}**H-NMR** (400 MHz, $CDCl_3$): δ = 3.74 – 3.72 (m, 2H, H-8), 3.69 – 3.67 (m, 10H, H-3 – 7), 3.63 – 3.61 (m, 2H, H-2), 3.40 (t, $^3J_{1,2}$ = 5.10 Hz, 2H, H-1), 2.17 (br, 1H, O*H*) ppm. ^{13}**C-NMR** (100 MHz, $CDCl_3$): δ = 72.5, 70.7, 70.6, 70.6, 70.3, 70.0 (C_s, C-2–7), 61.7 (C_s, C-8), 50.6 (C_s, C-1) ppm. **FT-IR** (ATR): ṽ = 3431 (br, ν(O-H), 2870 (w, ν(C-H)), 2098 (s, ν(N_3)), 1452 (w, δ(C-H)), 1348 (w), 1286 (m), 1103 (vs, ν($C=O_{ether}$)), 1068 (s, ν($C=O_{ether}$)), 933 (m), 885 (w), 833 (w, δ($C-H_{arom}$)) cm^{-1}.

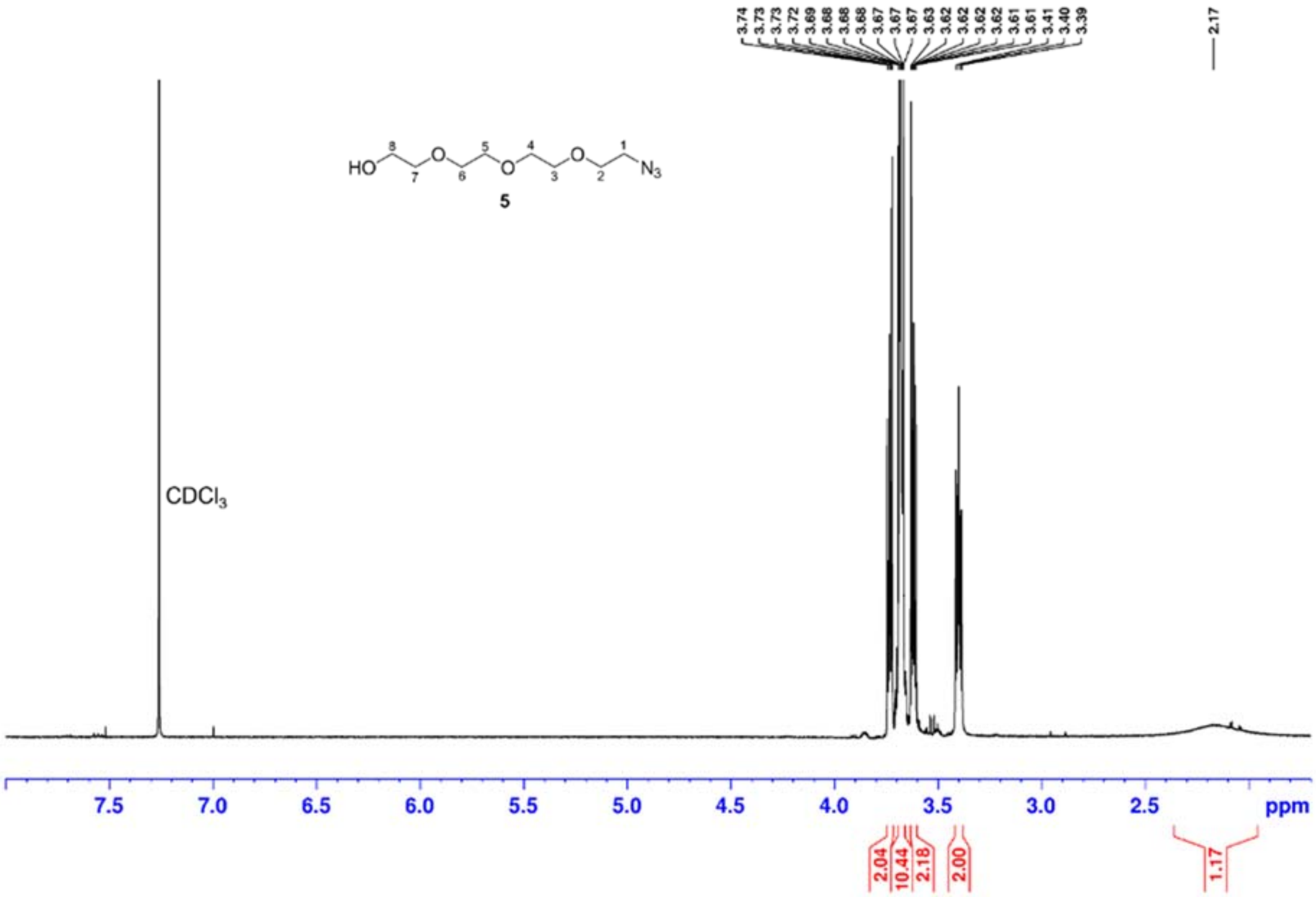

Figure S4. ^{1}H-NMR spectrum (400 MHz, $CDCl_3$) of compound **5**.

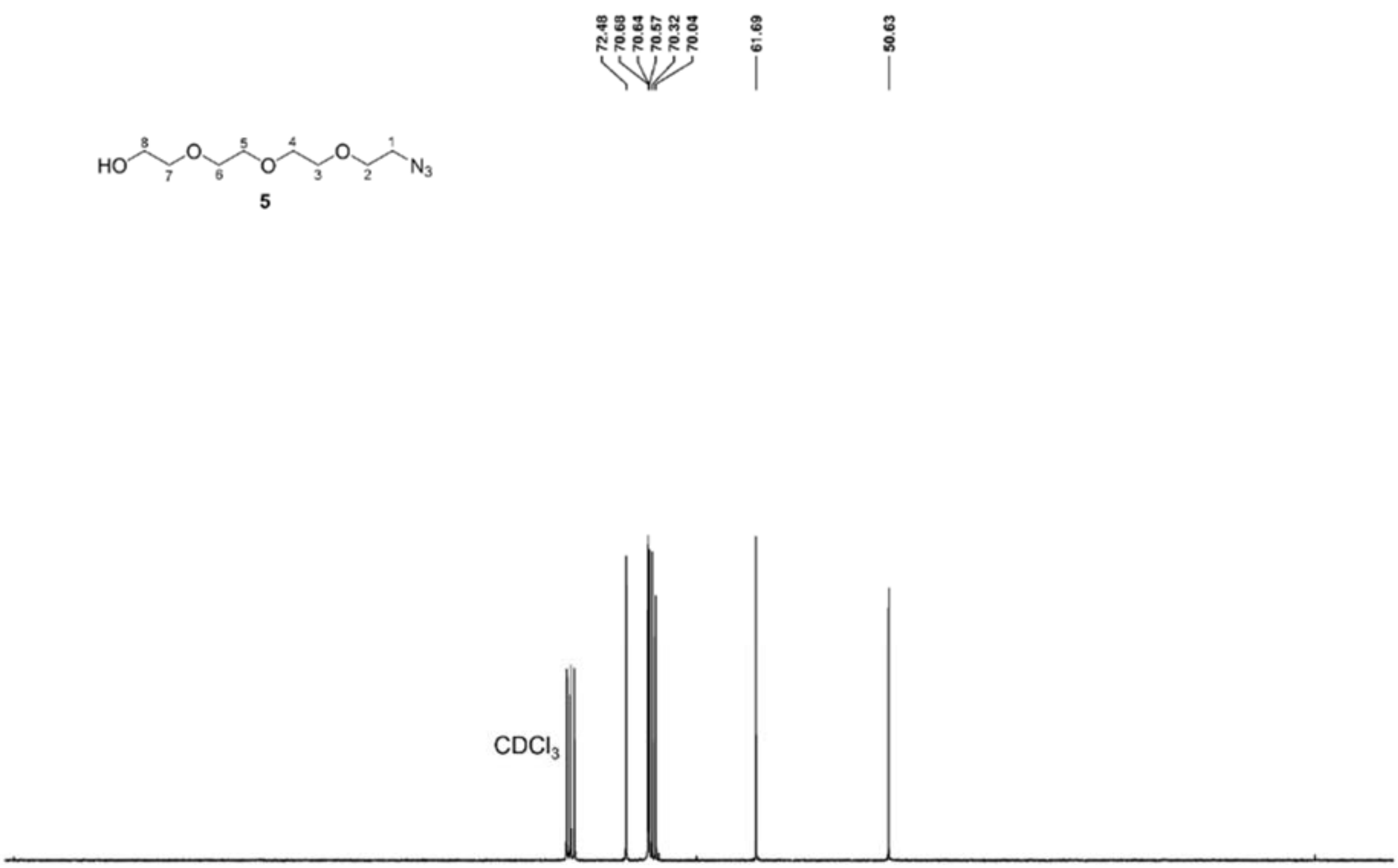

Figure S5. ^{13}C-NMR spectrum (100 MHz, $CDCl_3$) of compound **5**.

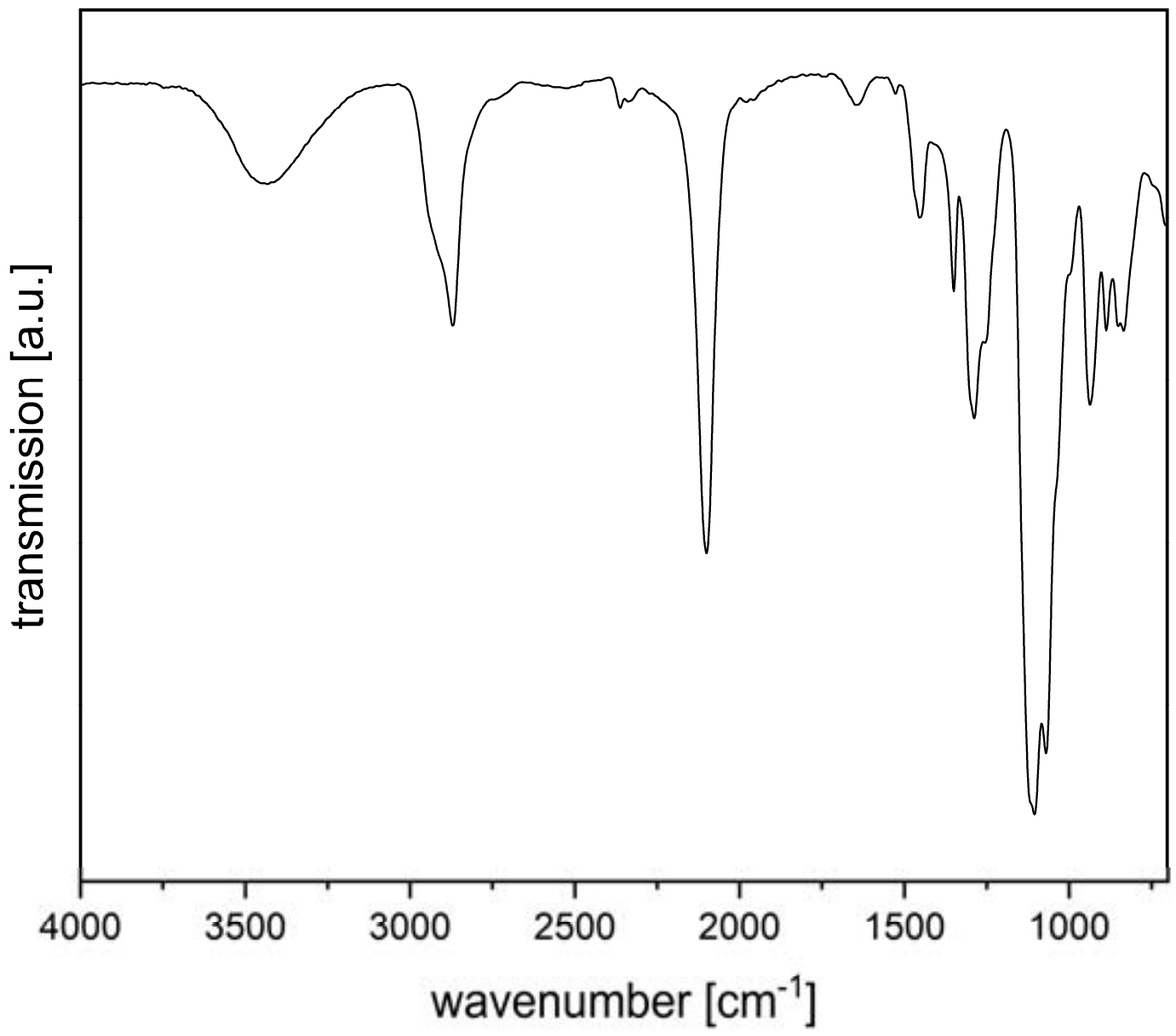

Figure S6. FT-IR (ATR) spectrum of compound **5**.

2-[2-[2-(2-Azidoethoxy)ethoxy]ethoxy]ethyl 4-methylbenzenesulfonate (**6**)[1]

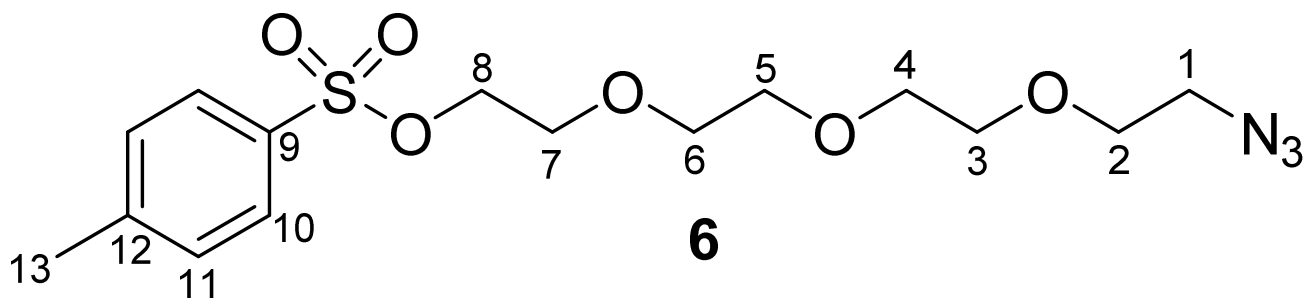

6

To a solution of 3.00 g of azide **5** (13.7 mmol, 1.00 eq) in 150 ml of dry dichloromethane 15.2 mL of triethylamine (109 mmol, 8.00 eq) were added. After cooling the reaction mixture to 0 °C, 20.7 g of tosyl chloride (109 mmol, 8.00 eq) were added and the mixture was stirred overnight at room temperature. Upon reaction completion, the solvent was removed *in vacuo* and the slightly reddish residue was dispersed in 100 mL of hydrochloric acid (2 N). The obtained suspension was extracted with dichloromethane (4x, 150 mL each) and the combined organic layers were dried over $MgSO_4$. After removal the solvent *in vacuo*, the crude product was purified by column chromatography (silica, cyclohexane/ethyl acetate, 1:2, v/v). The product **6** was obtained as a colourless oil.

Yield: 5.11 g (13.7 mmol, 98 %). **R_f** 0.60 (cyclohexane/ethyl acetate, 1:2). **^{1}H-NMR** (400 MHz, $CDCl_3$): δ = 7.80 (m, 2H, H-10), 7.34 (m, 2H, H-11), 4.16 (m, 2H, H-8), 3.70 – 3.60 (m, 12H, H-2–7), 3.38 (m, 2H, H-1), 2.45 (s, 3H, H-13) ppm. **^{13}C-NMR** (100 MHz, $CDCl_3$): δ = 145.0 (C_q, C-9), 133.1 (C_q, C-12), 130.0 (C_t, C-10), 128.1 (C_t, C-11), 70.9, 70.8, 70.7, 70.2,

69.4, 68.8 (C_s, C-2–8), 50.8 (C_s, C-1), 21.8 (C_p, C-13) ppm. **FT-IR** (ATR): ṽ = 2870 (w, ν(C-H)), 2101 (s, ν(N_3)), 1598 (w, ν(C=C_{arom})), 1451 (w, δ(C-H)), 1354 (m, ν(S=O)), 1290 (w), 1250 (w), 1175 (s, ν(C=O_{ether})), 1097 (s, ν(C=O_{ether})), 1014 (m), 918 (vs), 815 (m, δ(C-H_{arom})), 773 (m), 662 (s), 552 (vs) cm^{-1}.

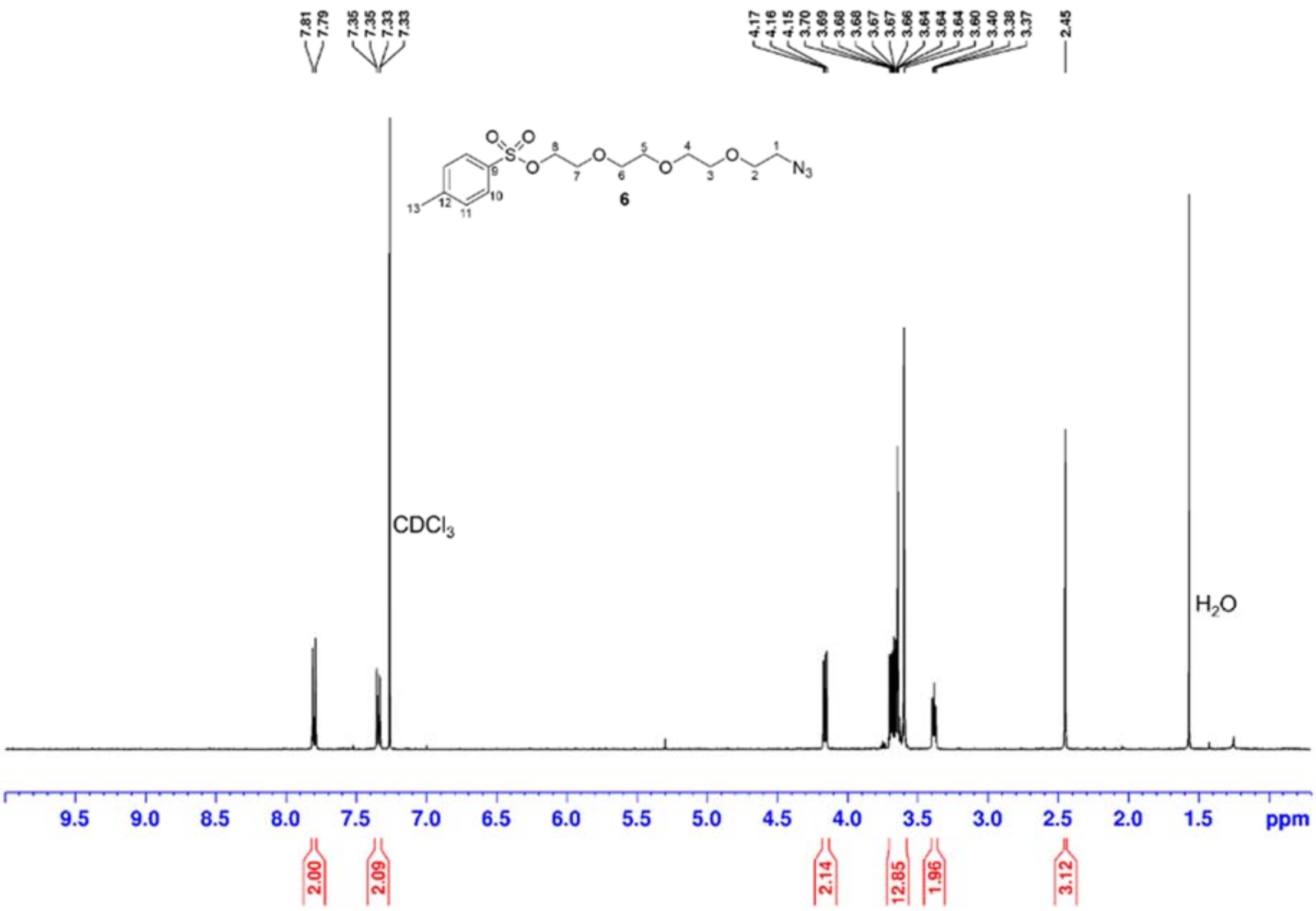

Figure S7. ^{1}H-NMR spectrum (400 MHz, $CDCl_3$) of compound **6**.

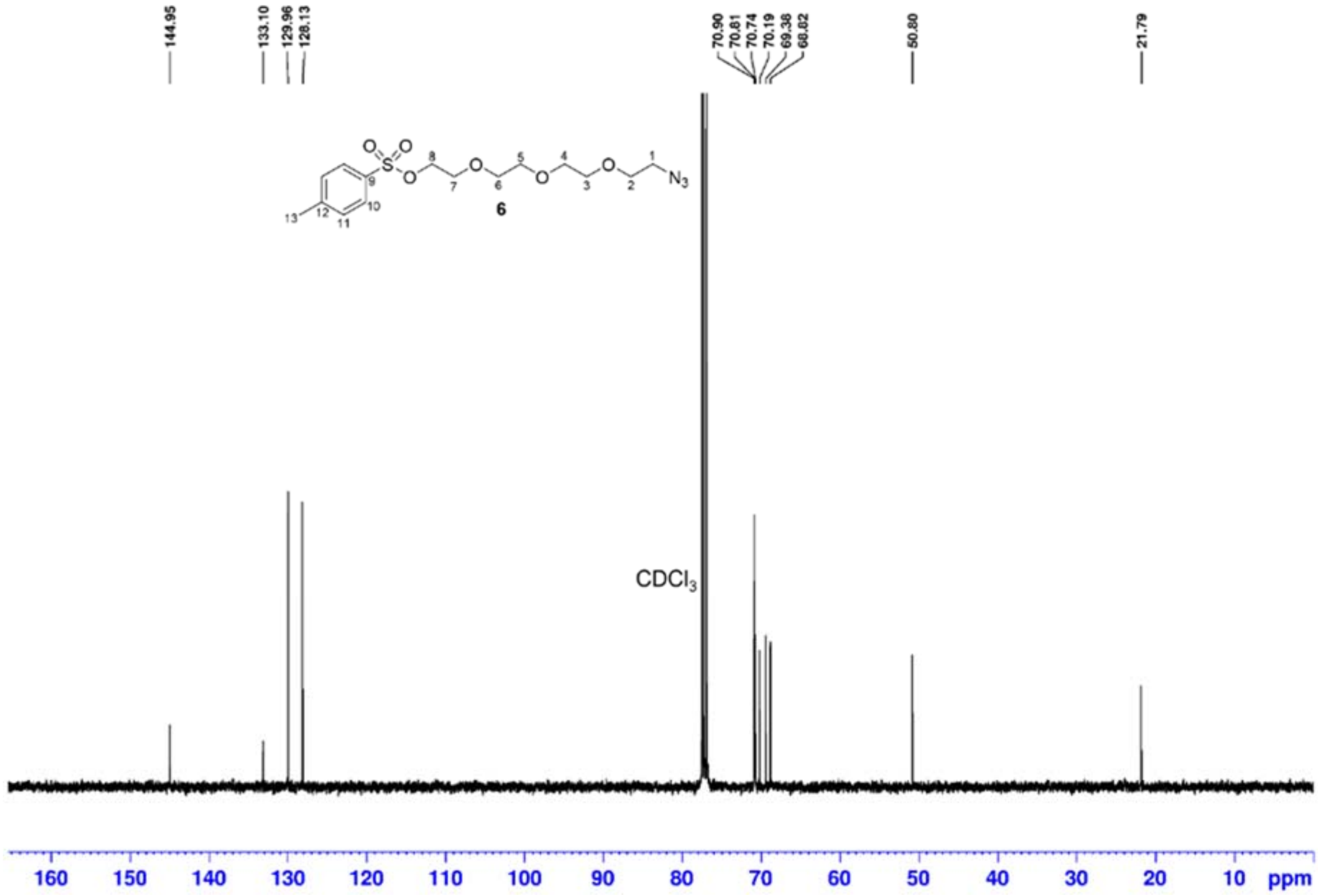

Figure S8. ^{13}C-NMR spectrum (100 MHz, $CDCl_3$) of compound **6**.

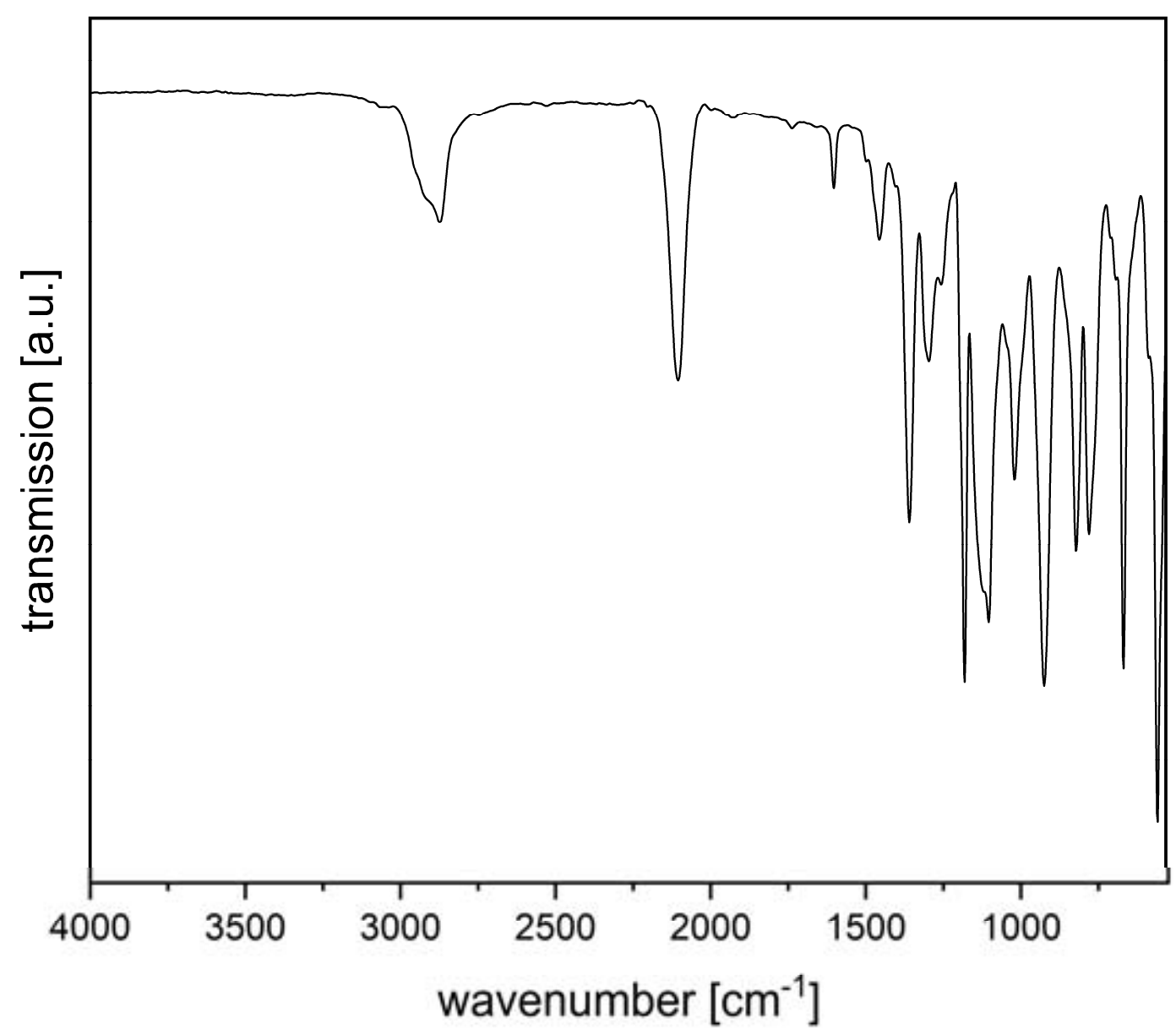

Figure S9. FT-IR (ATR) spectrum of compound **6**.

2,2-Dimethyl-4-oxo-3,8,11,14-tetraoxa-5-azahexadecan-16-yl-4-methylbenzenesulfonate (**7**)[1]

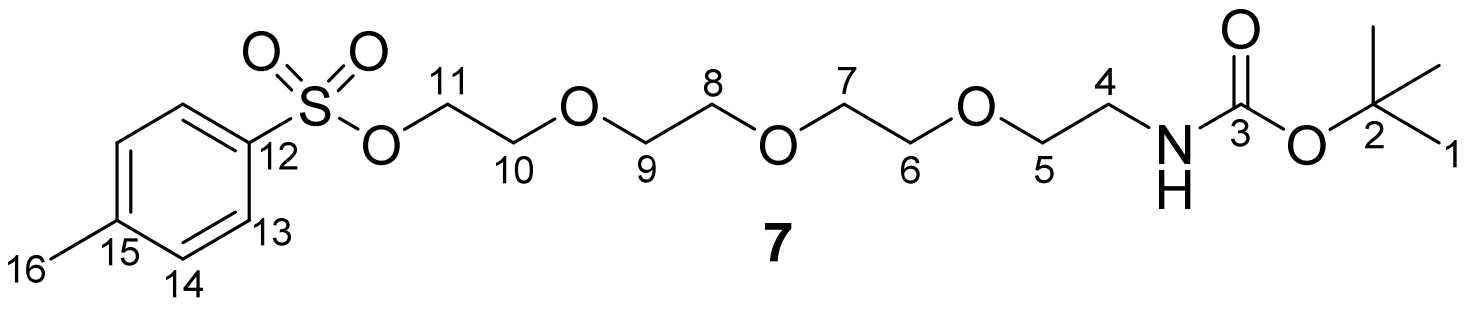

7

To a solution of 2.30 g of azido tetraethylene glycol tosylate **6** (6.16 mmol, 1.00 eq) in 15 mL of dry methanol 0.50 g of 10 %-Pd/C (0.20 eq) were added, and the reaction mixture was cooled to 0 °C in an ice bath. After subsequent addition of 3.63 g of Boc anhydride (16.6 mmol, 2.70 eq) the dispersion was stirred under H_2-atmosphere for 20 h. Then, the reaction mixture was filtered and the solvent removed *in vacuo*. The crude product was purified by column chromatography (silica, cyclohexane/ethyl acetate, 1:1, v/v) giving the product **7** as pale-yellow oil.

Yield: 2.19 g (4.89 mmol, 79 %). **R_f** 0.30 (cyclohexane/ethyl acetate, 1:1). **^{1}H-NMR** (400 MHz, $CDCl_3$): δ = 7.80 (m, 2H, H-10), 7.34 (m, 2H, H-11), 4.99 (br, 1H, N*H*), 4.16 (m, 2H, H-11), 3.71 – 3.68 (m, 2H, H-10), 3.61 – 3.58 (m, 8H, H-6–9), 3.54 – 3.51 (m, 2H, H-5), 3.32 – 3.29 (m, 2H, H-4), 2.45 (s, 3H, H-16), 1.43 (s, 9H, H-1) ppm. **^{13}C-NMR** (100 MHz, $CDCl_3$): δ = 156.1 (C_q, C-3), 145.0 (C_q, C-12), 133.1 (C_q, C-15), 130.0 (C_t, C-13), 128.1 (C_t, C-14), 79.3 (C_q, C-2), 70.9, 70.7, 70.6, 70.3, 69.4, 68.8 (C_s, C-5–11), 40.5 (C_s, C-4), 28.6 (C_p, C-1), 21.8 (C_p, C-16) ppm. **FT-IR** (ATR): ṽ = 3357 (br, ν(n-H)), 2871 (w, ν(C-H)), 1707 (s, ν(C=O), amide I), 1597 (w, ν($C=C_{arom}$)), 1512 (m, ν(C-N), amide II), 1454 (w, δ(C-H)), 1358 (m, ν(S=O)), 1248 (w), 1173 (s, ν($C=O_{ether}$)), 1097 (s, ν($C=O_{ether}$)), 1014 (m), 918 (s), 864 (w), 816 (m, δ($C-H_{arom}$)), 773 (m) cm^{-1}.

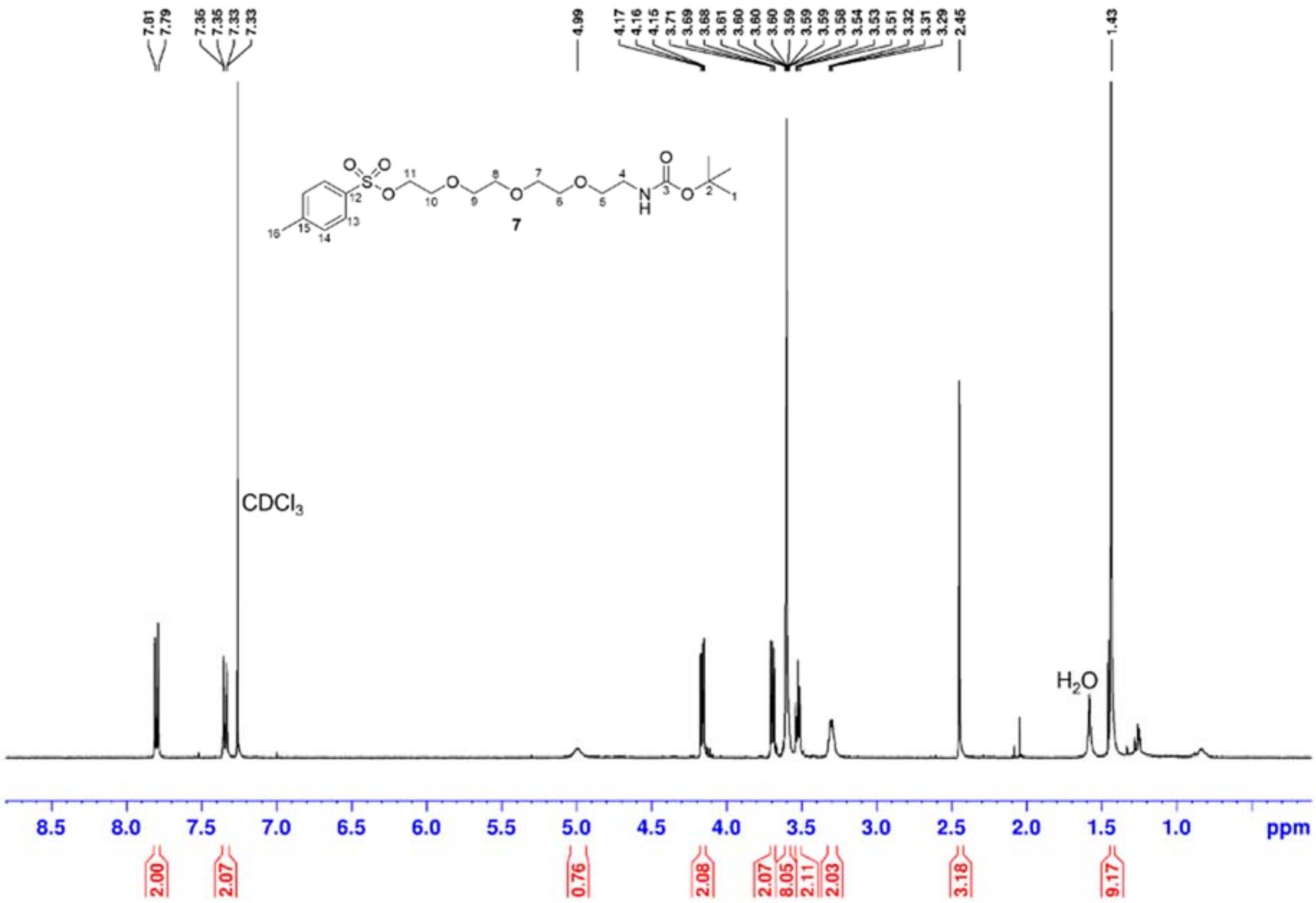

Figure S10. ^{1}H-NMR spectrum (400 MHz, $CDCl_3$) of compound **7**.

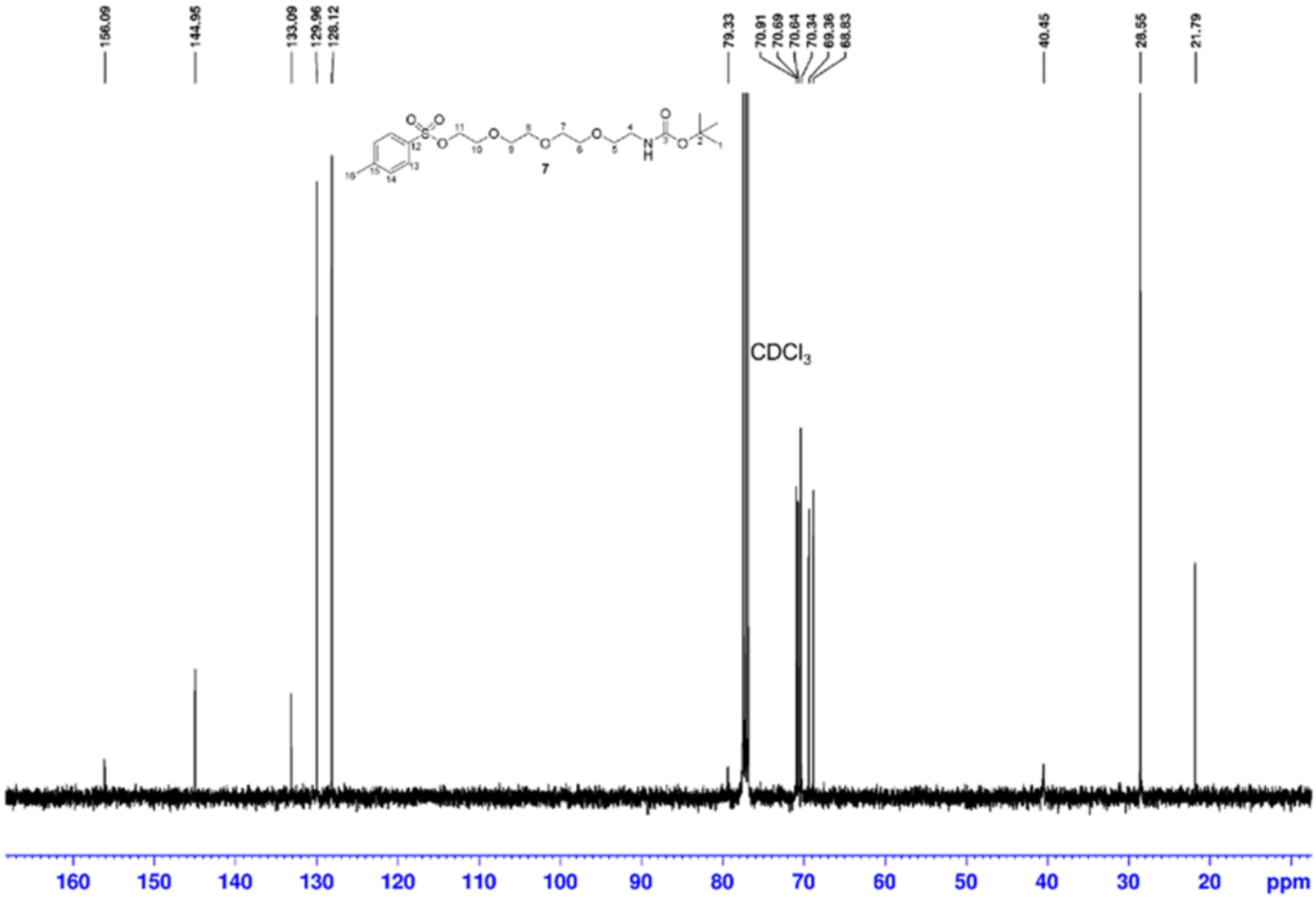

Figure S11. ^{13}C-NMR spectrum (100 MHz, $CDCl_3$) of compound **7**.

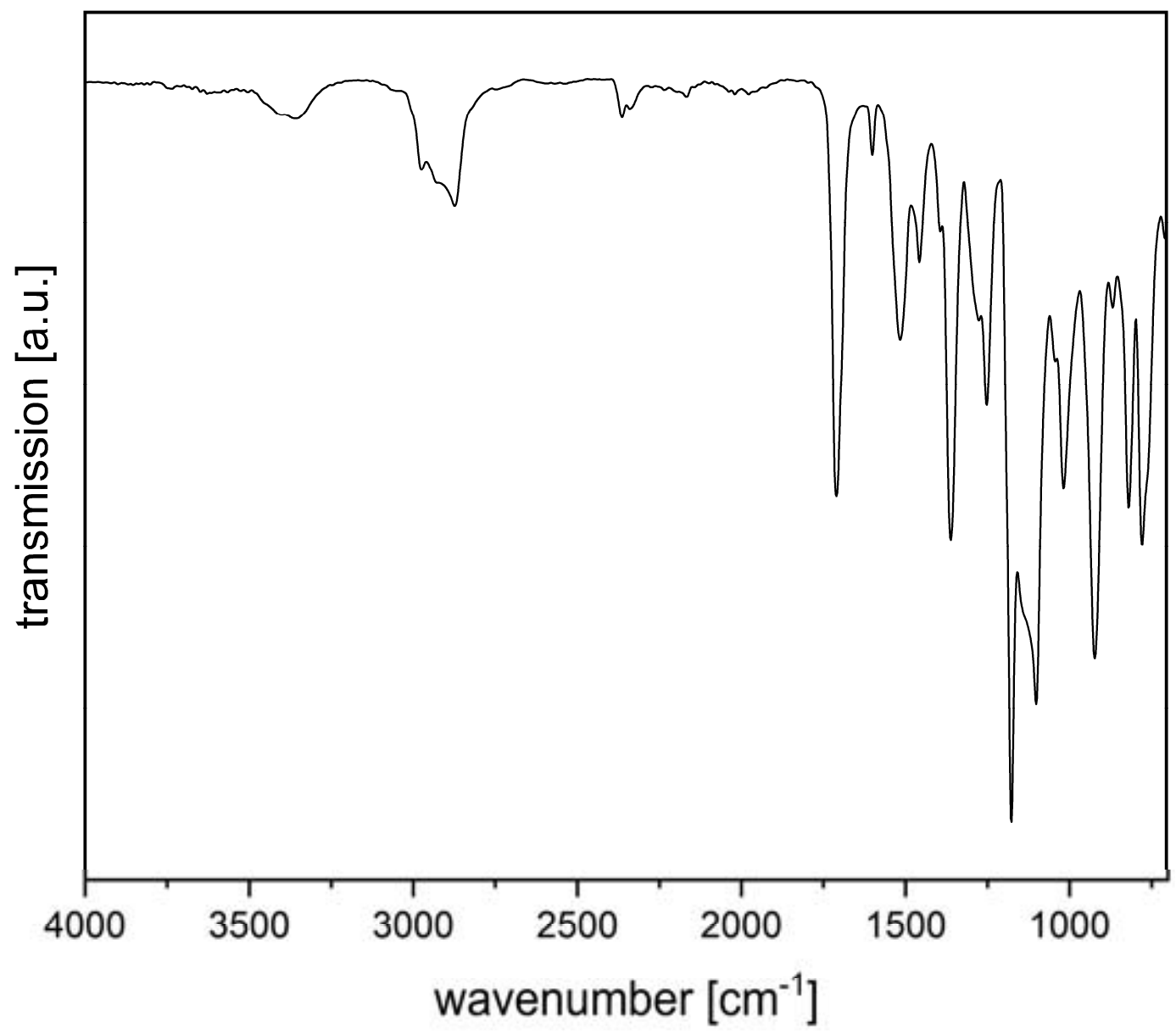

Figure S12. FT-IR (ATR) spectrum of compound **7**.

tert-Butyl [2-[2-[2-(2-Azidoethoxy)ethoxy]ethoxy]ethyl]carbamate (**8**)[2]

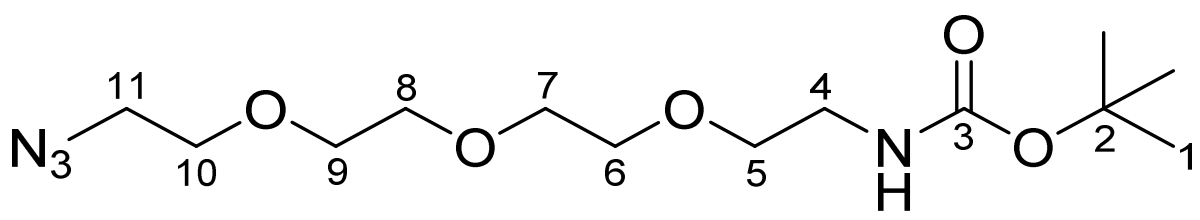

8

1.14 g of tosylate **7** (2.55 mmol, 1.00 eq) were dissolved in 20 mL of dry dimethylformamide (DMF) and 0.83 g of sodium azide (12.8 mmol, 5.00 eq) were added. After stirring at 70 °C for 14 h, the solvent was removed *in vacuo* followed by addition of 50 mL of water and 50 mL of dichloromethane. The aqueous phase was extracted three times with dichloromethane (50 mL each), and the combined organic phases were dried over $MgSO_4$. After removing the solvent *in vacuo*, the crude product was filtered for further purification through a column (silica, cyclohexane/ethyl acetate, 1:1, v/v). The filtrate was concentrated *in vacuo* to obtain the product **8** as a colourless oil.

Yield: 779 mg (2.45 mmol, 96 %). **R$_f$** 0.37 (cyclohexane/ethyl acetate, 1:1). **^{1}H-NMR** (400 MHz, $CDCl_3$): δ = 5.02 (br, 1H, N*H*), 3.70 – 3.62 (m, 10H, H-6–10), 3.55 – 3.53 (m, 2H, H-5), 3.54 – 3.51 (m, 2H, H-5), 3.32 – 3.29 (m, 2H, H-4), 2.45 (s, 3H, H-16), 1.43 (s, 9H, H-1) ppm. **^{13}C-NMR** (100 MHz, $CDCl_3$): δ = 156.1 (C_q, C-3), 79.3 (C_q, C-2), 70.9, 70.8, 70.8, 70.4, 70.3, 70.2 (C_s, C-5–10), 50.8 (C_s, C-11), 40.3 (C_s, C-4), 28.5 (C_p, C-1) ppm. **FT-IR** (ATR): ṽ = 3358 (br, ν(n-H)), 2974 (w, ν(C-H)), 2870 (w, ν(C-H)), 2100 (m, ν(N_3)), 1707 (s, ν(C=O), amide I), 1512 (m, ν(C-N), amide II), 1454 (w, δ(C-H)), 1390 (w), 1365 (w), 1275

(m), 1248 (m), 1169 (s, ν(C=O_{ether})), 1107 (vs, ν(C=O_{ether})), 1039 (w), 937 (w), 862 (w), 781 (m) cm^{-1}.

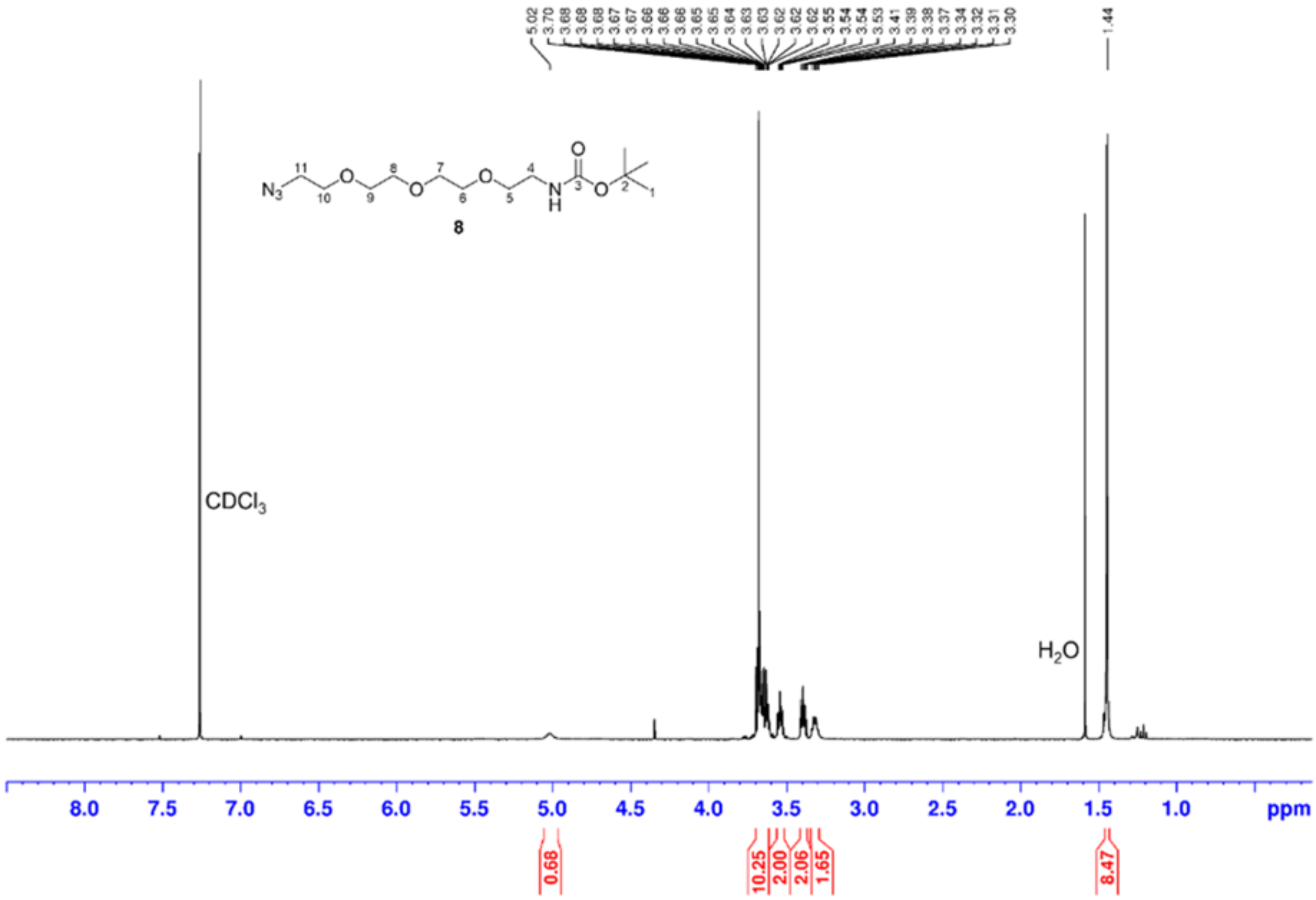

Figure S13. ^{1}H-NMR spectrum (400 MHz, $CDCl_3$) of compound **8**.

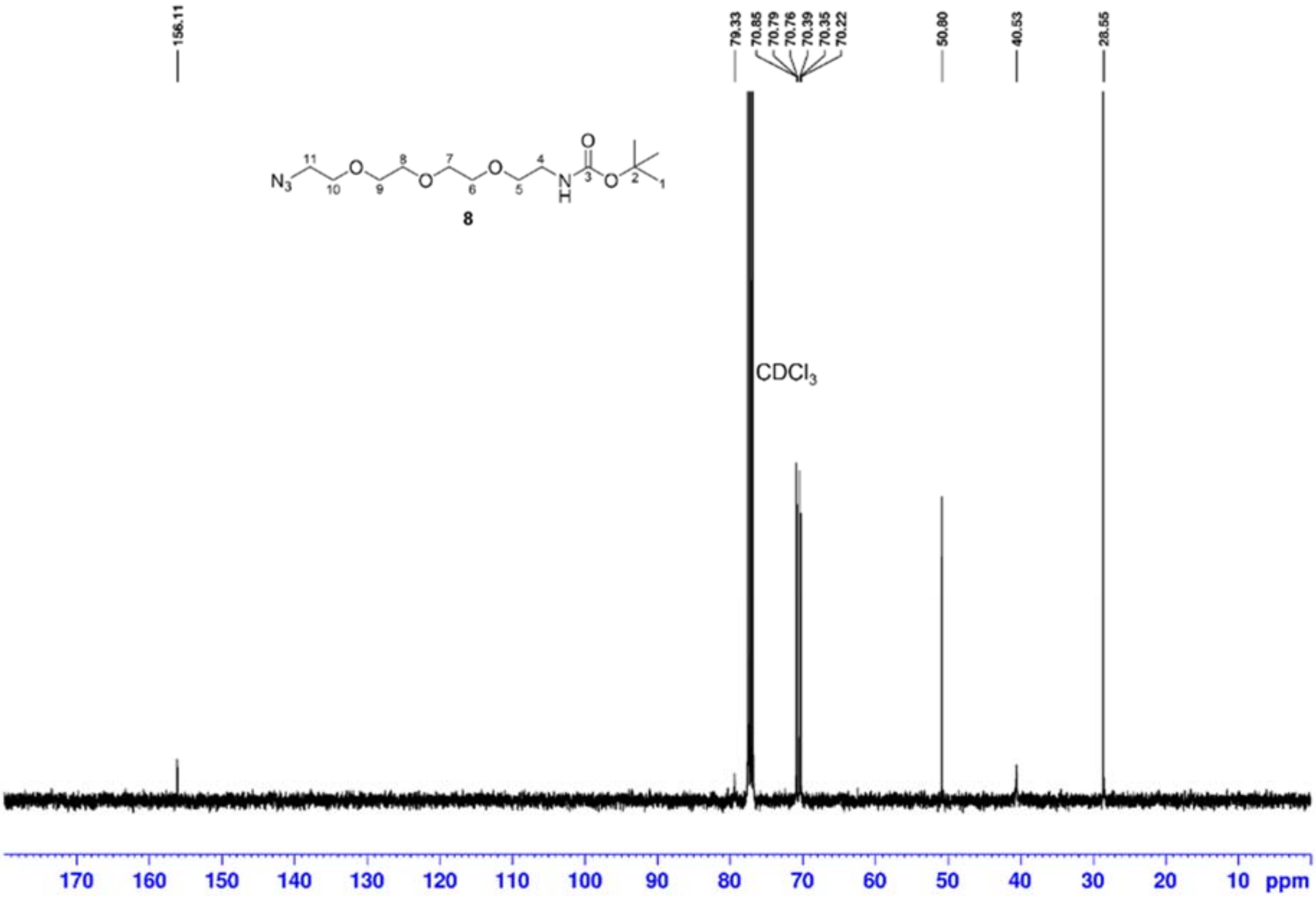

Figure S14. ^{13}C-NMR spectrum (100 MHz, $CDCl_3$) of compound **8**.

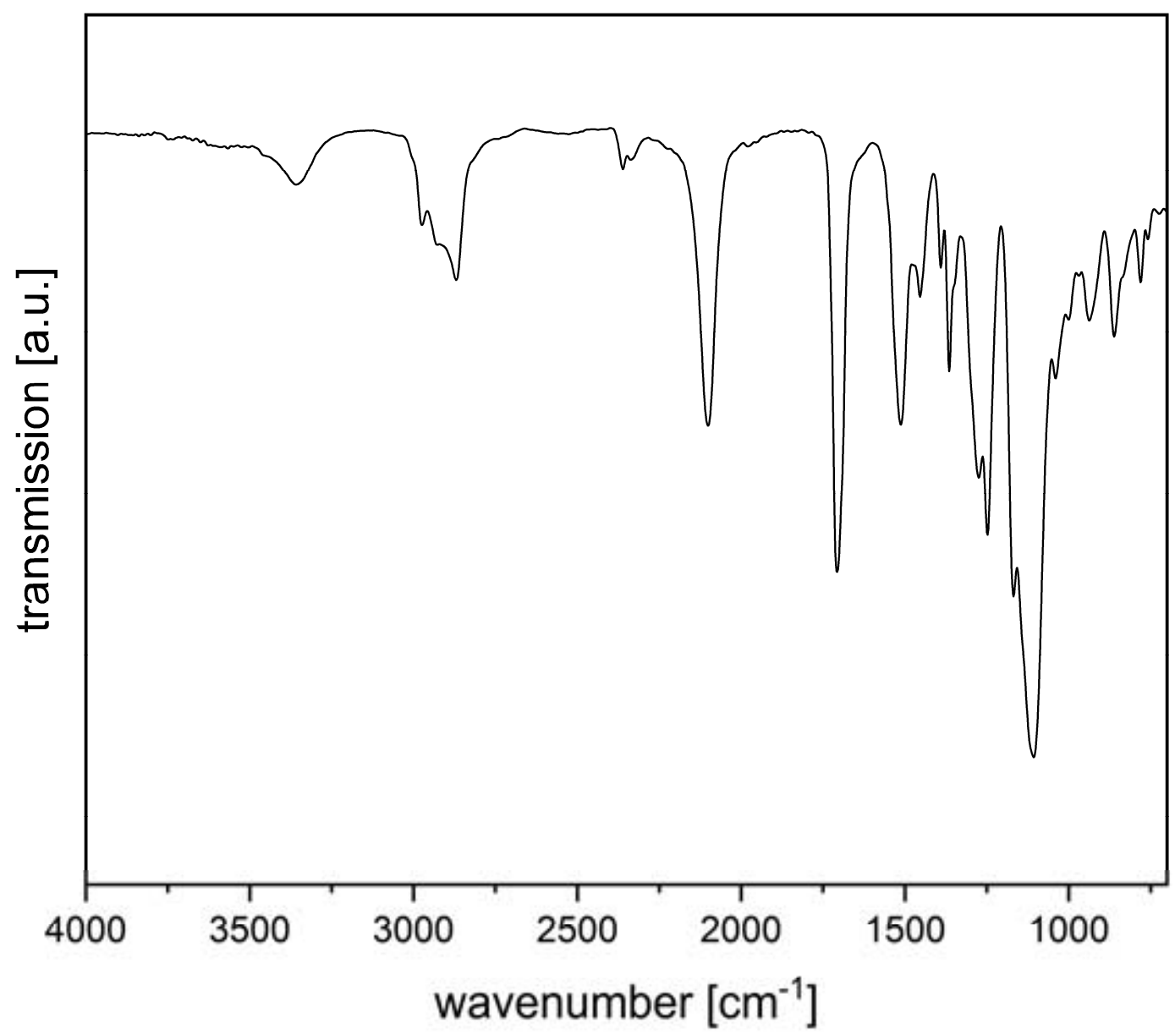

Figure S15. FT-IR (ATR) spectrum of compound **8**.

2-[2-[2-(2-Azidoethoxy)ethoxy]ethoxy]ethanamine (**2**)[2]

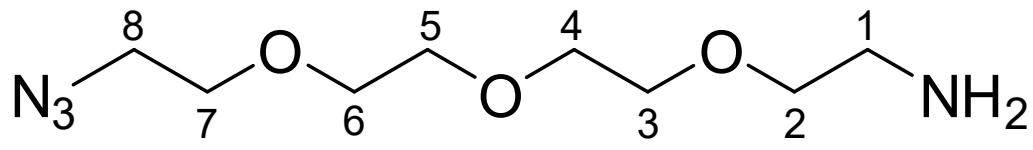

2

35.0 mL of trifluoroacetic acid were added to a solution of 614 mg of azide **8** (1.92 mmol, 1.00 eq) in 50 mL of chloroform. After stirring the reaction mixture for 2 h at room temperature, the volatiles were removed *in vacuo* and the residue was redissolved in chloroform. The resulting organic phase was washed three times with sat. $NaHCO_3$ solution, three times with NaCl solution and then dried over $MgSO_4$. The solvent was removed *in vacuo* to give the product **2** as pale-yellow oil.

Yield: 278 mg (1.66 mmol, 66 %). **R$_f$** 0.29 (dichloromethane/methanol/7 N NH_3, 95:4:1). **^{1}H-NMR** (400 MHz, $CDCl_3$): δ = 3.69 – 3.62 (m, 10H, H-3–7), 3.53 (t, $^3J_{2,1}$ = 5.20 Hz, 8H, H-2), 3.40 (t, $^3J_{8,7}$ = 5.20 Hz, 2H, H-8), 2.89 (t, $^3J_{1,2}$ = 5.20 Hz, 2H, H-1) ppm. **^{13}C-NMR** (100 MHz, $CDCl_3$): δ = 73.6 (C$_s$, C-2), 70.9, 70.8, 70.8, 70.4, 70.2 (C$_s$, C-3–7), 50.8 (C$_s$, C-8), 41.9 (C$_s$, C-1) ppm. **FT-IR** (ATR): ṽ = 3369 (br, ν(n-H)), 2866 (w, ν(C-H)), 2098 (s, ν(N_3)), 1595 (m, ν(N-H)), 1452 (w, δ(C-H)), 1348 (w), 1286 (m), 1105 (s, ν(C=O$_{ether}$)), 929 (m), 850 (w) cm^{-1}.

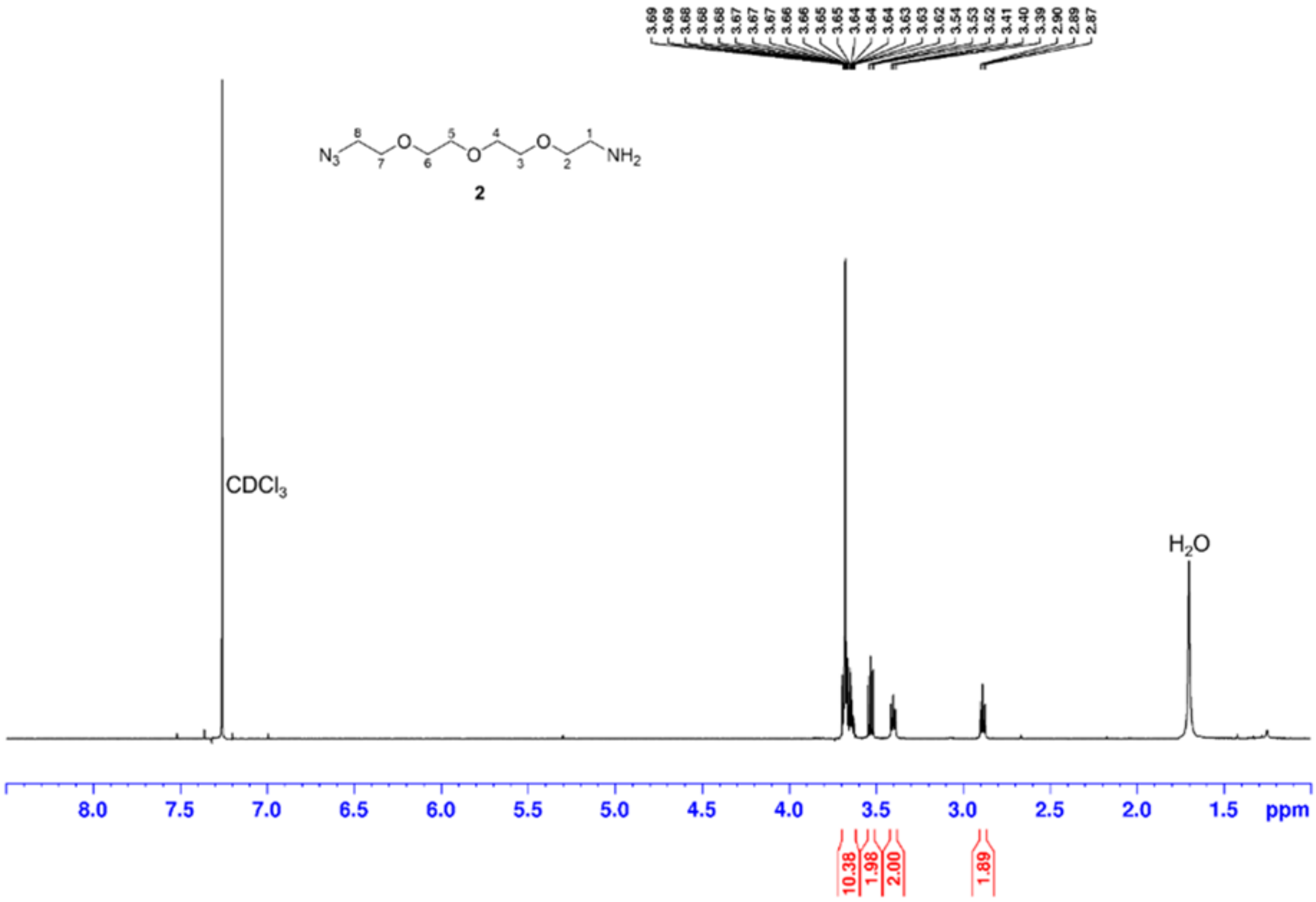

Figure S16. ^{1}H-NMR spectrum (400 MHz, $CDCl_3$) of compound **2**.

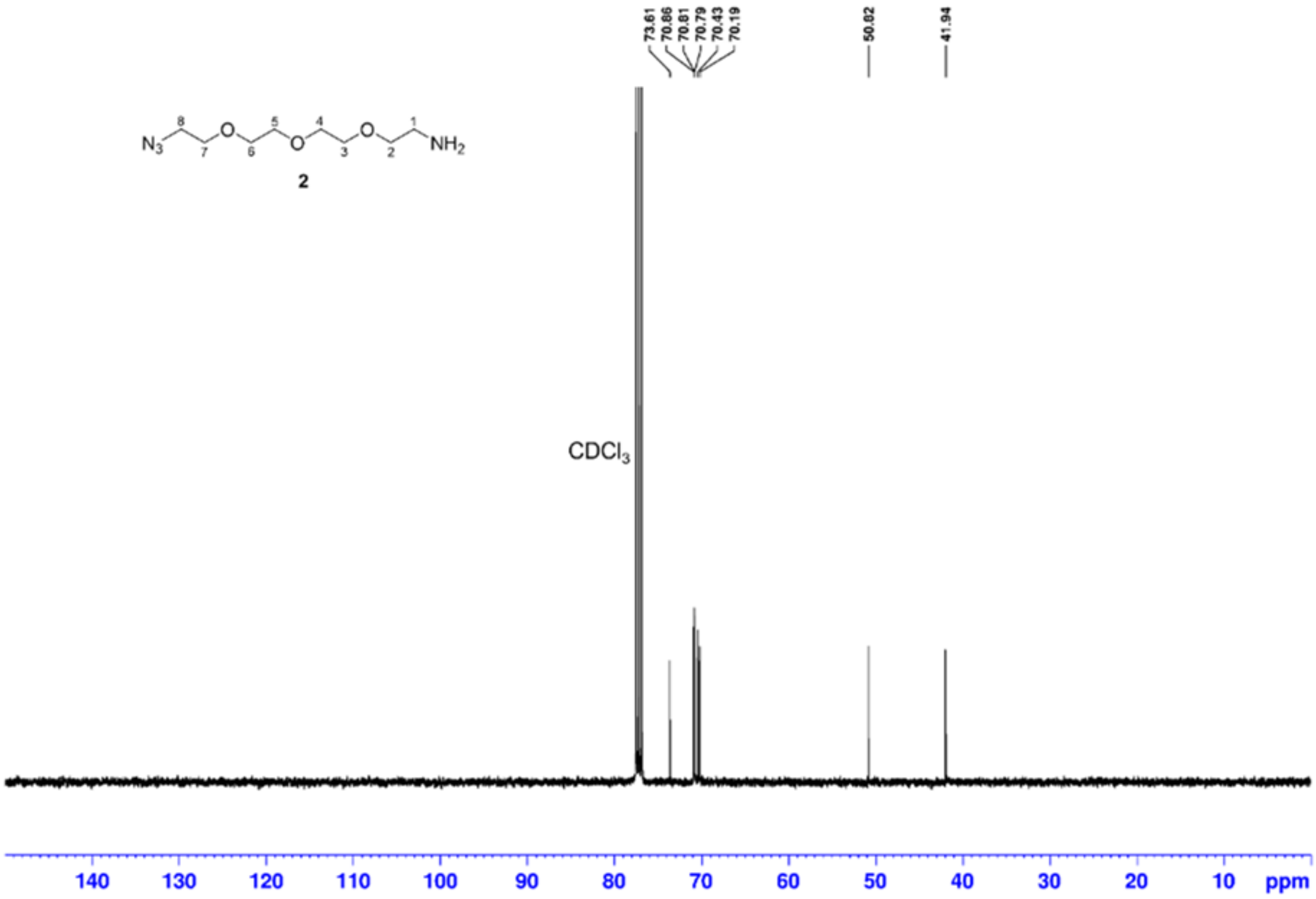

Figure S17. ^{13}C-NMR spectrum (100 MHz, $CDCl_3$) of compound **2**.

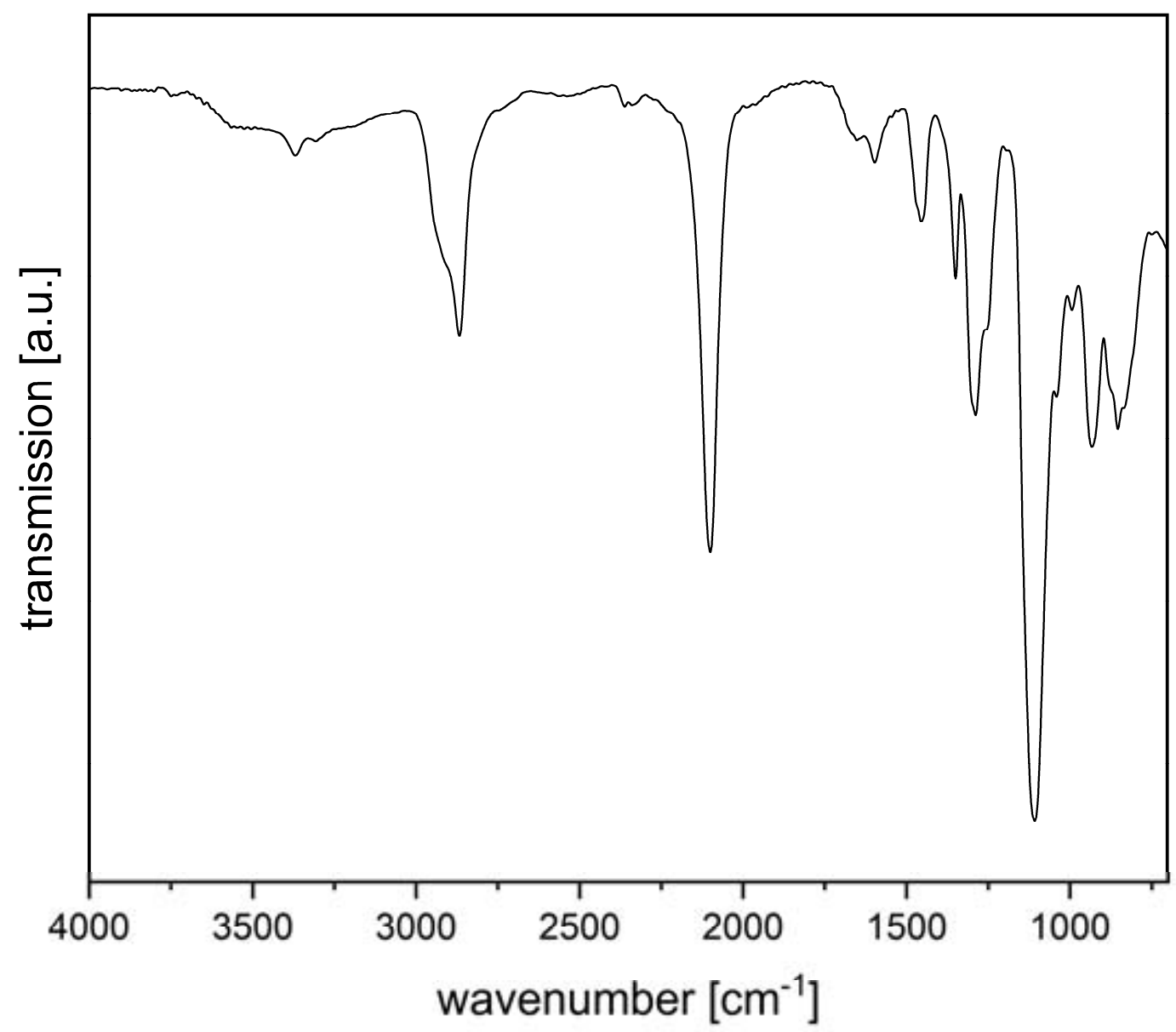

Figure S18. FT-IR (ATR) spectrum of compound **2**.

2. Synthesis and characterization of $[Mn(CO)_3(tpm^{CH2OCH2C≡CH})]PF_6$ (CORM)

All reactions were carried out in anhydrous glass flasks heated to 130 °C for a couple of hours and – where necessary – under a protective atmosphere of argon. Commercially available chemicals were purchase from Sigma-Aldrich and Strem and used as received. Tris-2,2,2-(pyrazol-1-yl)ethoxypropargylether ($tpm^{CH2OCH2C≡CH}$) was synthesized following a published procedure.[3-6] NMR spectra were recorded on a Bruker Avance III Nanobay spectrometer (^{1}H at 400.47 MHz) and referenced against the proton signal of the solvent.[7] Chemical shifts δ are reported in ppm and coupling constants *J* are given in Hz. The multiplicity of the peaks is indicated as singlet (s), doublet (d), or triplet (t). The elemental composition of the compounds was determined with a CHN analyzer Vario Micro Cube from Elementar Analysensysteme GmbH.

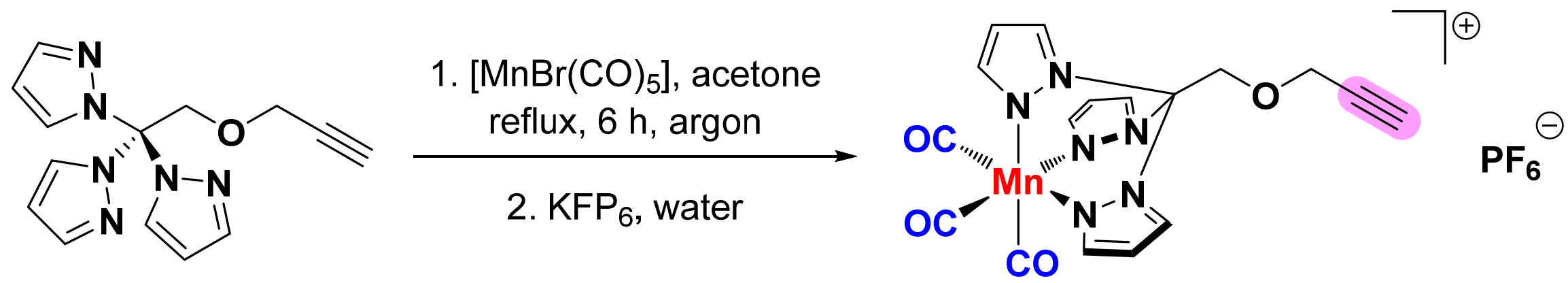

$C_{14}H_{14}N_6O$
282.30 g/mol

$C_{17}H_{14}F_6MnN_6O_4P$
566.23 g/mol

In a 100 mL Schlenk flask, tris-2,2,2-(pyrazol-1-yl)ethoxypropargylether (530 mg, 1.9 mmol) was dissolved in anhydrous acetone (15 mL) and degassed by purging with argon. Then, under exclusion of light, manganese pentacarbonyl bromide (517 mg, 1.9 mmol) was added and the solution heated to reflux for 6 h, during which a yellow precipitate started to form. After cooling to room temperature, the resulting solid material was filtered off and redissolved in methanol (10 mL). Addition of potassium hexafluorophosphate (346 mg, 1.9 mmol) in water (10 mL) led to formation of a yellow precipitate, which was filtered off and washed with water (3 × 10 mL). After drying under high vacuum for 24 h, the product was obtained as a pale-yellow solid.

Yield: 569 mg (1.0 mmol, 53%). **^{1}H-NMR** (400.47 MHz, DMSO-*d*6): δ = 8.71 (s, 3H, pyrazole-H3), 8.59 (s, 3H, pyrazole-H5), 6.79 (s, 3H, pyrazole-H4), 5.59 (s, 2H, C*H*2O), 4.72 (d, 2H, ^4J = 2.3 Hz, OC*H*2C≡CH), 3.84 (t, 1H, ^4J = 2.2 Hz, C≡C*H*) ppm. **Elemental analysis** (%): calcd. for $C_{17}H_{14}F_6MnN_6O_4P$: C 36.06, H 2.49, N 14.84; found (%): C 36.01, H 2.55, N 14.93.

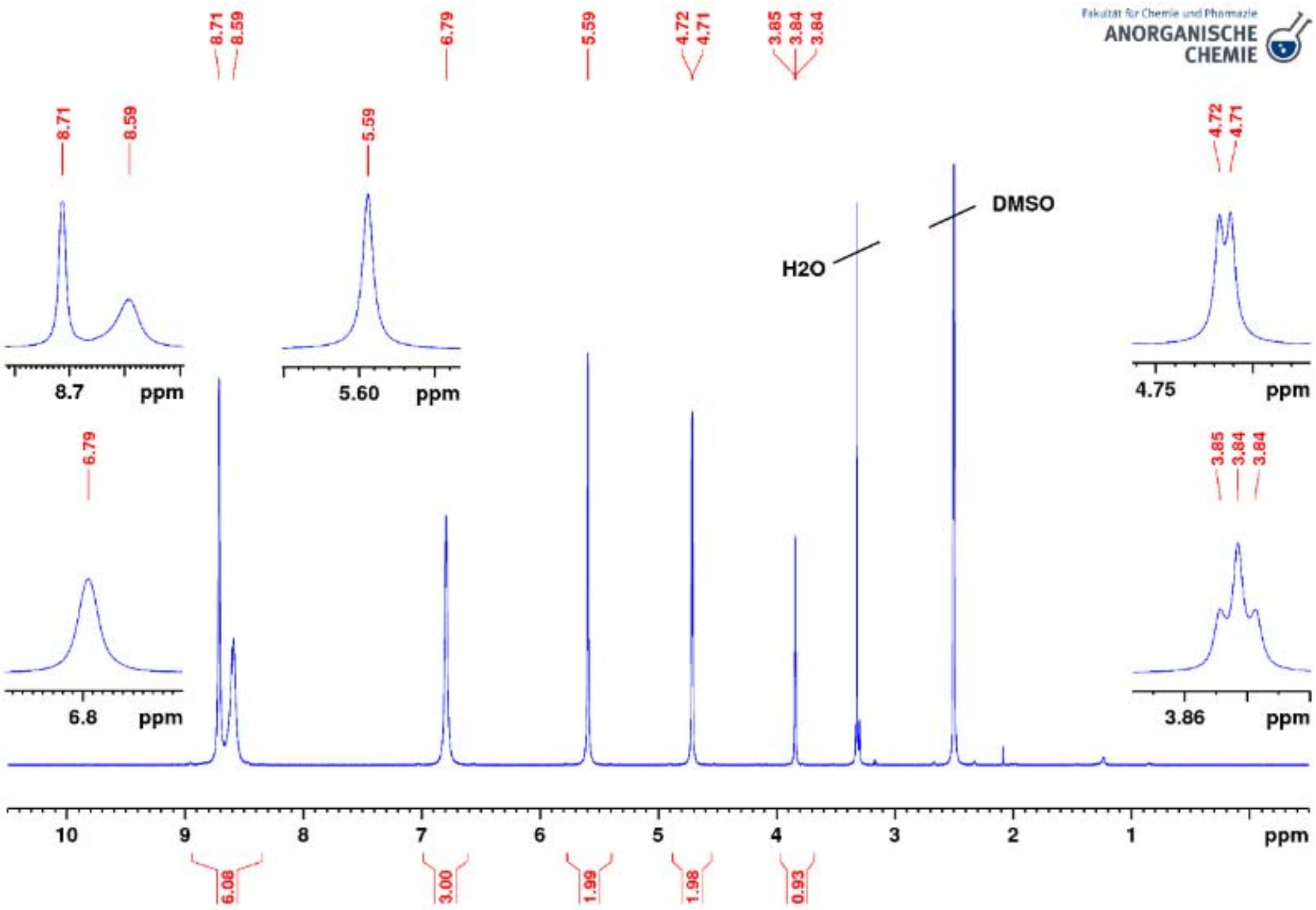

Figure S19. ^{1}H-NMR spectrum (400 MHz, DMSO-d_6) of the alkyne-functionalized **CORM**.

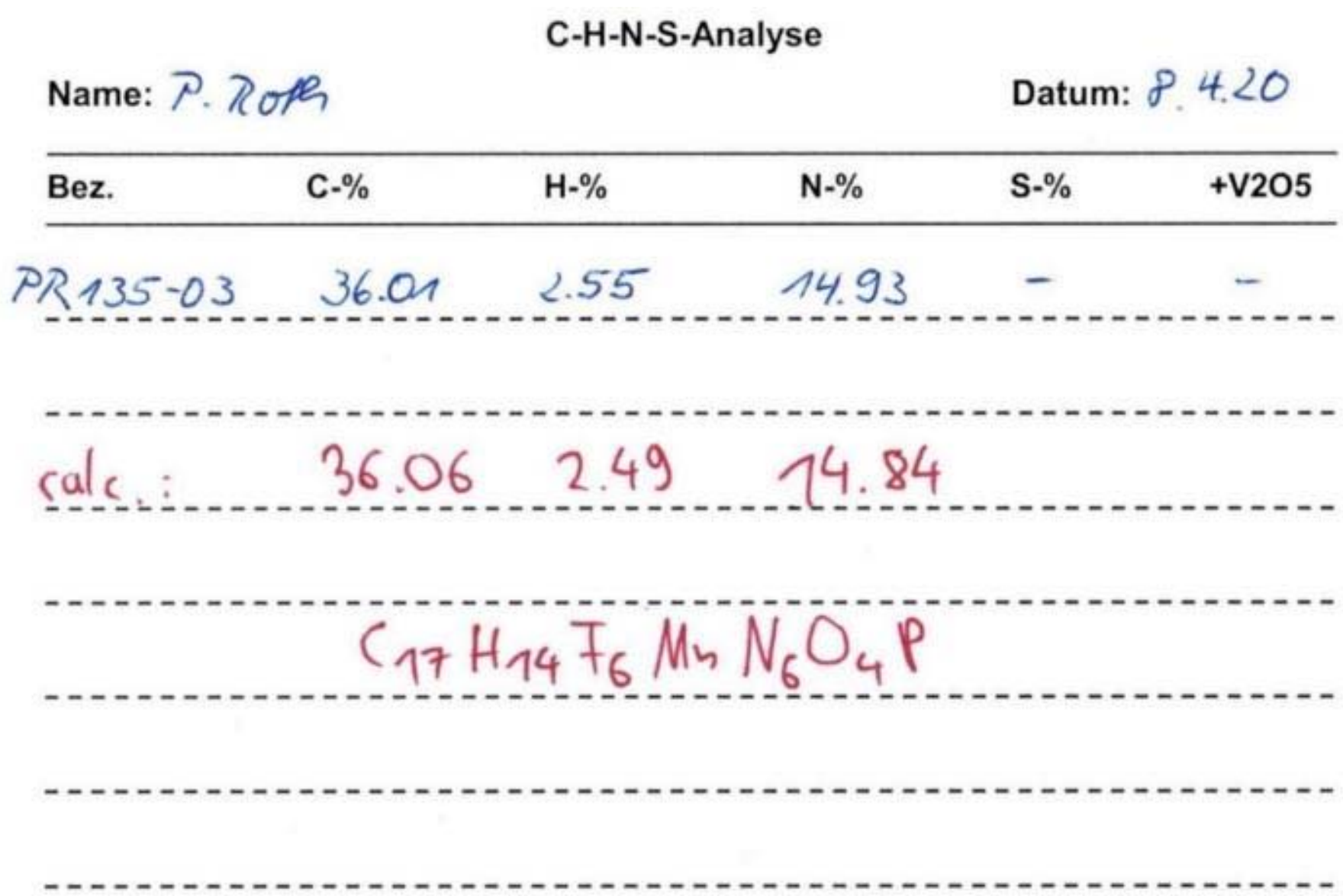

C-H-N-S-Analyse

Name: P. Roth

Datum: 8.4.20

Bez.	C-%	H-%	N-%	S-%	+V2O5
PR135-03	36.01	2.55	14.93	–	–
calc.:	36.06	2.49	14.84		

$C_{17}H_{14}F_6MnN_6O_4P$

Figure S20. Elemental analysis of the alkyne-functionalized **CORM**.

3. Characterization of functionalized NDs

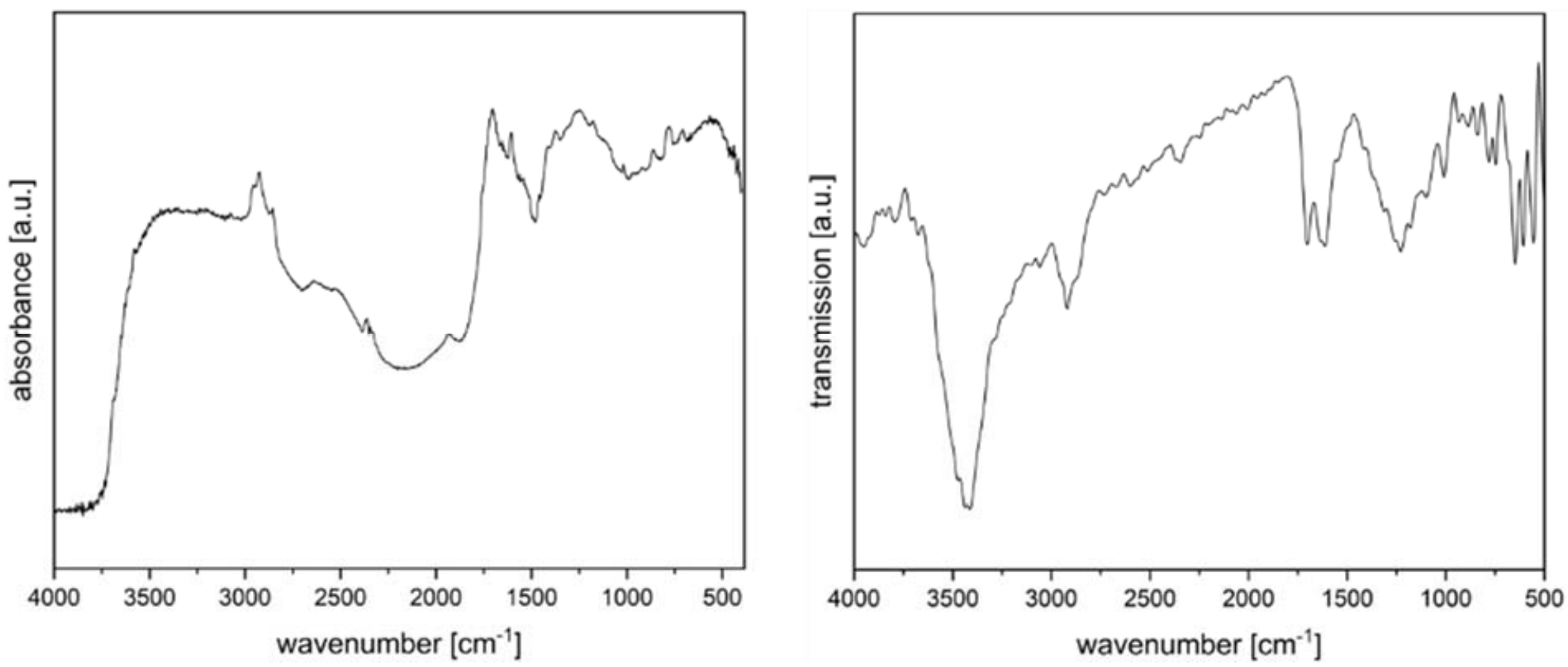

Figure S21. DRIFT spectrum (left) and FT-IR (KBr pellet) spectrum (right) of benzoic acid functionalized nanodiamond **ND-CO_2H**.

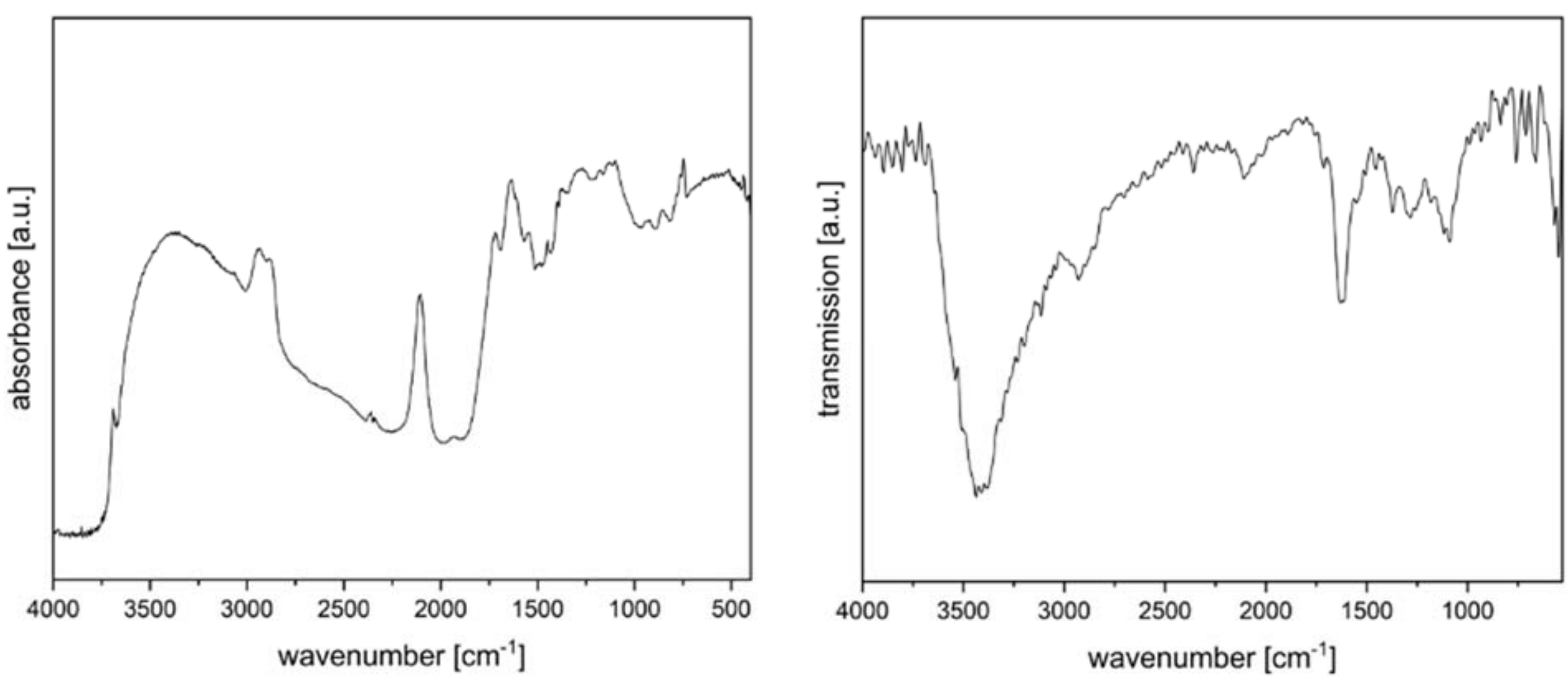

Figure S22. DRIFT spectrum (left) and FT-IR (KBr pellet) spectrum (right) of azide linker functionalized nanodiamond **ND-Linker**.

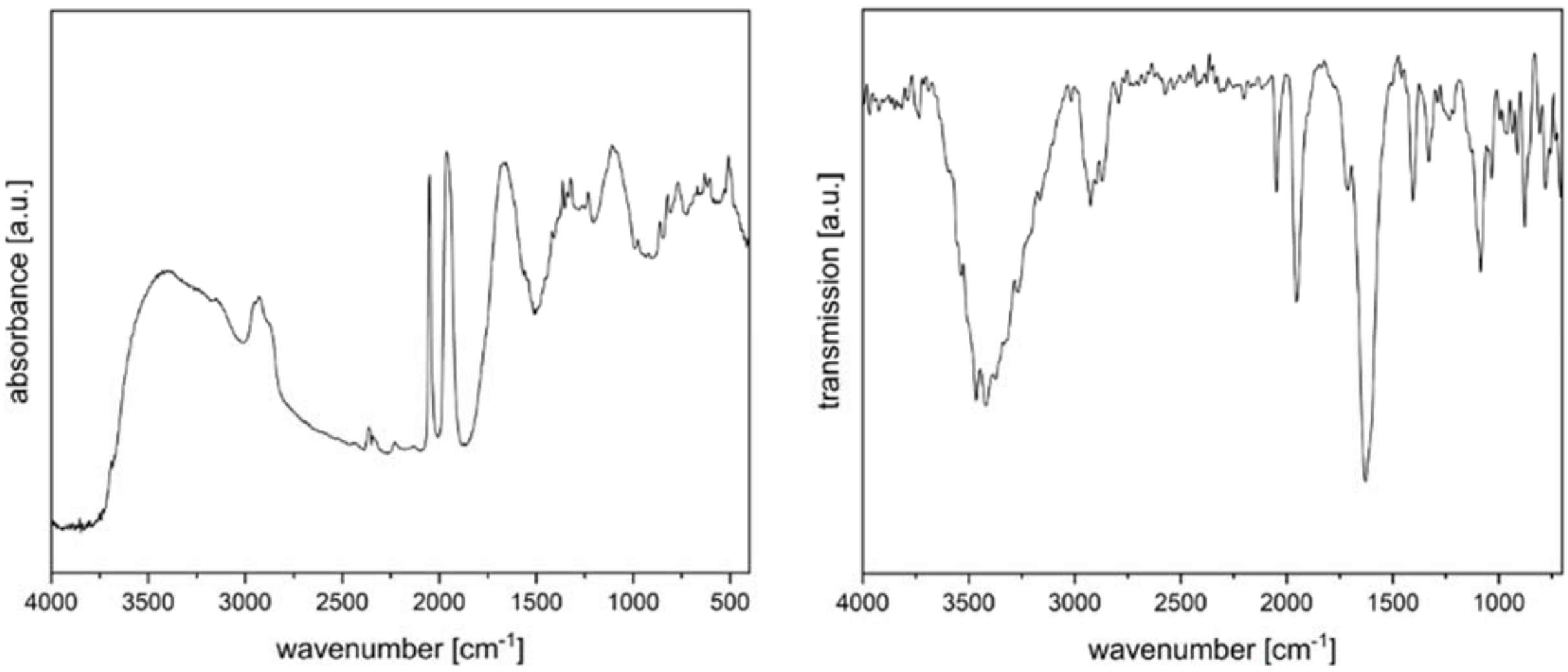

Figure S23. DRIFT spectrum (left) and FT-IR (KBr pellet) spectrum (right) of PhotoCORM functionalized nanodiamond **ND-CORM**.

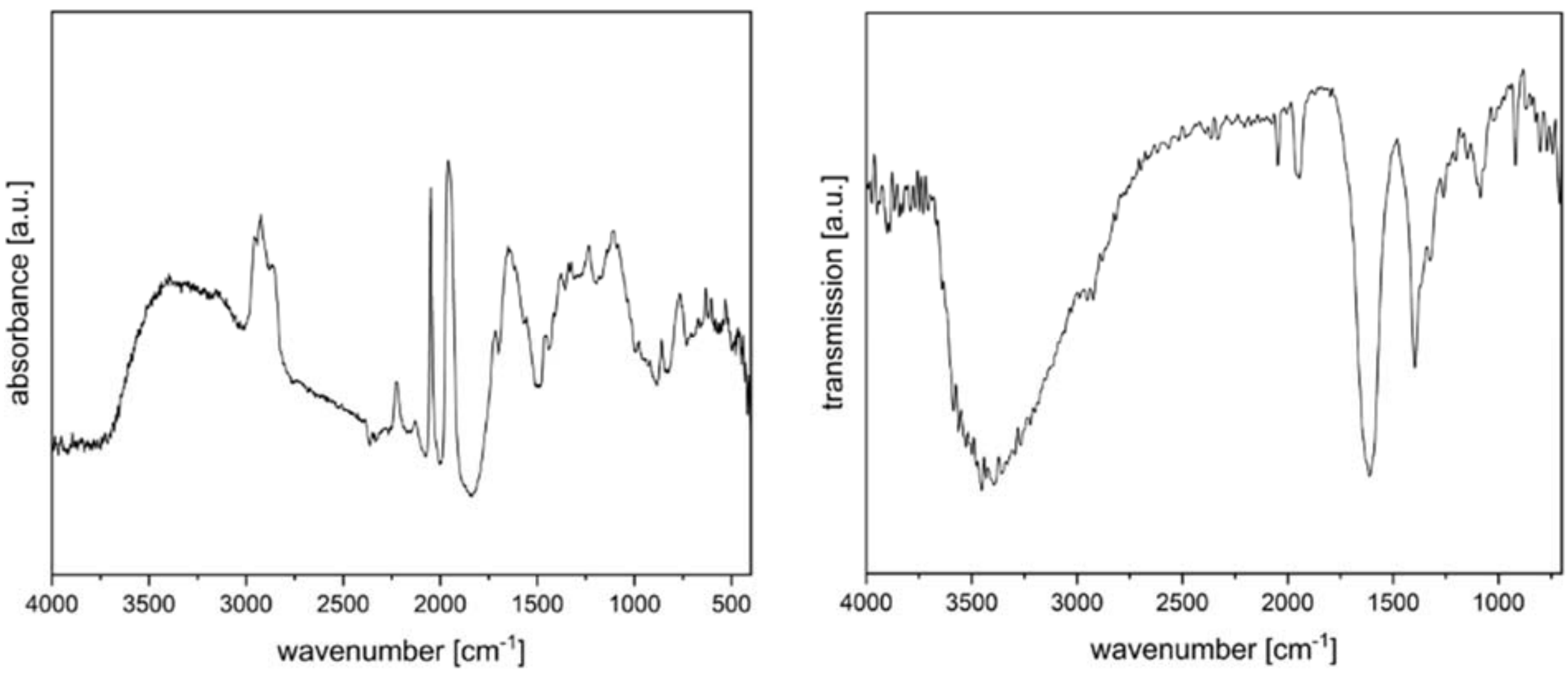

Figure S24. DRIFT spectrum (left) and FT-IR (KBr pellet) spectrum (right) of PhotoCORM functionalized nanodiamond **ND-CORM-anti-EGFR pep**.

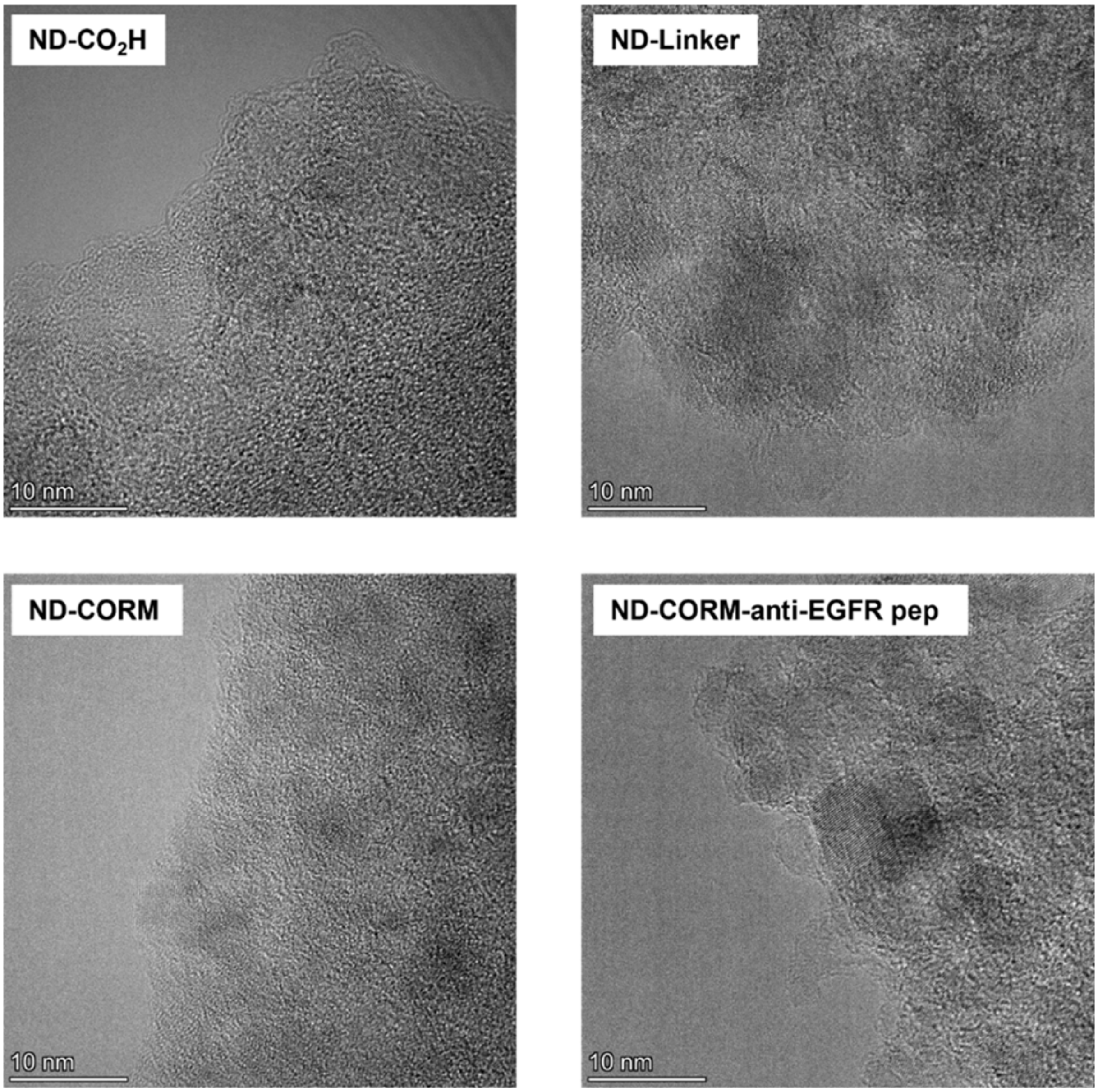

Figure S25. HRTEM images of all produced ND conjugates. Scale bar: 10 nm.

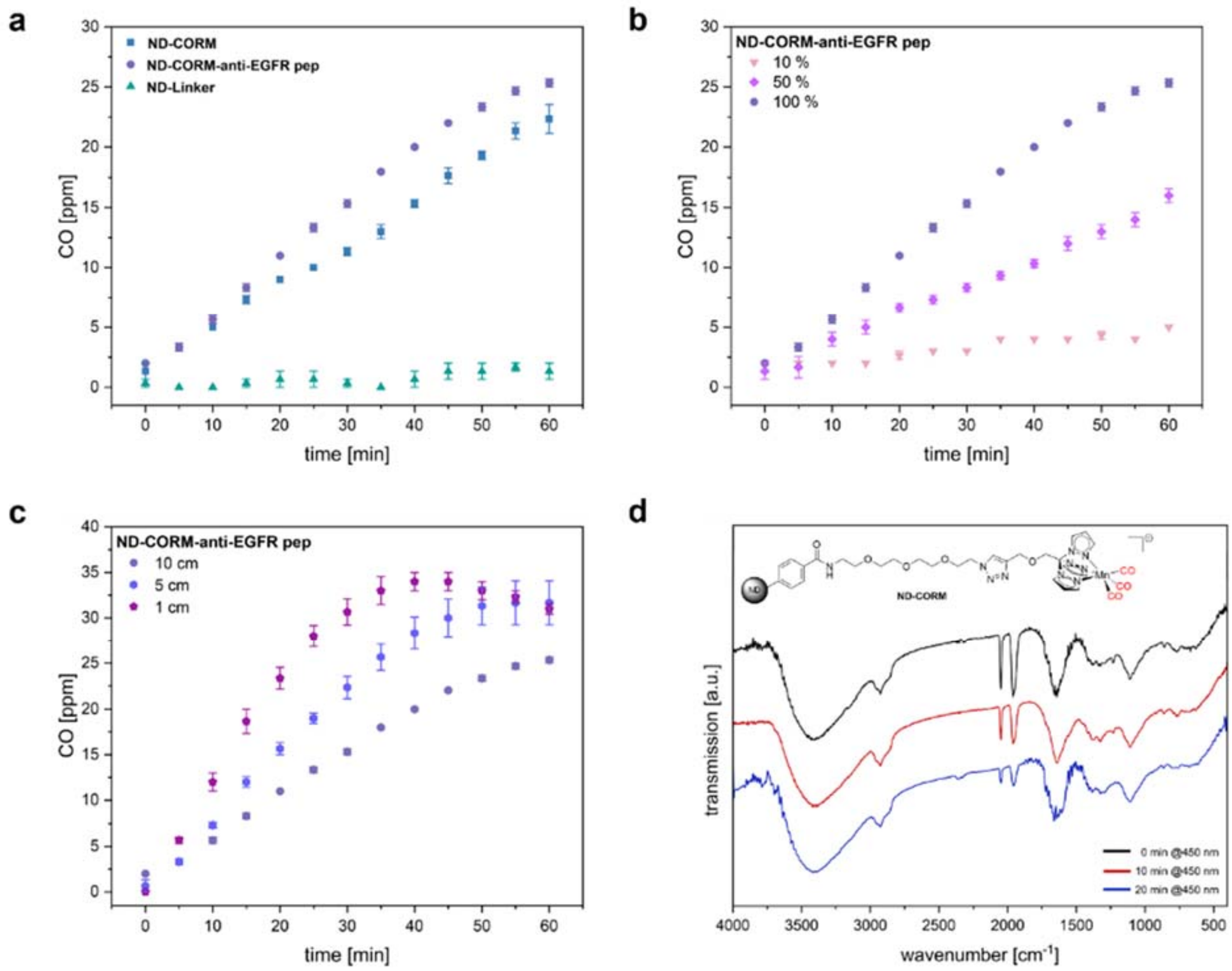

Figure S26. (a) CO release profiles of **ND-CORM**, **ND-CORM-anti-EGFR pep** and **ND-Linker** recorded over the course of 1 h of photoactivation at 450 nm (100 % optical power). Error bars show the standard error of the mean of triplicate measurements. (b) CO release profiles of **ND-CORM-anti-EGFR pep** at 10 % optical power (data points indicated by triangles), 50 % optical power (data points indicated by diamonds), and 100 % optical power (data points indicated by circles) over the course of 1 h of photoactivation at 450 nm. (c) CO release profiles of **ND-CORM-anti-EGFR pep** at 10 cm distance from the LED light source (data points indicated by circles), 5 cm distance (data points indicated by hexagons), and 1 cm distance (data points indicated by pentagons) at 100 % optical power for an illumination period of 1 h. (d) DRIFT spectra of **ND-CORM** recorded in transmission mode after illumination at 450 nm for 0 – 20 min.

4. Additional results from *in vitro* and *in vivo* studies

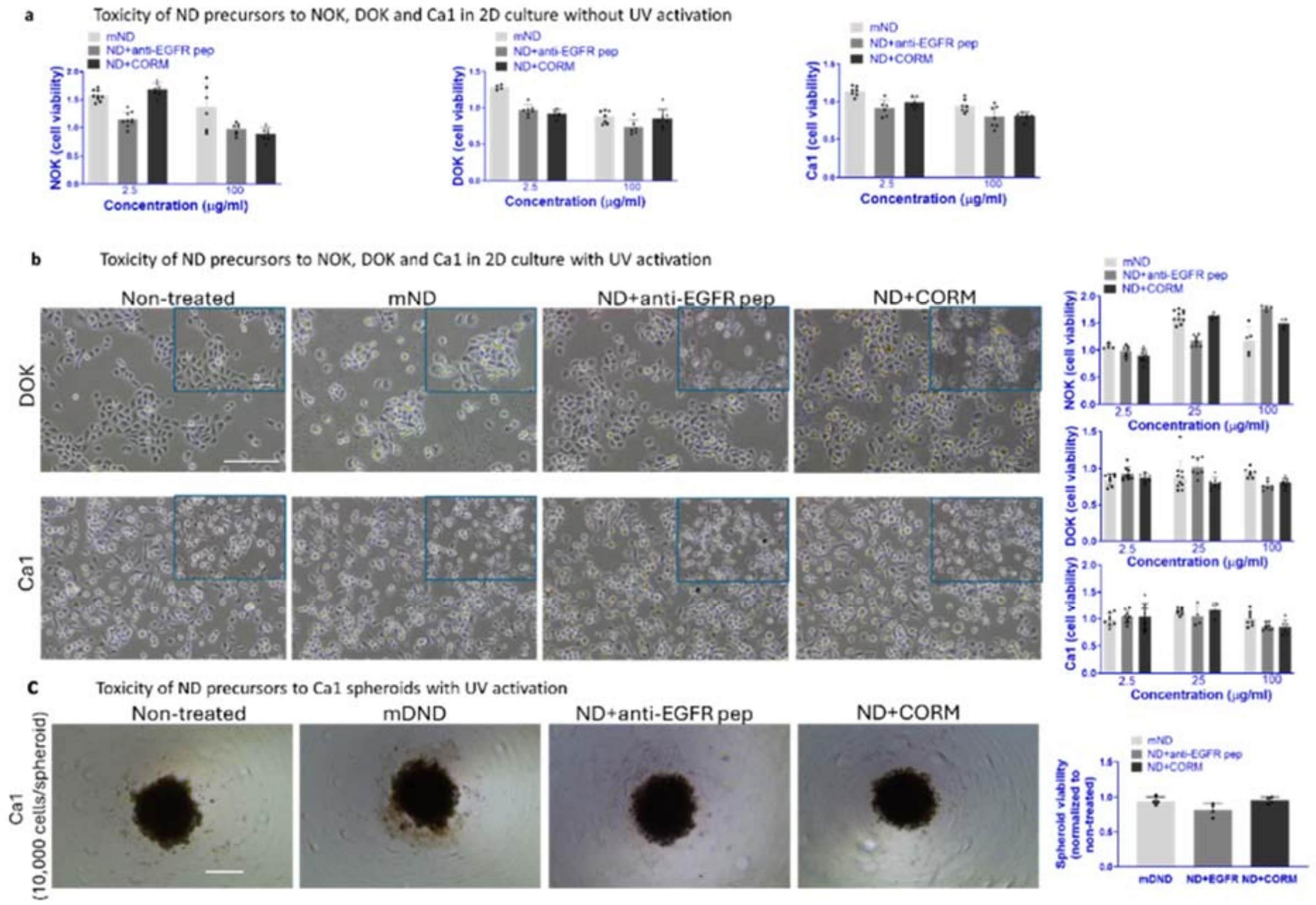

Figure S27. Biocompatibility assessment of functionalized NDs and precursor materials with photoactivation in *in vitro* models. (a) Toxicity of different ND precursors at the lowest and highest tested concentration without photoactivation. Note that the values are normalized to non-treated controls. (b) Brightfield micrographs showing effect of different ND precursors at 25 µg mL^{-1} to DOKs and Ca1. Quantitative cell viability measurements of NOKs DOKs and Ca1 to different precursors at three concentrations with photoactivation. (c) Toxicity of different ND precursors to Ca1 tumor spheroids with photoactivation. Note that NOKs and DOKs, being normal and premalignant cell lines, do not form uniform spheroids.

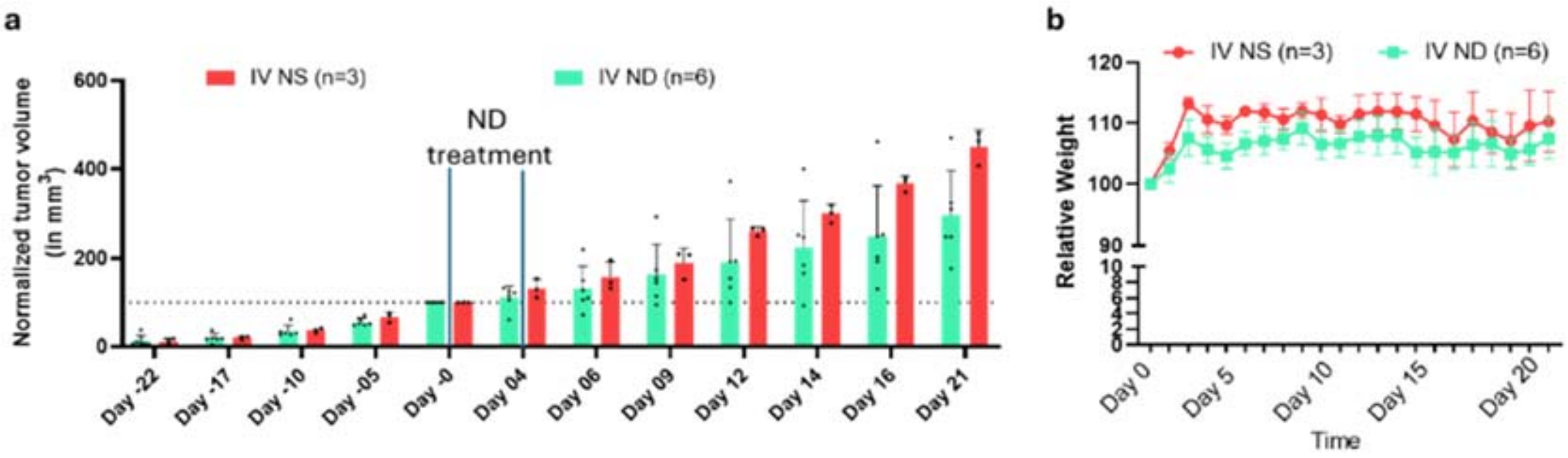

Figure S28. (a) Caliper-measured tumor size over the course of the treatment. At day 23, p=0.09 (b) Body weight measurements showing some differences between intravenous (IV) **ND-CORM-anti-EGFR pep** *vs.* IV PBS injected mice.

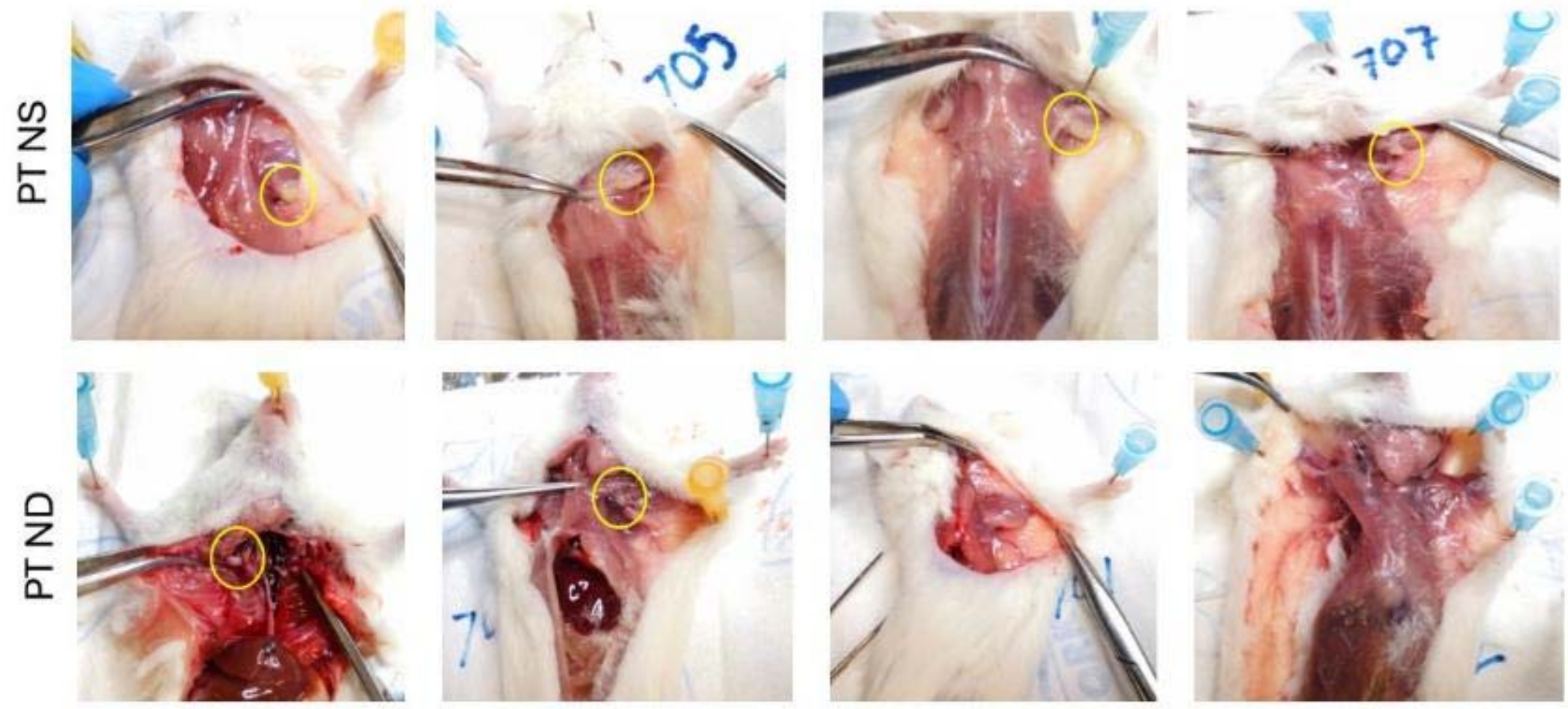

Figure S29. Macroscopic observations of lymph node metastasis in PT NS and PT ND groups. Note: yellow circles indicate presence of macroscopic lymph node metastasis.

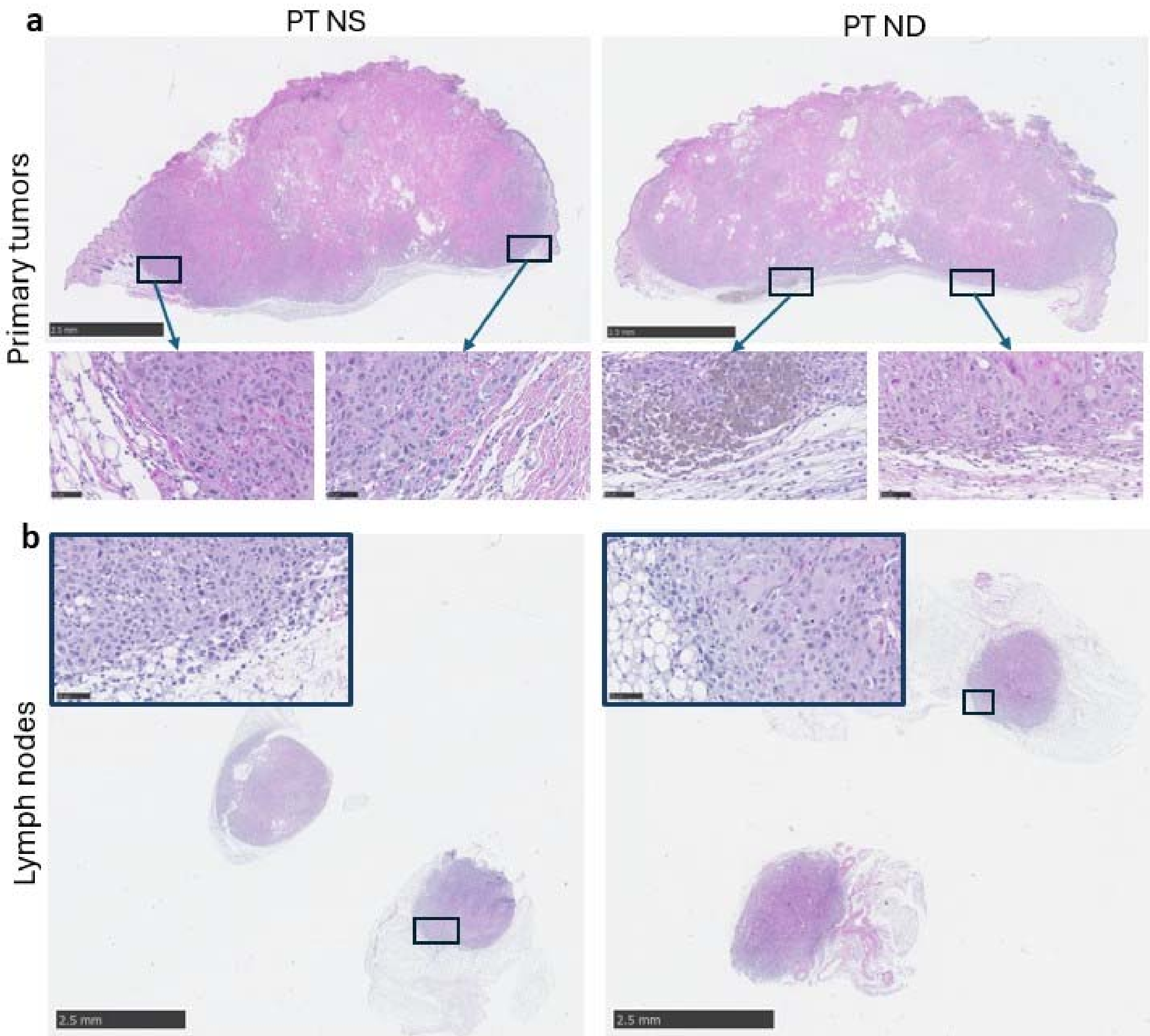

Figure S30. Histological analysis of (a) primary tumors and (b) lymph node metastasis. Scale bar: 2.5 mm. Inset showing magnified image of boxed area. Scale bar: 250 µm.

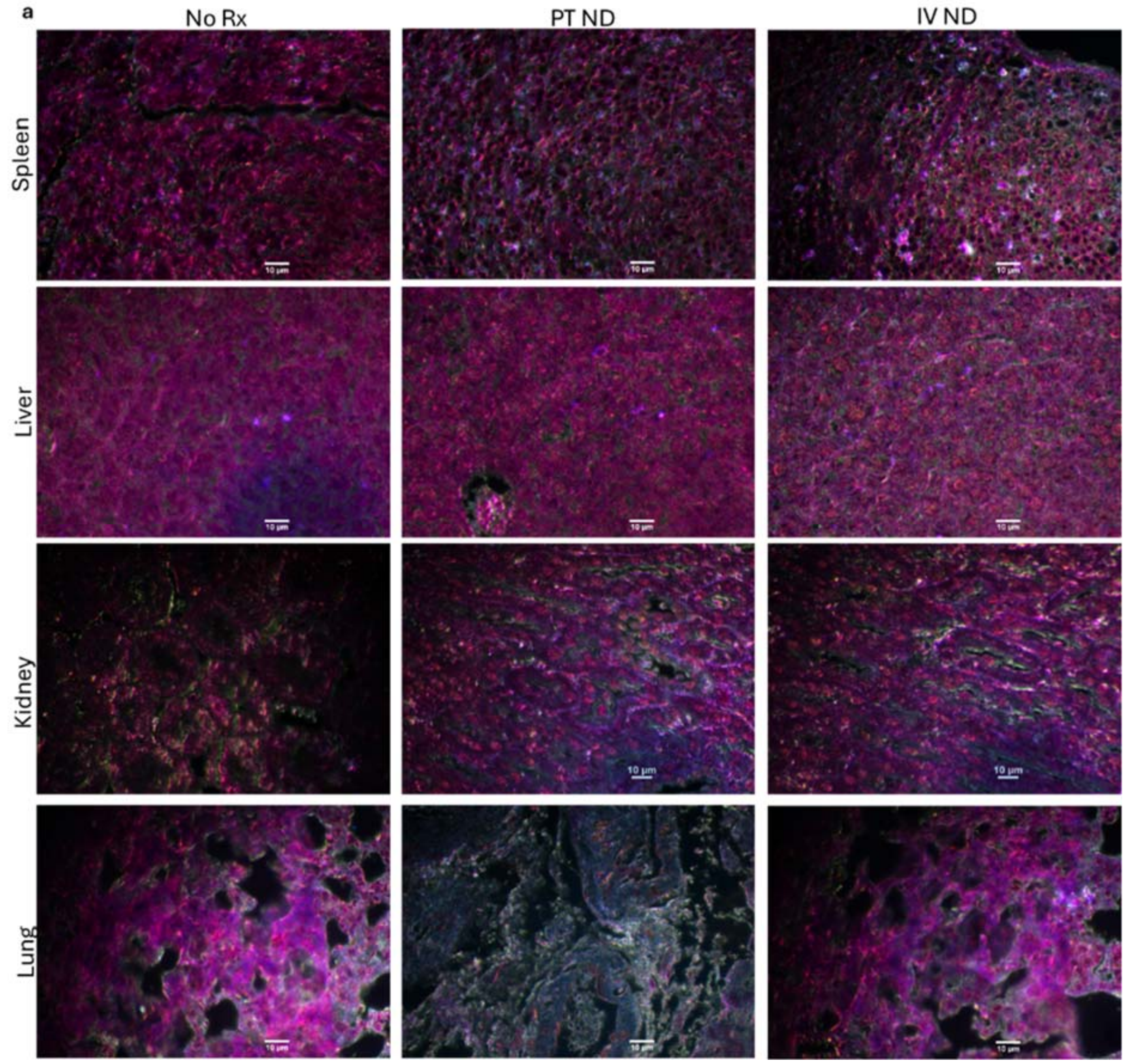

Figure S31. (a) Ultra-high resolution imaging of spleen, liver, kidneys, and lungs of untreated (No Rx), PT treated and IV treated mice showing higher accumulation of NDs (see as bright white spots) in the spleen of IV treated mice.

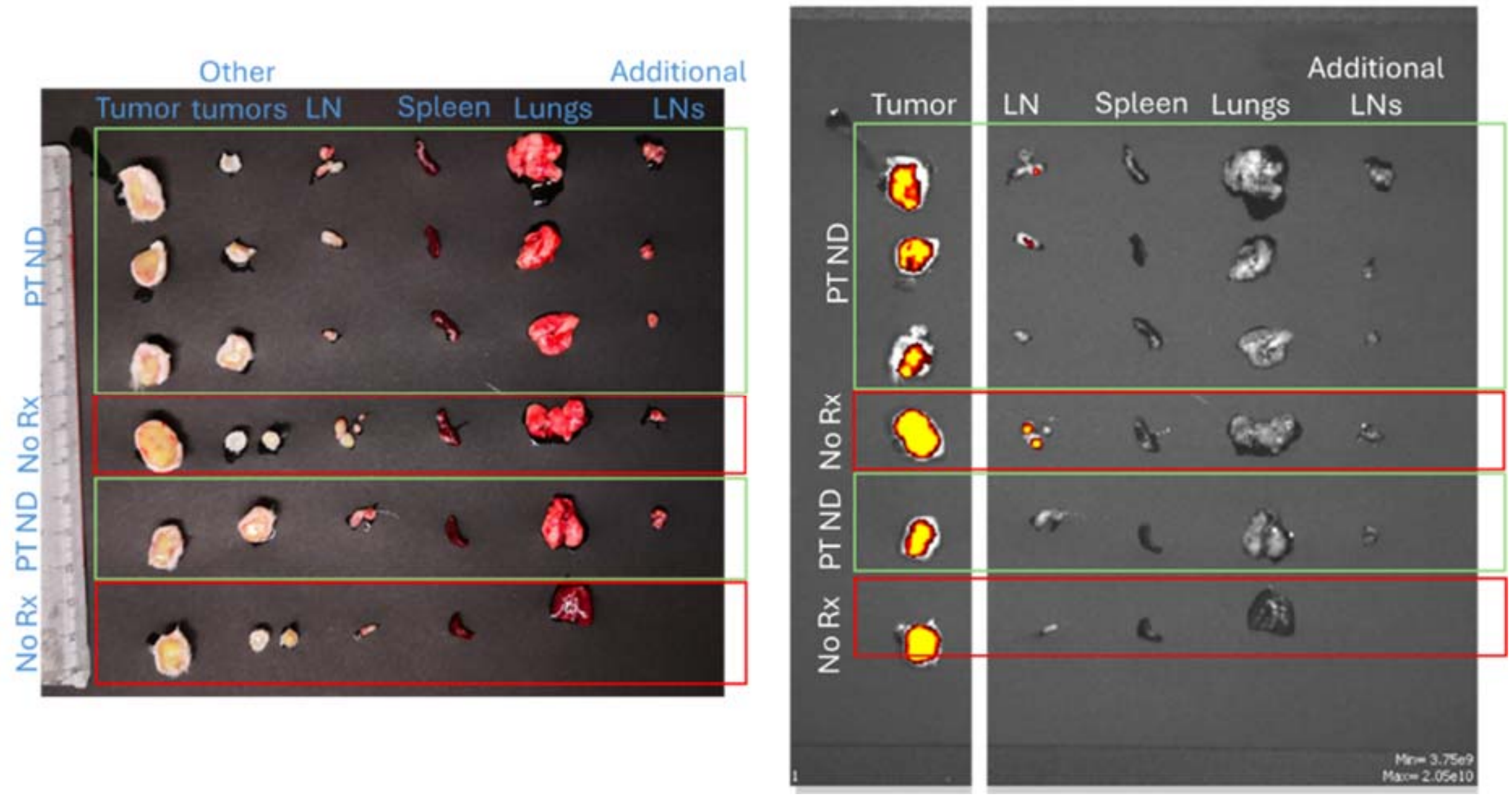

Figure S32. Raw bioluminescence image showing all the excised organs from the PT ND group only.

5. References

[1] K. Brunner, J. Harder, T. Halbach, J. Willibald, F. Spada, F. Gnerlich, K. Sparrer, A. Beil, L. Möckl, C. Bräuchle, K.-K. Conzelmann, T. Carell, *Angew. Chem. Int. Ed.* **2015**, *54*, 1946-1949

[2] V. Merz, J. Lenhart, Y. Vonhausen, M. E. Ortiz-Soto, J. Seibel, A. Krueger, *Small* **2019**, *15*, 1901551.

[3] D. L. Reger, J. R. Gardinier, S. Bakbak, R.F. Semeniuc, U. H. F. Bunz, M. D. Smith, *New J. Chem.* **2005**, *29*, 1035–1043.

[4] H. Pfeiffer, A. Rojas, J. Niesel, U. Schatzschneider, *Dalton Trans.* 2009, 4292–4298.

[5] G. Dördelmann, H. Pfeiffer, A. Birkner, U. Schatzschneider, *Inorg. Chem.* **2011**, *50*, 4362–4367.

[6] G. Dördelmann, T. Meinhardt, T. Sowik, A. Krueger, U. Schatzschneider, *Chem. Comm.* **2012**, *48*, 11528–11530.

[7] G. R. Fulmer, A. J. M. Miller, N. H. Sherden, H. E. Gottlieb, A. Nudelman, B. M. Stoltz, J. E. Bercaw, K. I. Goldberg, *Organometallics* **2010**, *29*, 2176–2179.
